# 3D chromatin organisation contributes to the regulatory logic in differentiating lymphatic endothelium

**DOI:** 10.1038/s44319-026-00895-1

**Published:** 2026-08-14

**Authors:** Virginia Panara, Hannah Arnold, Marleen Gloger, Renae Skoczylas, Victoria Vidal Gutierrez, Anna Johansson, Agata Smialowska, Katarzyna Koltowska

**Affiliations:** 1 Department of Immunology, Genetics and Pathology, Uppsala University, Uppsala, Sweden; 2 Beijer Gene and Neuro Laboratory, Uppsala University, Uppsala, Sweden; 3 Department of Organismal Biology, Uppsala University, Uppsala, Sweden; 4 Institut des Sciences de l’Evolution de Montpellier, University of Montpellier, Montpellier, France; 5 Department of Cell and Molecular Biology, National Bioinformatics Infrastructure Sweden, Science for Life Laboratory, Uppsala University, Uppsala, Sweden; 6 Department of Biochemistry and Biophysics, National Bioinformatics Infrastructure Sweden, Science for Life Laboratory, Stockholm University, Stockholm, Sweden

**Keywords:** Cardiovascular System, Chromatin, Transcription & Genomics, Development

## Abstract

Gene activation and repression is an integral part of embryonic development and tissue formation. Changes in chromatin organisation dictate accessibility to gene regulatory elements, controlling gene expression. Although several molecular regulators of lymphatic endothelial cell (LEC) development have been identified, the role of chromatin organisation in the acquisition of LEC identity remains unclear. In this study, we combine HiC and ATAC-sequencing to map 3D chromatin architecture and accessibility in LECs and blood endothelial cells (BECs). We identify cell type-specific topologically associating domains (TADs), and discovered TAD boundaries changes and differentially segregating enhancers in lymphatic-associated loci, such as *prox1a* and *tbx1*. Our multi-omic approach also defines the regulatory logic of nine LEC-enriched genes. In vivo validation of ATAC- and HiC-based enhancers confirms their activity in LECs. Leveraging these datasets, we reconstructed *mafba* tissue-specific regulatory networks identifying a genetic interaction with *tfe3a* in vivo limiting ectopic vessel formation. Overall, our work provides a powerful resource of multi-omic datasets that can be used to systematically determine the regulatory networks governing LEC identity and genes linked to lymphatic disease.

## Synopsis

The topologically associating domains (TADs) and chromatin accessibility of lymphatic endothelial cells diverge from those of blood endothelial cells. These differences underlie the activation of LEC-specific regulatory networks governing cell identity and involving genes linked to lymphatic disease.

- The LEC and BEC populations present lineage-specific chromatin organisation at the level of both TADs and chromatin accessibility.
- Tissue-specific chromatin structural changes are association with key genes involved in LEC differentiation.
- Novel local enhancers of lymphatic enriched genes are marked by differentially accessible chromatin.
- Tissue-specific chromatin loops connect the promoters of known vascular and lymphatic regulators, such as *mafba* and *tbx1*, with long-range enhancers active in the lymphatic endothelium.
- Transcription factor footprinting revealed an interaction between *mafba* and *tfe3*, which limits ectopic vessel formation and ensures a correct vascular differentiation.

## Abstract (web summary)

The topologically associating domains (TADs) and chromatin accessibility of lymphatic endothelial cells diverge from those of blood endothelial cells. These differences underlie the activation of LEC-specific regulatory networks governing cell identity and involving genes linked to lymphatic disease.

## Graphical abstract

**Figure:**
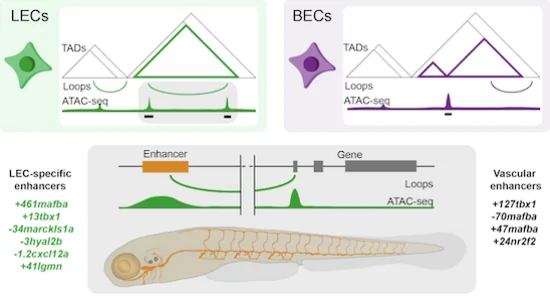

## Introduction

The lymphatic endothelial cells (LECs) originate from different cellular sources (Hu et al, 2024; Lupu et al, 2025). Despite this, LEC identity acquisition is uniformly accompanied by the induction and regulation of lymphatic gene expression. Changes in chromatin structure and enhancer-promoter interactions are key in controlling gene expression in a cell type-specific manner and serve as an integral regulatory mechanism. However, the mechanisms by which chromatin organisation defines the uniform molecular signature in LECs remain to be understood.

During embryonic development, a pool of lymphatic progenitors gives rise to a lymphatic vascular network that extends across the body. The establishment of this complex and diverse system of vessels depends on steps such as specification, proliferation, migration and differentiation. These processes are regulated by molecular mechanisms which ensure the morphology and functionality of the network. The lymphatic vessel progenitors are distinguished from blood endothelial cells (BECs) by expression of Prox1, a transcription factor (TF) necessary for lymphatic vessel formation in mice and zebrafish (Koltowska et al, 2015a; Wigle et al, 1999). In mice, *Prox1* expression is directly regulated by Sox18 and CoupTFII (François et al, 2008; Srinivasan et al, 2010). Both in mice and zebrafish, defined functional lymphatic enhancers contribute to the upstream regulation of *Prox1* (Kazenwadel et al, 2023; Panara et al, 2024).

A key pathway regulating LEC sprouting, specification, proliferation and migration is Vegfc-Vegfr3 signalling. This pathway has an important role in controlling Prox1 expression. In zebrafish, Prox1 is induced by Vegfc-Vegfr3 signalling (Koltowska et al, 2015a), and in mice, a Vegfc-Vegfr3 feedback loop regulates the maintenance of Prox1 (Srinivasan et al, 2014). In addition to this role, Vegfc-Vegfr3 signalling also mediates the migration of specified LEC progenitors across the embryo through its downstream components Mafba and Mafbb (Arnold et al, 2022; Koltowska et al, 2015b). Vegfr3 expression itself is regulated by the TFs Tbx1 and Hhex (Chen et al, 2010; Gauvrit et al, 2018). Additional transcription factors, such as Gata2, Foxc2, Foxp2 and Nfatc1, have a described role in the maturation of lymphatic vessels and in lymphatic valve formation (Hernández Vásquez et al, 2021; Kazenwadel et al, 2015; Norrmén et al, 2009; Petrova et al, 2004). Despite the identification of these TFs and signalling pathways as key factors in lymphatic development, the LEC transcriptional network and downstream factors have not been systematically defined.

Central to the regulation of gene expression and the shifts in transcriptomic profiles necessary for cell differentiation and tissue formation are chromatin organisation and enhancer-promoter interactions (Ruiz-Velasco and Zaugg, 2017). In cells, differential gene expression is regulated by chromatin structural changes that enable DNA-binding proteins, including TF, to access regulatory sequences and interact with gene promoters. Changes in chromatin conformation can occur both at the local level, by the opening or closing of chromatin, and distally, by the formation of chromatin loops connecting distant regions of DNA, such as promoters and their regulatory elements. The loops are further organised in Topologically Associating Domains (TADs), which are restricted by insulators to confine chromatin interactions (Mañes-García et al, 2024). Chromatin architectural changes during lymphatic development have not been explored. Therefore, it is compelling to determine if loop restructuring is one of the molecular mechanisms that contribute to the LEC differentiation.

In this study, we have characterised the chromatin organisation of LECs and its associated transcriptional signature using a combination of omics approaches. We uncovered TADs, DNA loops and enhancers specific to the LEC population, providing a more holistic view of the regulatory signature of differentiated LECs. By in vivo validation, we have identified active ATAC- and HiC-based enhancer sequences driving reporter expression in LECs. To assess the functional relevance of our data, we have reconstructed the transcriptional network downstream of the known lymphatic TF Mafb. We further validated its genetic interaction with Tfe3a in vivo, determining its necessity for regulating ectopic vessel formation. These results show that the acquisition of LEC identity is marked not only by the establishment of a cell type-specific transcriptional signature, but also by a matching structural change of chromatin organisation.

## Results

### The LEC and BEC populations present lineage-specific chromatin organisation

**Figure 1:**
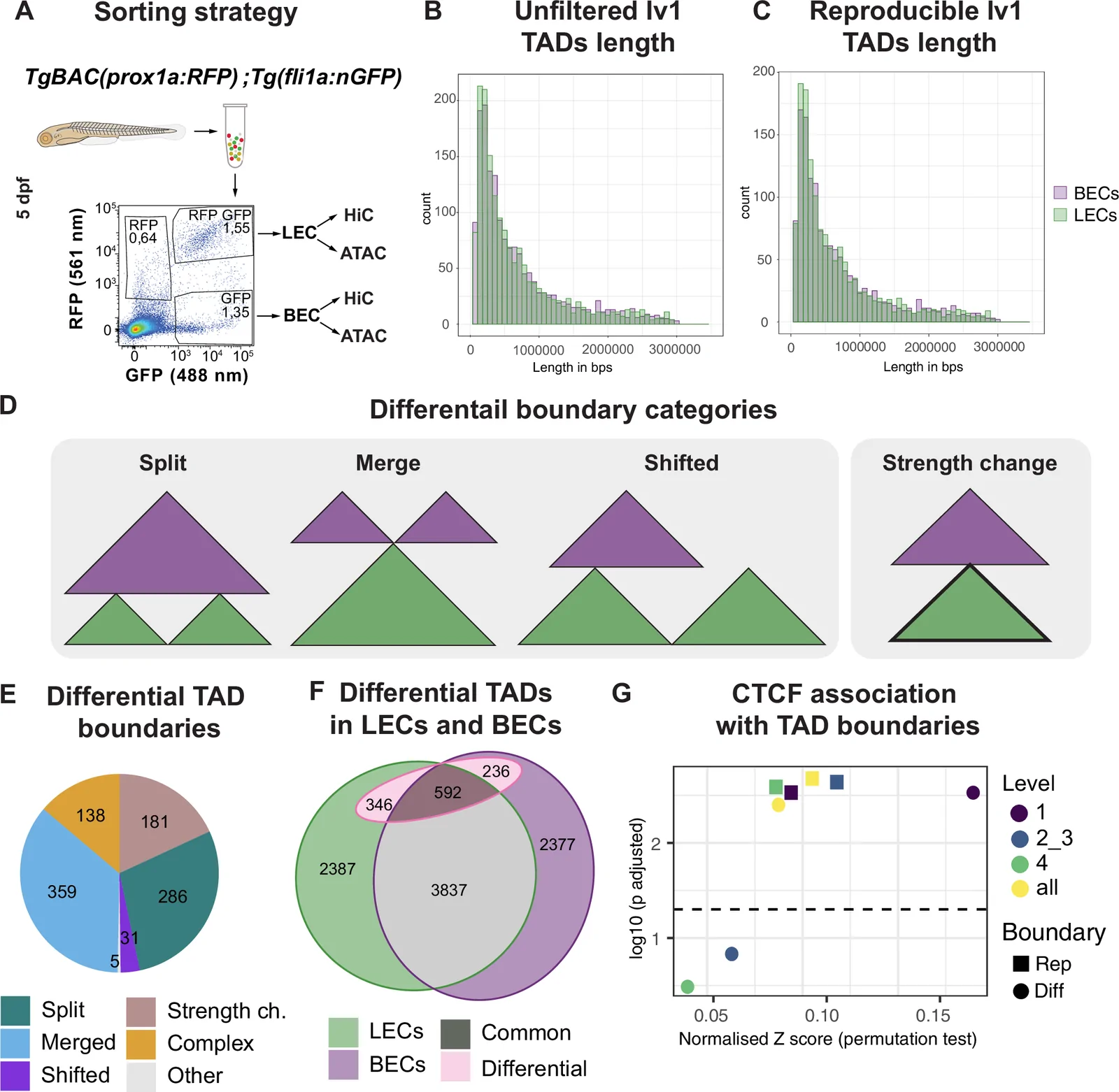
TAD structure differs between LECs and BECs. (**A**) Cell sorting strategy for HiC and ATAC-seq. ATAC-seq was performed in biological triplicates and HiC in duplicates. LEC and BEC were isolated from *Tg(fli1a:nEGFP); Tg (prox1a: RFP)* zebrafish at 5 dpf. (**B**,** C**) Length distribution of unfiltered (**B**) and reproducible (**C**) level 1 TADs in LECs and BECs. (**D**) TADcompare classification scheme (adapted from Cresswell and Dozmorov, 2020). (**E**) Distribution of reproducible differential boundaries across TADcompare categories. (**F**) Summary of reproducible differential TADs across all TAD levels. Green: reproducible TADs detected in LECs; purple: reproducible TADs detected in BECs; pink: differential TADs. (**G**) Results of permutation tests for overlaps of TAD boundaries and CTCF occupancy at 48 h from (Franke et al, 2021). 1: outermost TADs; 2_3: intermediate inner TADs; 4: TADs deeper in hierarchy; square: reproducible boundaries; circle: differential boundaries. Significance of overlaps with CTCF peaks was assessed by permutation tests. The horizontal line represents a significance cutoff *p* = 0.05.

**Figure EV1:**
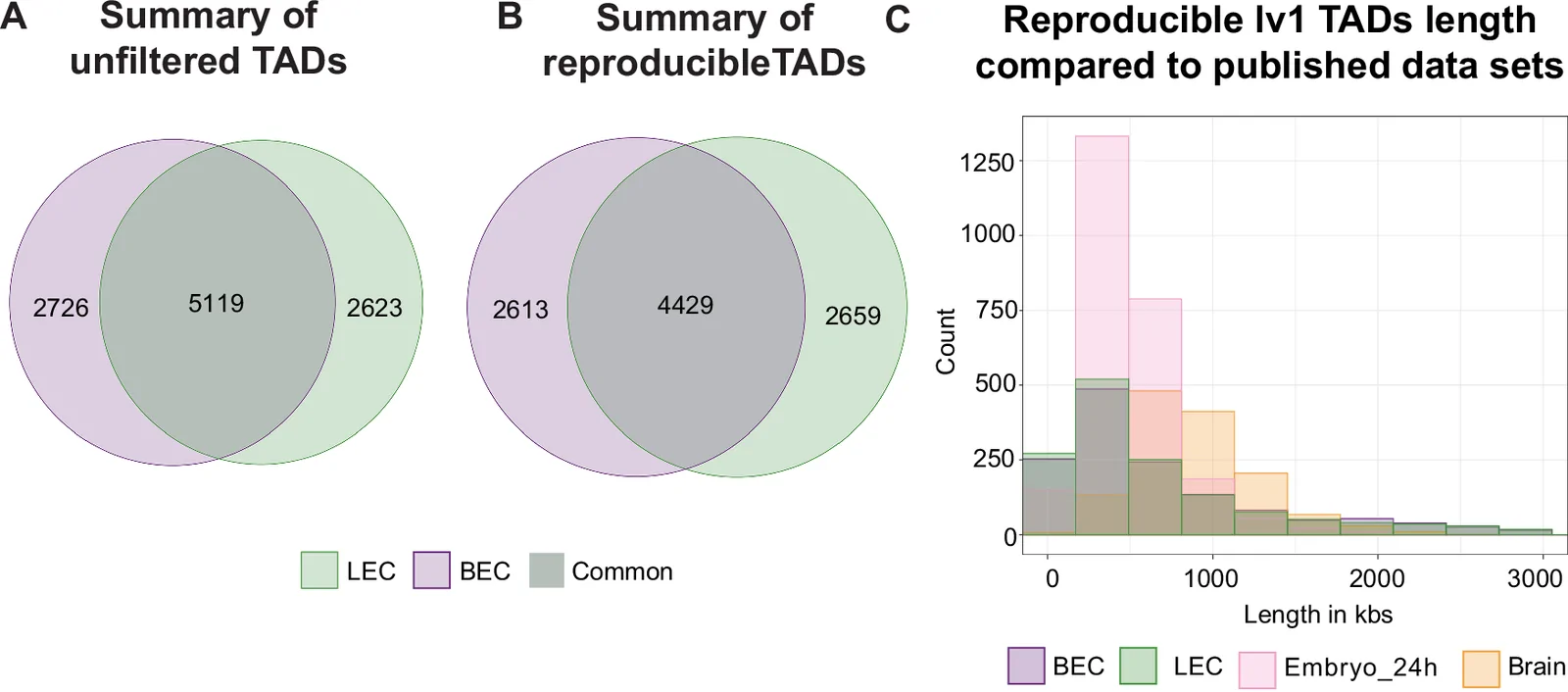
TAD structure differs between LECs and BECs. (**A**) Summary of unfiltered TADs of all levels in LECs and BECs. (**B**) Summary of reproducible TADs of all levels in LECs and BECs. (**C**) Length distribution of level 1 TAD compared with published datasets, showing similarities to reported brain data. Purple: BEC TADs; Green: LEC TADs; pink: 24 hpf embryo TADs from (Franke et al, 2021); yellow: brain TADs from Yang et al, (2020)

To understand how chromatin is organised in lymphatic endothelial cells (LECs) and if it presents unique features compared to blood endothelial cells (BECs), we have used an HiC sequencing approach to compare these two cell populations. We isolated LECs and BECs using Fluorescent Activated Cell Sorting (FACS) and sorted cells from *Tg(fli1a:nEGFP)*^*y7*^*;TgBAC(prox1a:KalTA4-4xUAS-ADV.E1b:TagRFP)*^*nim5*^ (hereafter referred to as *Tg(prox1a:RFP)*^*nim5*^) embryos at 5 days post-fertilisation (dpf). Double-positive *Tg(fli1a:nEGFP)*^*y7*^; *Tg(prox1a:RFP)*^*nim5*^ cells were enriched for LECs, while single *Tg(fli1a:nEGFP)*^*y7*^ cells were enriched for BECs, which include both arterial and venous endothelial cell (VEC) populations (Fig. 1A). Sequencing of 50,000 cells per sample from two replicates identified, on average, 300 M number of chromatin contacts per sample (read pairs after filtering duplicates, intra-fragment pairs, low-quality alignments). A third replicate was excluded from the analysis due to insufficient sequencing depth. TAD boundaries were called on merged replicates using OnTAD (An et al, 2019), a nested TAD caller. TAD boundaries from all levels in the TAD hierarchy were interrogated for differential contact frequency using TADCompare (Cresswell and Dozmorov, 2020). Reproducible TAD boundaries in LEC and BEC, respectively, were identified by comparing contact frequency between replicates (Datasets EV1 and 2). Boundaries not identified as differential between replicates were considered tissue-reproducible. Reproducible TADs were delimited by two reproducible boundaries. We found that LECs and BECs have a similar distribution and number of TADs at all hierarchical levels before and after filtering for reproducible ones, with around 25% of TADs being specific for both LECs and BECs (Fig. EV1A,B). Similarly, the length distribution of outermost (level 1) TADs before and after filtering shows no marked differences (Fig. 1B,C), confirming unbiased filtering. We further validated the TADs prediction by TADs length comparison with previously published zebrafish datasets, in dissected brain (Yang et al, 2020; Data ref: Yang et al, 2020) and whole embryo at 24 h post-fertilisation (hpf) (Franke et al, 2021). In our samples, we could observe similar TADs characteristics to those in the dissected tissue data (Fig. EV1C).

To characterise the differences in TAD organisation between LECs and BECs, differential TAD boundaries between LECs and BECs were identified by comparing the signal in merged replicate matrices and classified based on published criteria (Cresswell and Dozmorov, 2020) (Fig. 1D). Reproducible TADs delimited by at least one differential boundary in LECs vs. BECs were considered. Overall, 1000 differential boundaries were identified between LECs and BECs, of which 138 were classified as 'complex' changes, 645 as “merged/split”, 31 as “shifted”, and 186 as “strength” changes” or unclassified (Fig. 1E; Dataset EV3). To be able to compare TADs between LECs and BECs, we defined common TADs as delimited by both boundaries within 30 kb distance in both tissues. In total, both LECs and BECs contain around 2500 TADs that were unique to their subtype, while 4429 were common to both cell populations. Out of these, differential TAD boundaries were found in association with 272 LEC TADs, 236 BEC TADs and 592 common TADs (Fig. 1F). We then asked whether published CTCF ChIP-seq peaks are associated with common and differential TAD boundaries in LECs and BECs to underscore their structural relevance.

To do so, we leveraged the previously published dataset of CTCF occupancy in whole embryos at 48 hpf (Franke et al, 2021). The associations between reproducible and differential TAD boundaries and regions of CTCF occupancy were evaluated using permutation tests. Boundaries were stratified into three groups: level 1, including the outermost TADs; levels 2 and 3, including intermediate inner TADs; and level 4, including TADs deeper in the TAD hierarchy, of level 4 or higher. As expected, the boundaries of reproducible TADs show a strong association with CTCF occupancy regardless of hierarchical level (Fig. 1G). However, we observed that the boundaries of deeper-level differential TADs are not significantly associated with peaks of CTCF occupancy at 48 hpf. This might suggest that these tissue-specific TADs emerge later during development, accompanying the acquisition of mature LEC and BEC identities.

Overall, these data reveal that chromatin organisation presents localised differences between LECs and BECs, as shown by the contrasting differential TAD structure of these cell populations.

### The chromatin organisation shows structural changes in loci of genes enriched in LECs and VECs

**Figure 2:**
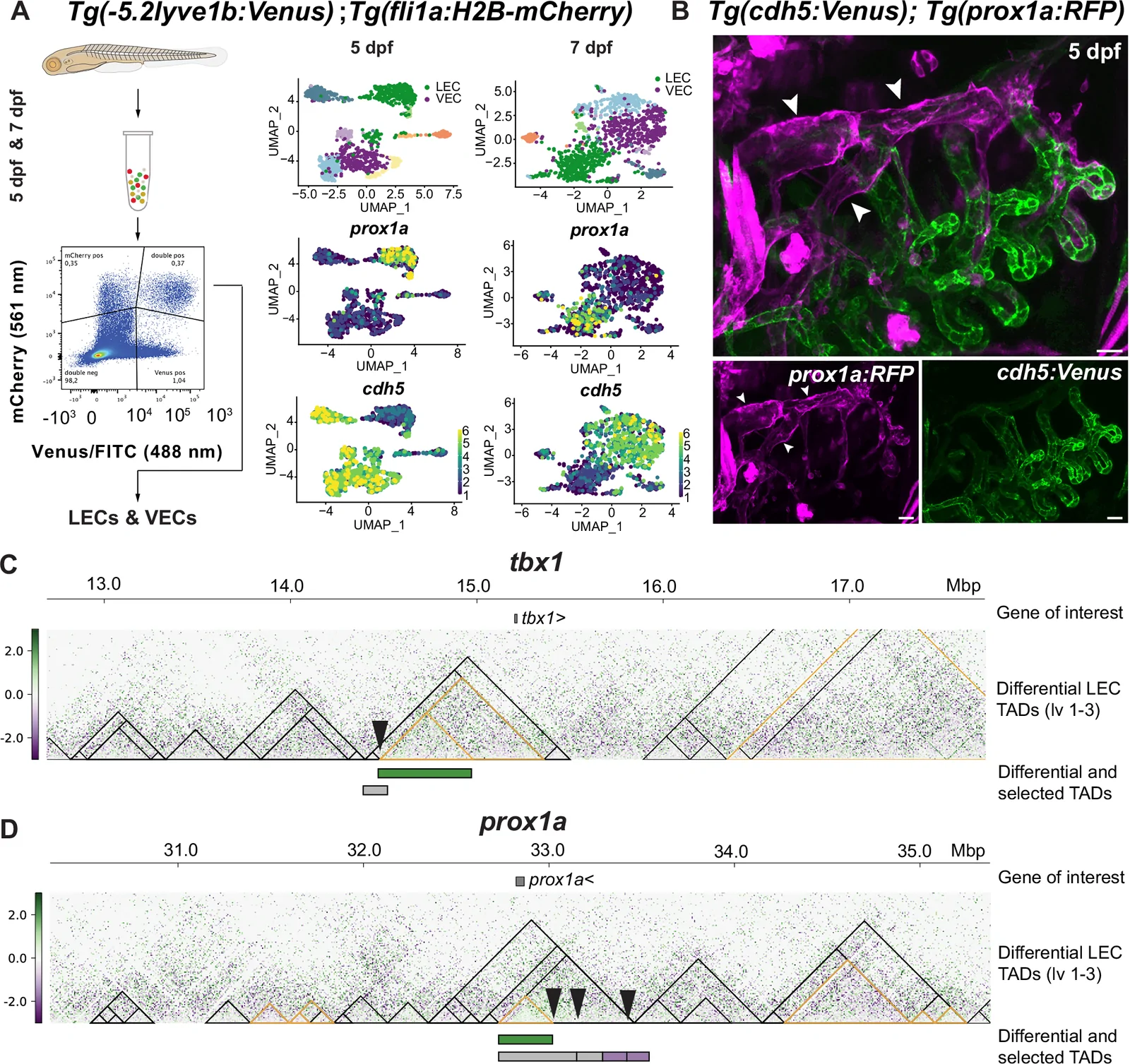
Different chromatin signatures are associated with known lymphatic factors in LECs and BECs. (**A**) Schematic of LEC and VEC isolation from *Tg(-5.2lyve1b:Venus)* and *Tg(fli1a:H2B-mCherry)* zebrafish for scRNA-seq data (top) and identification of the main LEC and VEC clusters with normalised expression of the lymphatic marker *prox1a* and venous marker *cdh5* at 5 dpf and 7 dpf (bottom; Viridis scale). (**B**) Expression of *Tg*(*cdh5:Venus*) in the blood endothelium (green) and *Tg(prox1aRFP)* in the lymphatic endothelium (magenta, white arrowhead). Scale bars: 20 µm. (**C**,** D**) TAD organisation at the *tbx1* (**C**) and *prox1a* (**D**) loci. Log2 of the HiC signal in LEC vs BEC is shown. Non-differential LEC TADs of level 1–3 are plotted in black and differential LEC TADs in orange. Green boxes: differential TADs in LECs. Purple boxes: differential TADs in BECs. Grey boxes: TADs spanning a differential TAD boundary. Arrowhead: differential boundaries closely associated with the gene of interest. Source data are available online for this figure.

**Figure EV2:**
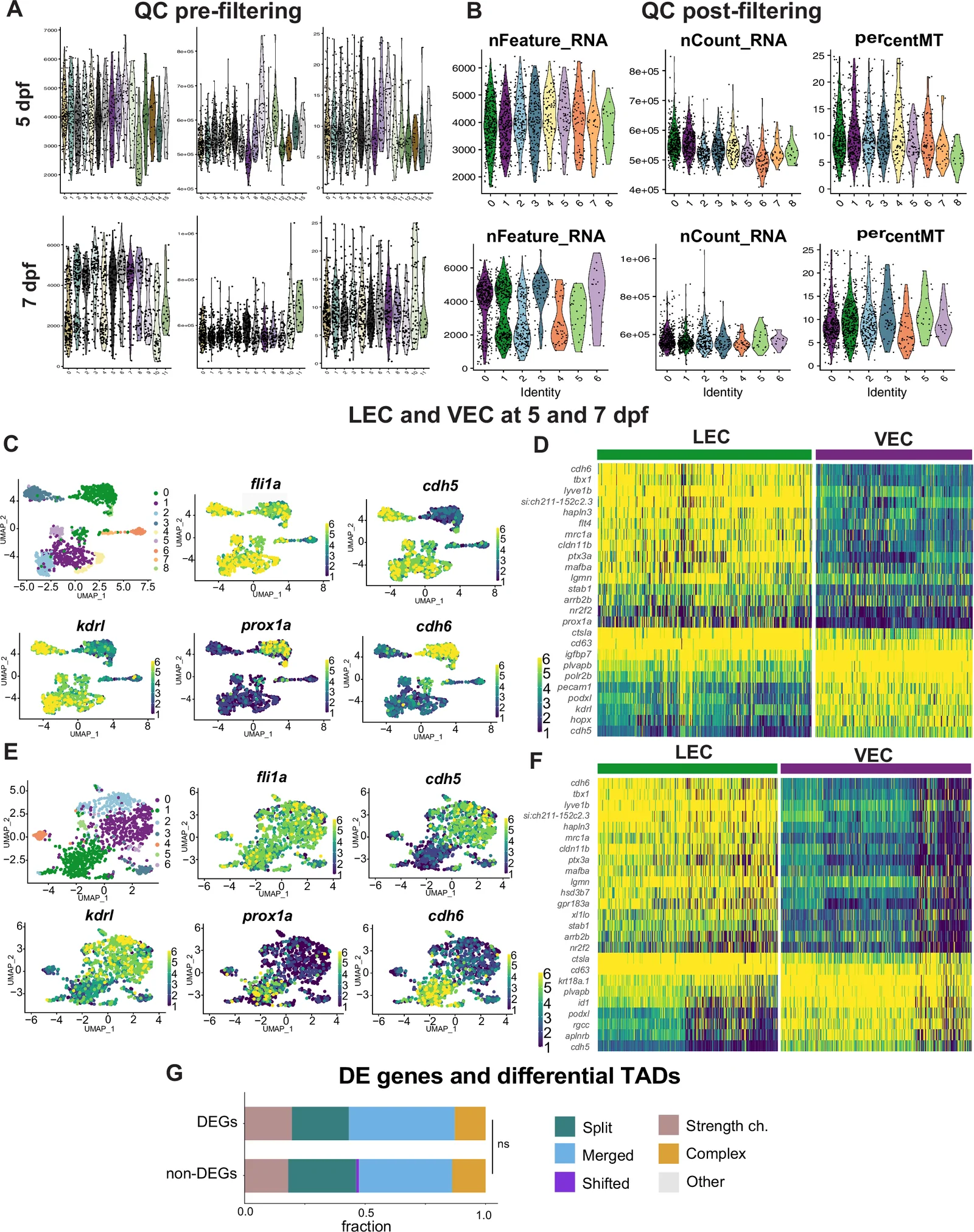
scRNA-seq quality control and cluster identification. (**A**,** B**) Plate quality control (QC) before (**A**) and after (**B**) filtering for low-quality reads, showing the percentage of mitochondrial, protein-coding and rRNA reads. (**C**,** E**) UMAP visualisation of vascular clusters from 5 dpf (**C**) and 7 dpf (**E**), coloured by cluster identity. UMAP plot shows expression of vascular markers: *fli1a* (all endothelium), *chd5* and *kdrl* (blood endothelium), *cdh6* and *prox1a (*lymphatic endothelium). Colour scale shows log-normalised expression. (**D**,** F**) Heatmaps of known gene expression in the endothelial cells at 5 dpf (**D**) and 7 dpf (**F**). Cells ordered by clusters. Colour scale shows log-normalised read counts. (**G**) Distribution of differential TAD boundaries in differentially expressed vs non-differentially expressed genes within level 1 TADs containing a differential boundary. DEG-associated boundaries: *n* = 157; non-DEG-associated boundaries: *n* = 1186. Chi-squared test, ns no significance (*p* = 0.2424). Source data are available online for this figure.

To obtain a genome-wide understanding of which genes with LEC-enriched expression undergo changes in loci chromatin conformation, we have characterised the transcriptional signature of LECs and venous endothelial cells (VECs), the subset of BECs more closely related to LECs, by single-cell (sc) RNA-sequencing. As LECs and VECs share several genes and developmental dynamics, this allowed us to focus on the transcriptomic differences between these cell populations without the stronger gene signature coming from arteries. These data are a subset of those published in (Data ref: Gloger et al, 2026; Koltowska and Gloger, 2026). We used FACS to isolate a mixed LEC and VEC population by sorting double-positive *Tg(-5.2lyve1b:Venus)*^*uu1kk*^ and *Tg(fli1a:H2B-mCherry)*^*uq37bh*^ cells at 5 dpf and 7 dpf (Fig. 2A). To refine the dataset, we used QC filtering and unsupervised clustering (Fig. EV2A,B), which resulted in nine clusters at 5 dpf and seven clusters at 7 dpf (Figs. 2A and EV2C–E). We identified the main LEC cluster at 5 and 7 dpf by the expression of well-characterised LEC genes, such as *prox1a*, *mafba* and *cdh6* (Figs. 2A and EV2C–F; Datasets EV4 and 5). The main VEC cluster was determined by the expression of genes enriched in BECs, such as *cdh5* and *kdrl* (Figs. 2A and EV2C–F; Datasets EV4 and 5). Further comparisons between LEC and VEC clusters showed enriched expression of *flt4*, *lyve1b* and *tbx1* in the former, whereas expression of *podxl* and *aplnrb* was enriched in the latter. In addition, *fli1a* is expressed in all the clusters, confirming their endothelial identity (Figs. 2A and EV2C–F; Datasets EV4 and 5). To validate the assigned LEC and VEC clusters, we used transgenic lines reporting the expression of *cdh5* (*TgBAC(ve-cad:ve-cadTS)*^*uq11bh*^) and *prox1a* (*Tg(prox1a:RFP)*^*nim5*^) and confirmed that within the endothelium *prox1a* is restricted to lymphatics and *cdh5* to blood endothelium (Fig. 2B), further validating the identity of the clusters.

We leveraged the scRNA-sequencing data to link the transcriptomic signature of VECs and LECs with the observed changes in chromatin organisation. We quantified the number of genes with LEC- or VEC-biased expression associated with a changing TAD boundary. A gene was considered as LEC or VEC enriched when the |log2FC| ≥0.3 and the adjusted *p* value <0.1. Genes potentially affected by a change in TAD boundaries are not only the ones overlapping the boundary itself, but also those located in its proximity, as the overall chromatin conformation of the region may be altered. Therefore, this analysis included genes located within differential TADs. The distribution of TADs delimited by differential boundaries in the proximity of differentially expressed genes showed no preferential association with LEC or VEC-enriched loci (Fig. EV2G). In total, we identified 46 LEC-enriched and 42 VEC-enriched genes associated with changing boundaries, corresponding respectively to 14 and 13% of all DE genes (Dataset EV6). This suggests that while not all differences in gene expression are associated with a change in TAD structure, these changes play an important role in regulating tissue-specific gene expression.

We then focused on two transcription factors identified by this analysis, which have known functions in lymphatic endothelial cells: Tbx1 (Chen et al, 2010) and Prox1a (Koltowska et al, 2015a; Wigle et al, 1999). The *tbx1* locus is associated with a differential boundary, resulting in the inclusion of an additional 150 kbp in the outermost TAD encompassing the gene in LECs (Fig. 2C). Similarly, three differential TAD boundaries are closely associated with the *prox1a* locus, resulting in a large 1100 kbp outermost TAD encompassing the gene in LECs (Fig. 2D). This indicates that tissue-specific chromatin structural changes are association with key genes involved in LEC differentiation, and may help to restrict their expression to this cell population.

### Identification of candidate lymphatic regulators based on cell type-enriched gene expression and chromatin accessibility

**Figure EV3:**
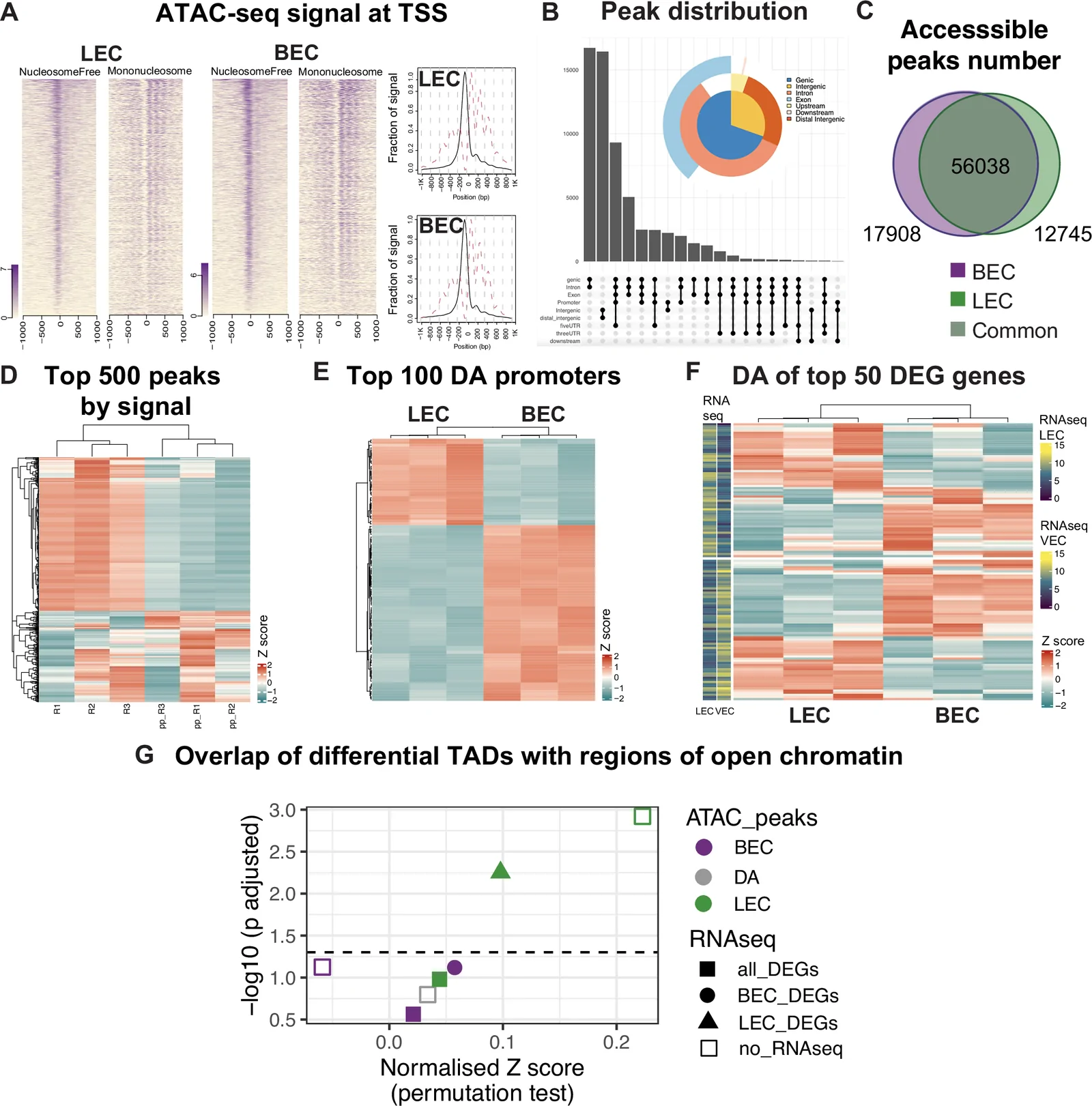
Combination of ATAC-sequencing and scRNA-sequencing reveals novel LEC factors. (**A**) ATAC-seq signal at TSS in nucleosome-free and mononucleosome fractions. Left: heatmaps of scaled counts in 10 bp bins centred on TSS. Right: cumulative TSS signal. (**B**) Distribution of genomic feature annotation of identified ATAC-seq peaks. (**C**) Quantification of the tissue-specific and common accessible ATAC-seq peaks. (**D**) ATAC-seq heatmap showing chromatin state at the top 500 DA peaks. (**E**) ATAC-seq heatmap of the top 100 differentially accessible (DA) promoters at 5 dpf. (**F**) ATAC-seq heatmap of promoters of differentially expressed (DE) genes at 5 dpf. (**G**) Graphical representation of the results of the permutation tests for the association of TADs, open chromatin regions and differential gene expression. Purple: BEC DA regions; green: LEC DA regions, grey: all DA regions; square: regions associated with any DEG; circle: regions associated with BEC DEG, triangle: regions associated with LEC DEGs, empty square: all accessible regions regardless of DEG association. The significance of these overlaps was assessed by permutation tests, where TAD regions were randomised. The horizontal line represents a significance cutoff *p* = 0.05.

In addition to large-scale chromatin organisation, local changes in chromatin accessibility in LECs and BECs were also characterised. We performed bulk ATAC-sequencing of isolated LECs and BECs from 5 dpf zebrafish, ensuring comparability between the datasets by using the same sorting strategy as for the HiC data (Fig. 1A). We confirmed the high quality of the data by plotting the signal profiles of nucleosome-free and mononucleosome fractions of the data at annotated transcriptional start sites (TSS) (Fig. EV3A). The peak aggregation in relation to TSS showed a standard distribution, and the expected genomic features were detected within the open regions (Fig. EV3B), validating the quality of the dataset. We observed that, out of 86,691 peaks, 17,908 had reduced accessibility in LECs compared to BECs, and 12,745 had increased accessibility at FDR cut-off 0.05 (Fig. EV3C; Dataset EV7). Using this data, we identified the top 500 differentially accessible peaks (Fig. EV3D; Dataset EV8) and the top 100 promoters (defined as regions up to 3 kb upstream of the TSS) (Fig. EV3E; Dataset EV9), showing the presence of differences in the chromatin accessibility between LECs and BECs.

**Figure 3:**
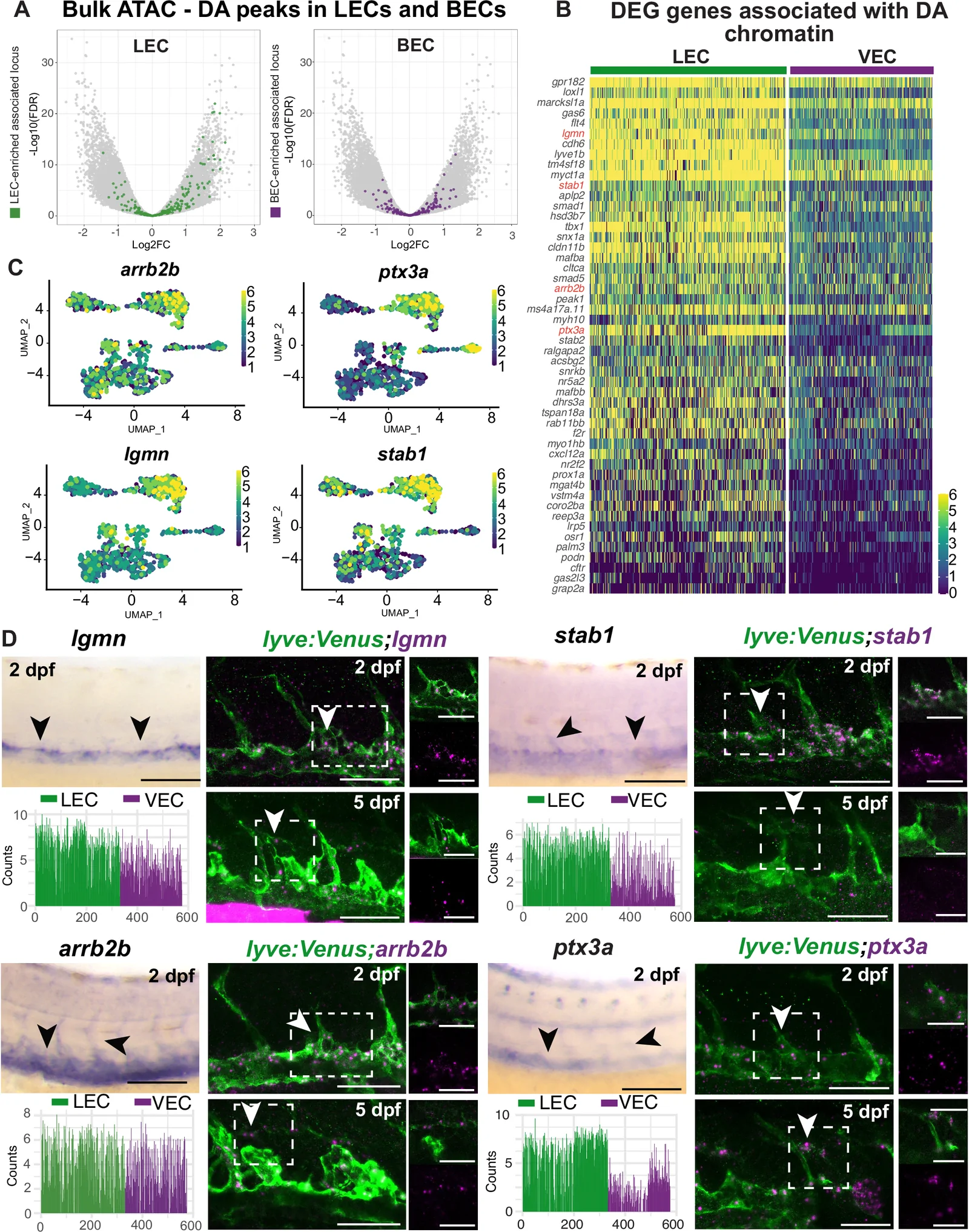
A combination of ATAC-sequencing and scRNA-sequencing data reveals novel LEC factors. (**A**) Volcano plots of differentially accessible (DA) peaks in LECs and BECs in zebrafish. Coloured dots indicate the differentially expressed (DE) status of the nearest gene in scRNA-seq data at 5 dpf. (**B**) Heatmap of expression of genes associated with the top DA ATAC-seq peaks at 5 dpf in the main LEC and VEC clusters (Fig. 2A). Cells are ordered by clusters. Colour indicates log-normalised read counts. (**C**) Expression of *lgmn*, *stab1, arrb2b* and *ptx3a* at 5 dpf, selected for in vivo validation. Colour scale indicates log-normalised expression. (**D**) Standard (*N* = 45) and fluorescent (*N* = 45) in situ hybridisation showing endothelial and lymphatic expression of genes in (**C**) in the trunk at 2 dpf and 5 dpf. Cell counts for LEC and VEC clusters at 5 dpf are shown. Arrows: endothelial expression at 2 dpf and lymphatic expression at 5 dpf. Boxed area: magnified views. Scale bars: 50 µm. In situs were performed in three technical repeats. Source data are available online for this figure.

To further investigate the relation between chromatin accessibility and transcriptional regulation, we checked if an open chromatin signature in the ATAC-sequencing dataset correlated with a tissue-specific expression enrichment of the closest gene locus. We found that, for both LECs and BECs, chromatin accessibility and gene expression level are independent from each other (Fig. 3A; Dataset EV8). Within the top 50 differentially expressed genes (DEG) in LECs and BECs, we found an even split of promoters with increased and decreased accessibility in the same tissue (Fig. EV3F; Dataset EV10). This indicates that a change in chromatin accessibility alone is not a reliable indicator of altered gene activity.

We therefore investigated whether regions of open chromatin are preferentially associated with the previously identified differential TADs. We found that LEC DA were more accessible in differential TADs (*p* = 0.04, Fisher's exact test). We further investigated whether ATAC peaks preferentially accessible in LEC and associated with differential TADs can be linked to altered gene expression using a permutation test (*n* = 5000) (Fig. EV3G). We found that while no positive association could be found between BEC DA regions and differential TADs, regardless of gene expression, LEC DA regions annotated to LEC DE genes show a significant association with differential TADs. Therefore, while generally there is no strong association between differential TAD boundaries and DE genes (Fig. EV2G), LEC-specific genes tend to be associated with differential TAD boundaries, suggesting that changes in TAD structure may affect chromatin accessibility and gene expression associated with LEC identity.

To identify genes associated with both peaks of differentially accessible chromatin and enriched expression in LECs, we intersected the ATAC-sequencing and scRNA-sequencing datasets. To do so, we selected the top LEC-specific differentially accessible (DA) regions by average logarithmic fold change (log2FC), and annotated them to the name of the closest gene locus. We then imposed the condition that the genes must be DEG in LECs with log2FC ≥0.3 and adjusted *p* value <0.1. We identified 90 genes at 5 dpf and 107 genes at 7 dpf (Figs. 3B and S1A; Datasets EV11 and 12) satisfying these criteria. We plotted the top 50 genes by logFC and with an average signal log2CPM ≥2 in ATAC-sequencing, ordered by the fraction of cells expressing them in scRNA-sequencing (Fig. 3B and S1A; Datasets EV11 and 12). We selected four genes for further characterisation: *arrb2b*, *ptx3a*, *lgmn* and *stab1*. Out of these, *stab1* and *ptx3a* were selected due to their previously described expression in the lymphatic endothelium in other models (Kzhyshkowska, 2006; Petkova et al, 2023; Xiang et al, 2020). *arrb2b* and *lgmn* do not have a well-described role in the vasculature, and were therefore selected to test the potential for the discovery of new endothelial factors. At 5 dpf, their expression is enriched in LECs (Fig. 3C), and maintained at 7 dpf (Fig. S1B). To validate the expression of these genes in the zebrafish lymphatic endothelium, we have carried out chromogenic and fluorescent in situ hybridisation (Fig. 3D), and found that all four genes are expressed in the venous sprouts at 2 dpf and in the lymphatic endothelium at 5 dpf in both trunk and face (Figs. 3D and S1C). Controls with sense probes showed no expression (Fig. S1D). Hence, by integrating the ATAC-sequencing and scRNA-sequencing datasets, we identified genes presenting both differentially accessible chromatin and enriched expression in LECs, including previously unidentified candidates for lymphatic development, underscoring the robustness and the discovery potential of our datasets.

### LEC chromatin landscape reveals accessible enhancers in the proximity to LEC-enriched genes

**Figure 4:**
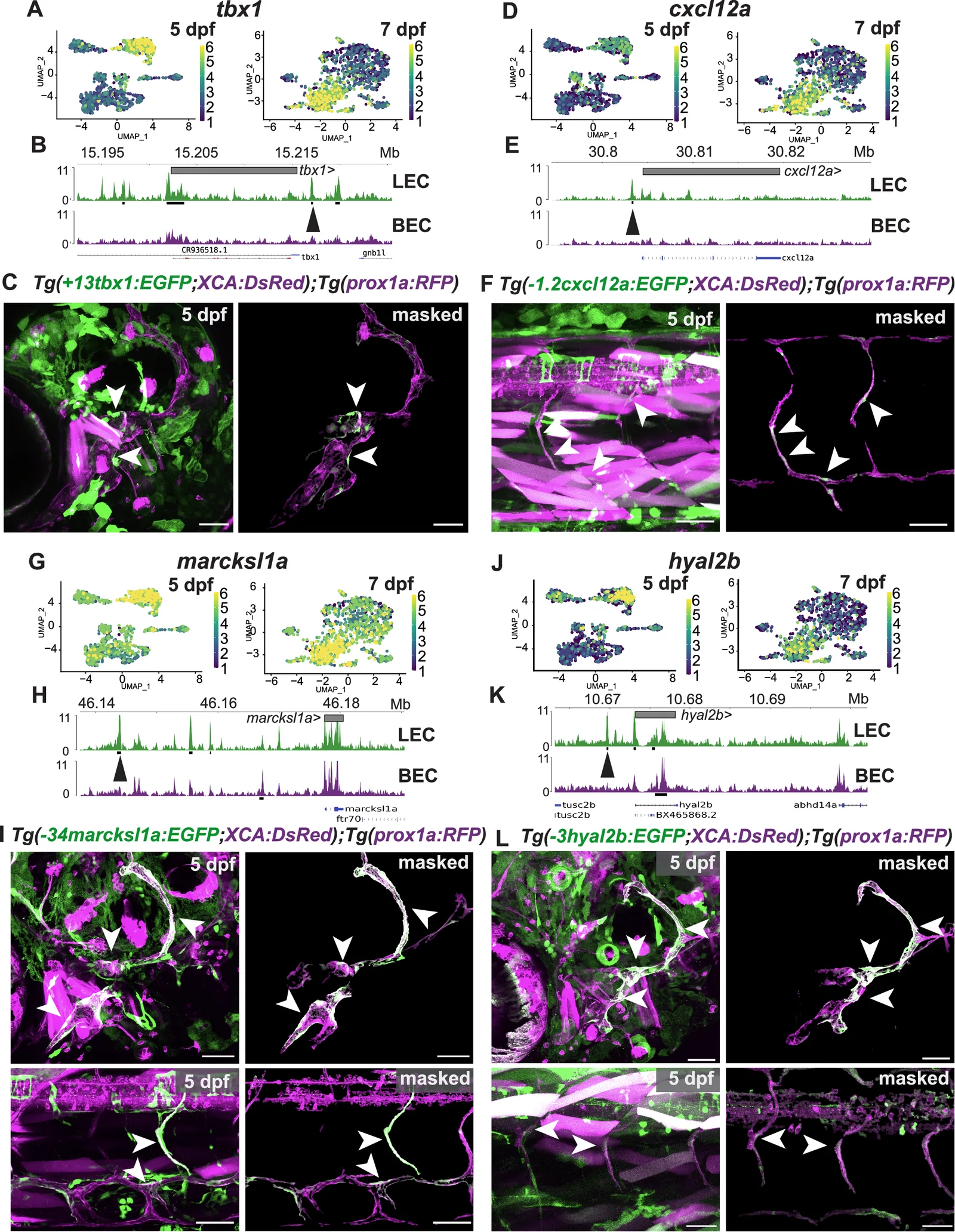
Characterisation of four novel ATAC-based LEC enhancers. (**A**–**C**) *+13tbx1* enhancer. (**D**–**F**) *-1.2cxcl12a* enhancer. (**G**–**I**) *-34marcksl1a* enhancer. (**J**–**L**) *-3hyal2b* enhancer. (**A**, **D**, **G**, **J**) LEC-enriched gene expression at 5 dpf and 7 dpf in zebrafish. Colour scale indicates normalised expression. (**B**, **E**, H, **K**) LEC and BEC chromatin accessibility at the gene locus. Black boxes: DA peaks. Arrows: identified enhancers. (**C**, **F**, **I**, **L**) Confocal and masked expression driven by the enhancers in 5 dpf zebrafish. Arrows: expression in the lymphatics. (**C**) *Tg(+13tbx1:EGFP;XCA:DsRed);Tg(prox1a:RFP)* embryo, enhancer activity in the facial lymphatics. (**F**) *Tg-1.2cxcl12a:EGFP;XCA:DsRed);Tg(prox1a:RFP)* embryo, enhancer activity in the trunk lymphatics. (**I**) *Tg(-34marcksl1a:EGFP;XCA:DsRed);Tg(prox1a:RFP)* embryo, enhancer activity in the facial and trunk lymphatics. (**L**) *Tg(-3hyal2b:EGFP;XCA:DsRed);Tg(prox1a:RFP)* embryo, enhancer activity in the facial and trunk lymphatics. Scale bars: 50 µm. Source data are available online for this figure.

Gene expression is regulated through active enhancers marked by an open chromatin conformation. To further explore the ATAC-sequencing results, we looked for potential LEC enhancers to be tested in vivo. Such candidates were defined as LEC-specific peaks of open chromatin in the non-coding regions surrounding genes enriched in the LEC clusters. Four of these putative enhancers were selected by visual inspection based on signal intensity and easy attribution to a gene (Fig. 4A,B,D,E,G,H,J,K), and tested in vivo by cloning into the ZED vector (Bessa et al, 2009) (Figs. 4 and S2B). Their activity was tested by screening the injected F0 for lymphatic expression. One of the enhancers is located close to *tbx1*, a gene with a well-described role in lymphatic development (Chen et al, 2010), while the others are located close to previously undescribed lymphatic candidates: *hyal2b, cxcl12a* and *marcksl1a*. This analysis also identified a previously described (Grimm et al, 2023) *cdh6* lymphatic enhancer (Fig. S1E,F). Moreover, our previous work (Kazenwadel et al, 2023; Panara et al, 2024) described five *prox1a* enhancers active in different compartments of the lymphatics. Out of these, four (-*84prox1a*, -*71prox1a*, -*14prox1a* and -2.*1prox1a*) could also be identified as a peak of open chromatin in LECs (Fig. S1G). The fifth lymphatic enhancer, *+15.2prox1a*, did not show a clear signature of open chromatin, in accordance with the original publication. Overall, these observations validate the potential of the dataset for revealing tissue-specific cis-regulatory sequences.

All of the novel tested enhancers showed expression in the lymphatic endothelium (Fig. 4C,F,I,L). We further confirmed the identity of the enhancers by verifying that none of the neighbouring genes showed a clear LEC enrichment (Fig. S1H).

Some of the tested enhancers, such as *+13tbx1* and *-1.2cxcl12a*, showed activity within endothelium restricted to a specific lymphatic bed, respectively, the facial or the trunk lymphatics (Fig. 4C,F). On the other hand, -*34marcksl1a* and -3*hyal2b* drove expression in both facial and trunk lymphatic vessels, with additional activity in the blood vascular beds (Fig. 4I–L). In conclusion, by using our ATAC-sequencing and scRNA-sequencing datasets, we were able to uncover a set of enhancers that drive the expression in a topologically restricted manner, confined to specific vascular beds.

### The genomic features connected by LEC- and BEC-specific chromatin loops differ

In addition to the identification of TADs, HiC data can be employed to investigate the presence of chromatin loops. These represent strong physical interaction between linearly distant genomic features, and mediate regulatory interactions. Chromatin loops were detected in two independent replicates (Fig. S3A). The loops present in both replicates within a distance of 25 kb were considered as tissue-reproducible. Tissue-reproducible loops without an overlapping or adjacent loop within 40 kb in the other tissue were considered tissue-specific. Overlapping loops and loops within 40 kb in both tissues were considered common loops.

**Figure EV4:**
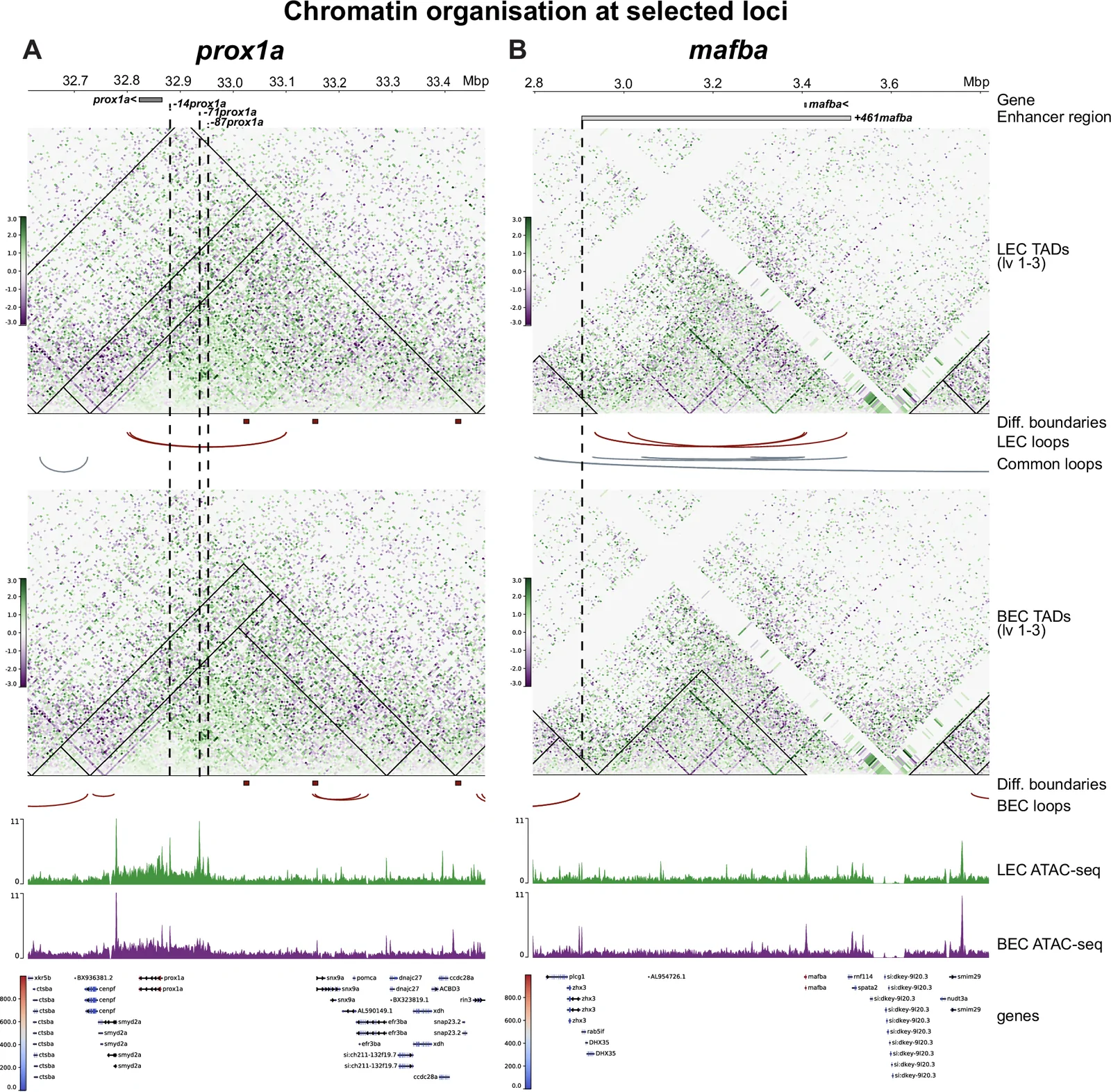
Characterisation of chromatin state at key lymphatic loci. (**A**,** B**) Chromatin organisation at the *prox1a* (**A**) and *mafba* (**B**) loci in LECs and BECs. Log2 of the HiC signal in LEC vs BEC is shown. LEC and BEC TADs of levels 1–3 are plotted in black. Differential boundaries are shown in red. The dotted line indicates the position of the putative enhancers. Tissue-specific loops plotted in red. Common loops plotted in grey. Chromatin accessibility plotted in green for LECs and purple for BECs.

By integrating these lists, we identified the presence of 7061 common loops, 2290 BEC-specific loops and 1095 LEC-specific loops (Fig. S3A; Dataset EV13). Both tissue-specific and common chromatin loops could be identified at the gene loci of known lymphatic regulators, such as *prox1a* (Fig. EV4A) and *mafba* (Fig. EV4B).

To determine if certain genomic interactions are enriched in the lymphatic endothelium, we characterised the type of features involved in loop formation. First, we identified open chromatin regions placed at the extremities of tissue-specific loops (Fig. S3B). An accessible chromatin region was assigned to the end of a loop when it fell within 10 kb of the loop matrix bin (25 kb). Quantification of the interactions detected in LEC- and BEC-specific loops determined a total of 1996 interactions specific to LECs and 3462 to BECs (Fig. S3B; Dataset EV14).

We then characterised the differences in interaction types between LECs and BECs by considering the genomic features assigned to the accessible regions at the end of loops. First, to exclude the possibility that the differences in interactions were mirroring underlying differences in the distribution of open chromatin in different genomic features, we used a chi-square test for goodness of fit between observed and expected frequencies. We confirmed that the distribution of genomic features was different from the one expected by randomly picking two open genomic features in the genome (LEC: *p* < 0.0001, BEC: *p* < 0.0001) (Fig. S3C).

We then used a chi-square test to compare the frequency of different interaction types in LECs and BECs. The analysis confirmed that the distributions were significantly different (*p* < 0.0001), suggesting that not only the location but the type of interactions varies between endothelial cell types. Specifically, the map of the residuals revealed an enrichment of promoter–promoter and promoter-distal interactions in BEC-specific loops, and an enrichment of promoter-intron, promoter-exon, exon-intron, exon-distal, intron-distal and distal-distal interactions in LEC-specific loops (Fig. S3D). These data revealed distinct differences in the underlying features of loop formation between LECs and BECs, which may shape their divergent developmental trajectories.

### LEC chromatin architecture reveals candidate HiC-based enhancers interacting with LEC-enriched genes

**Figure 5:**
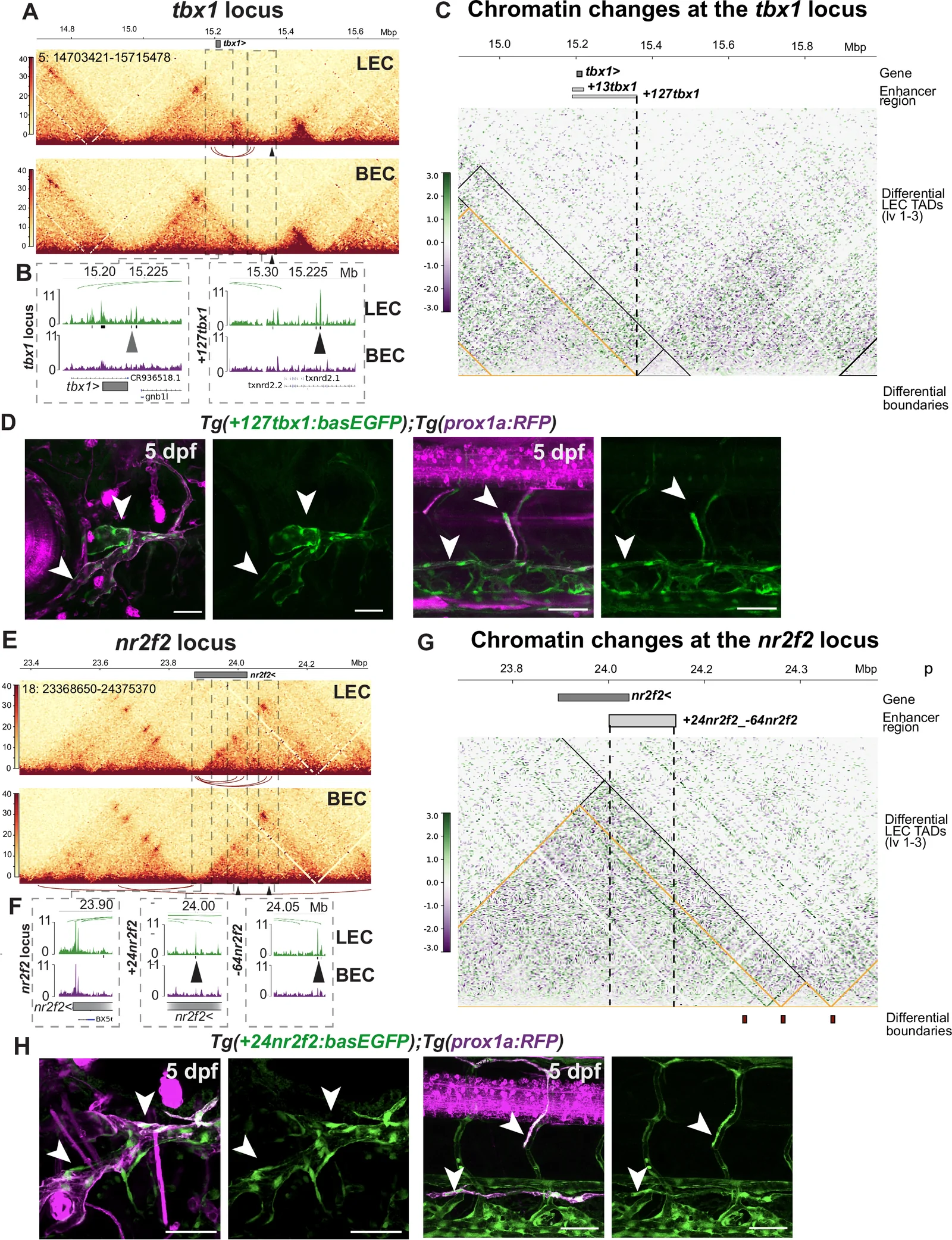
Characterisations of novel HiC-based LEC enhancers. (**A**) LECs and BECs HiC maps showing tissue-specific interaction around *tbx1* in zebrafish. Tissue-specific loops shown in red. Arrow: *+127tbx1*. Dotted box: regions in (**B**). (**B**) Chromatin accessibility and tissue-specific chromatin loops at the *tbx1* locus and the *+127tbx1* enhancer in LECs and BECs. Black boxes: DA peaks. Black arrow: *+127tbx1*. Grey arrow: *+13tbx1*. (**C**) TAD organisation surrounding *+127tbx1*. Log2 of the HiC signal in LEC vs BEC is shown. Non-differential LEC TADs of level 1–3 are plotted in black and differential LEC TADs in orange. Differential boundaries are shown in red. The dotted line indicates the position of the *+127tbx1* enhancer. (**D**) *Tg(+127tbx1:basEGFP);Tg(prox1a:RFP)* confocal and masked expression, showing activity in the endothelium, including facial and trunk lymphatics at 5 dpf (arrows). (**E**) LECs and BECs HiC maps showing tissue-specific interaction around *nr2f2*. Tissue-specific loops shown in red. Arrow: identified enhancers. Dotted box: regions in (**F**). (**F**) Chromatin accessibility and tissue-specific chromatin loops at the distal part of the *nr2f2* locus, +*24nr2f2*, and -*64nr2f2* in LECs and BECs. Black boxes: DA peaks. Arrows: identified enhancers. (**G**) TAD organisation surrounding the putative *nr2f2* enhancers. Log2 of the HiC signal in LEC vs BEC is shown. Non-differential LEC TADs of level 1–3 are plotted in black and differential LEC TADs in orange. Differential boundaries are shown in red. The dotted line indicates the position of the putative enhancers. (**H**) *Tg(+24nr2f2:basEGFP);Tg(prox1a:RFP)* confocal and masked expression, showing activity in endothelium, including the facial and trunk lymphatics at 5 dpf (arrows). Scale bars: 50 µm. Source data are available online for this figure.

**Figure 6:**
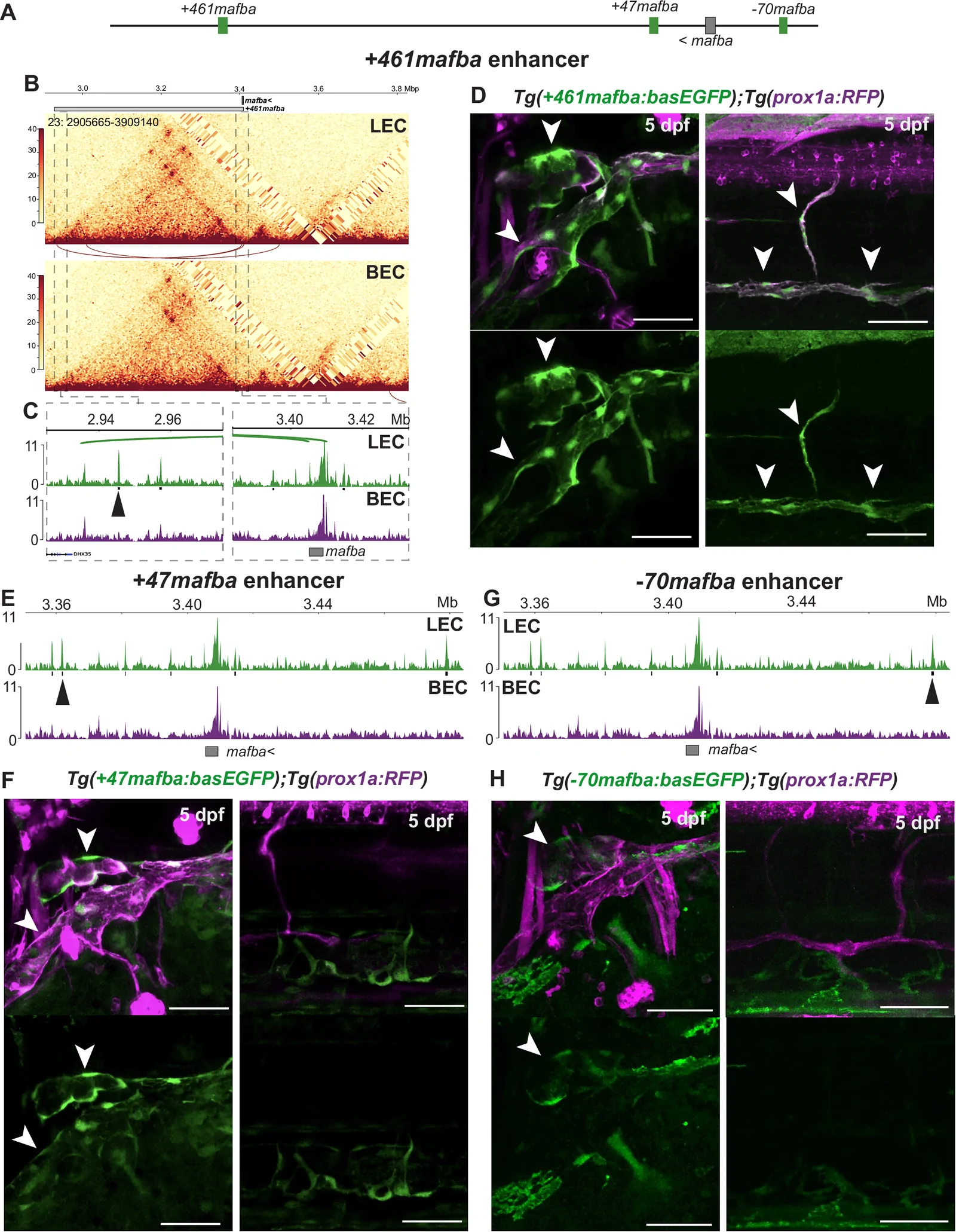
A set of enhancers of *mafba* drives expression in a cell type-specific manner. (**A**) Schematics of the identified *mafba* enhancers at the zebrafish *mafba* locus. (**B**) HiC maps in LECs and BECs showing tissue-specific interaction around *mafba*. Tissue-specific loops plotted in red. (**C**) Chromatin accessibility and tissue-specific loops at the *mafba* locus and *+461mafba* in LECs and BECs. Black boxes: DA peaks. Arrows: *+461mafba*. (**D**) *Tg(+461mafba:basEGFP);Tg(prox1a:RFP)* embryo, showing activity in the facial and trunk lymphatics (arrows). (**E**) Chromatin accessibility at the *mafba* locus in LECs and BECs. Black boxes: DA peaks. Arrow: *+47mafba*. (**F**) *Tg(+47mafba:basEGFP);Tg(prox1a:RFP)* embryo at 5 dpf, showing activity in the facial lymphatics (arrows). (**G**) Chromatin accessibility at the *mafba* locus in LECs and BECs. Black boxes: DA peaks. Arrow: *-70mafba*. (**H**) *Tg(-70mafba:basEGFP);Tg(prox1a:RFP)* embryo at 5 dpf, showing activity in the facial lymphatics (arrows). Scale bars: 50 µm. Source data are available online for this figure.

**Figure EV5:**
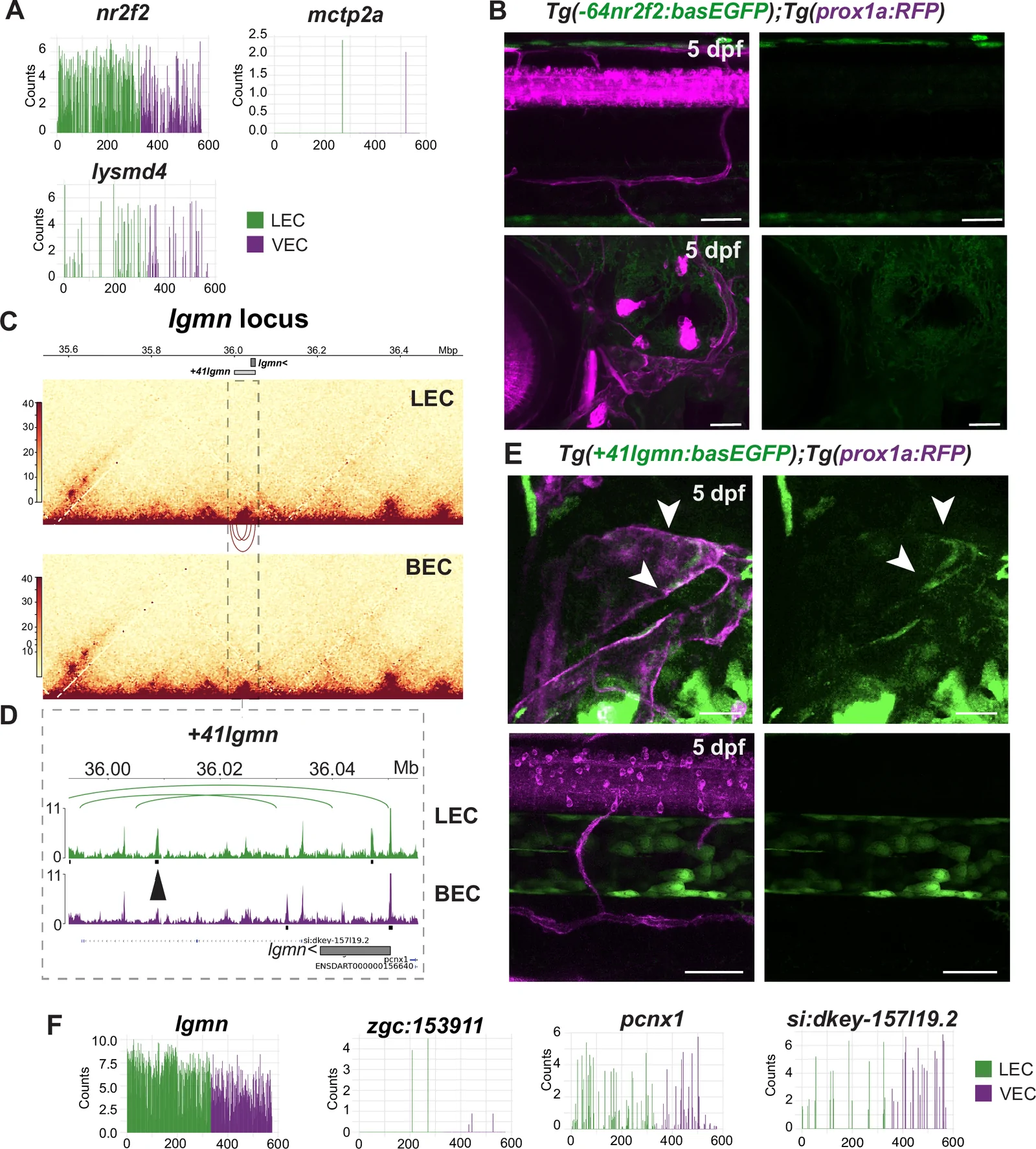
Additional characterisation of novel HiC-based LEC enhancers. (**A**) Cell counts of gene loci surrounding *nr2f2* in LEC and VEC clusters at 5 dpf, showing enriched expression at selected loci. (**B**) *Tg(+62nr2f2:basEGFP);Tg(prox1a:RFP)* embryo, showing no enhancer activity in lymphatics at 5 dpf. (**C**) HiC maps in LECs and BECs showing tissue-specific interactions at the *lgmn locus*. Dotted box: regions in (**E**). (**D**) Chromatin accessibility and tissue-specific loops at the *lgmn* locus and +*41lgmn* in LECs and BECs. Black boxes: DA peaks. Arrow: +*41lgmn*. (**E**) *Tg(+41lgmn:basEGFP);Tg(prox1a:RFP)* confocal and masked expression, showing activity in the facial lymphatics at 5 dpf (arrows). (**F**) Cell counts of gene loci around *lgmn* in LEC and VEC clusters at 5 dpf, showing enriched expression at selected loci. Scale bars: 50 µm. Source data are available online for this figure.

Next, we wanted to functionally characterise the tissue-specific chromatin loops. HiC, ATAC- and scRNA-sequencing data were combined to identify LEC-specific loop-mediated enhancer-promoter interaction. We first defined candidate enhancer sequences as differentially accessible intronic or distal regions of non-coding DNA which interact with the open promoter of an LEC-accessible gene through a LEC-specific loop (Fig. S3E), following the same rationale as previously described to assign accessible regions to a loop end. The analysis identified 95 gene loci involved in this kind of interactions. To identify genes with which HiC-based enhancer interaction coincided with increased expression in the lymphatic endothelium, we intersected the candidate list with genes significantly enriched in LECs in the scRNA-sequencing dataset. This analysis identified 7 genes at 5 dpf and 6 genes at 7 dpf (Fig. S3E). Out of these, *tbx1* (Fig. 5A,B), *mafba* (Fig. 6B) and *nr2f2* (Fig. 5E,F) showed a clear interaction between the promoter and a putative distal regulatory element, and have previously been associated with lymphatic development (Arnold et al, 2022; Chen et al, 2010; Koltowska et al, 2015a; Srinivasan et al, 2010), while *lgmn* (Figs. EV5C–F and S2A) has not been previously associated with lymphatic vessels. We then asked whether these long-range interactions and changes in chromatin accessibility are also accompanied by changes in TADs between LECs and BECs, and we found that the loops were associated with TAD changes at the *tbx1* and *nr2f2* loci (Fig. 5C,G). This change in organisation results in the *+127tbx1* putative enhancer falling inside a differential LEC TAD, very close to its border (Fig. 5C). In the *nr2f2* locus, we observed the presence of a large LEC-specific TAD, encompassing both putative enhancers, as well as several differential boundaries in the surrounding region (Fig. 5G).

To validate these HiC-based enhancers in vivo, we generated constructs using a vector with an *e1b TATA* minimal promoter (Villefranc et al, 2007), as we have previously confirmed that the empty plasmid does not drive any vascular expression (Panara et al, 2024). An analysis of the stable line for *+127tbx1* showed expression in the facial and trunk lymphatic vessels, as well as the posterior cardinal vein (Figs. 5D and S2A). As this enhancer sits 127 kb downstream of *tbx1*, we checked the expression of the surrounding genes in endothelial cells using the scRNA-sequencing dataset, finding minimal expression of *txtrd2.1, txtrd2.2* and *pebp1* in both LECs and VECs, further supporting the specificity of the enhancer-driven expression (Fig. S3F).

For *nr2f2*, we identified two enhancers: one located 24 kb downstream and one 64 kb upstream from the transcription start site (Fig. 5E,F). The *+24nr2f2* enhancer drove expression in facial lymphatics, as well as in arterial and venous vasculature (Figs. 5H and S2A). The *-64nr2f2* enhancer showed no expression in the endothelium (Figs. EV5B and S2A). The surrounding genes (*lysmd4* and *nctp2a*) were not expressed in the LEC and VEC clusters in the 5 dpf scRNA-sequencing dataset (Fig. EV5A).

Noticeably, for both +24*nr2f2* and +127*tbx1*, reporter activity was detected in the endothelial population, which are marked by low expression of the corresponding gene: for *tbx1*, in the veins (Fig. 4A) and for *nr2f2*, in the artery (DanioCell, Sur et al, 2023). This expanded domain of activity likely reflects the presence of additional regulatory interactions at the endogenous gene locus, which inhibit the expression in these populations.

For *lgmn* we have identified a HiC-based enhancer 41 kb downstream of the gene locus (Fig. EV5C,D) driving expression only in the facial lymphatics but not in the trunk (Figs. EV5E, and S2A). The surrounding genes (*zgc:153911, pcnx1, si:dkey-157l19.2*) did not show expression in the LEC and VEC transcriptomic data at 5 dpf (Fig. EV5F).

By leveraging chromatin looping, we identified enhancers that drive tissue-specific expression in the endothelium, revealing a previously unappreciated multifaceted nature of transcriptional regulation of lymphatic-enriched genes. Notably, two of these genes are located within regions subject to differential TAD formation in LECs and BECs, implicating different chromatin conformation as a mechanism for regulating cell lineage-restricted expression. Together, these data reveal a complex regulatory landscape in which lymphatic-enriched gene expression is orchestrated by chromatin organisation through local enhancers, long-range interactions, or a combination of both.

### A set of enhancers of mafba drives expression in a cell type-specific manner

To determine how the intricate regulatory landscape is linked to the regulation of key transcription factors for specific cellular processes, we focused on *mafba*. *mafba* expression has been reported in both LECs and VECs by in situ hybridisation and is necessary for LEC migration (Koltowska et al, 2015b). The scRNA-sequencing revealed that *mafba* is differentially expressed between LECs and VECs, with higher expression in LECs at both 5 and 7 dpf (Fig. EV2D,F). To understand how this gene regulation is achieved in LECs and VECs, we have focused on identifying and characterising some of its vascular enhancers. Although no TAD structural changes were detected in the *mafba* locus, we have selected three candidate sequences, *+461mafba*, *+47mafba* and *-70mafba* (Fig. 6A–C,E,G), based on a combination of ATAC-sequencing and HiC. Specifically, *+461mafba* was picked based on the presence of a LEC-specific chromatin loop connecting it to the *mafba* gene, while *+47mafba* was selected at random among the numerous LEC-specific peaks of chromatin accessibility found in the large gene desert downstream of the *mafba* locus. Finally, *-70mafba* was selected in order to test if the shorter region upstream of *mafba* also contained vascular enhancers. Tested in vivo, all three candidates can drive the expression in the facial lymphatic vessels, with *-70mafba* being more restricted to the facial collecting lymphatic vessel (Figs. 6D,F,H and S2A). Expression in the trunk lymphatic vessels was observed for *+461mafba* (Figs. 6D and S2A), whereas blood endothelial expression was observed for *+47mafba* and *-70mafba* (Figs. 6F,H and S2A). Interestingly, the *+461mafba* enhancer falls at the end of a LEC-specific loop, supporting direct chromatin interaction with the *mafba* promoter (Fig. 6B,C). However, this element is placed 461 kilobases away from *mafba* and close to other genes. Therefore, we checked the expression of these gene loci and found only a small percentage of LECs expressing *rnf114*, *DHX35, rebi5if, zhx3 and plcg1*. When compared with the robust expression of *mafba* (Fig. S3G), this provides further evidence of the enhancer’s role as a regulator of *mafba* in LECs.

Together, these data highlight the importance of considering chromatin loops in the reconstruction of gene regulation, as spatially distant enhancers may work in concert to fine-tune transcriptional control. The complexity of *mafba* regulation, with multiple enhancers being capable of driving the expression in different vascular beds, suggests that coordinated enhancer activity may be critical for modulating lineage-specific expression. In addition, the presence of several chromatin loops, both common and tissue-specific, surrounding the *mafba* locus (Fig. EV4B) further strengthens the idea that *mafba* regulation in the vasculature depends on complex mechanisms, involving several layers of chromatin organisation. In this context, it must be noted that several *mafba* candidate vascular enhancers remain untested, and likely contribute to this regulatory mechanism. This complex regulation of *mafba, a* key factor for LEC migration, further emphasises the intricate link between enhancer activity and cellular functions.

### Specific predicted TF binding sites are enriched in LECs and BECs at 5 and 7 dpf

**Figure 7:**
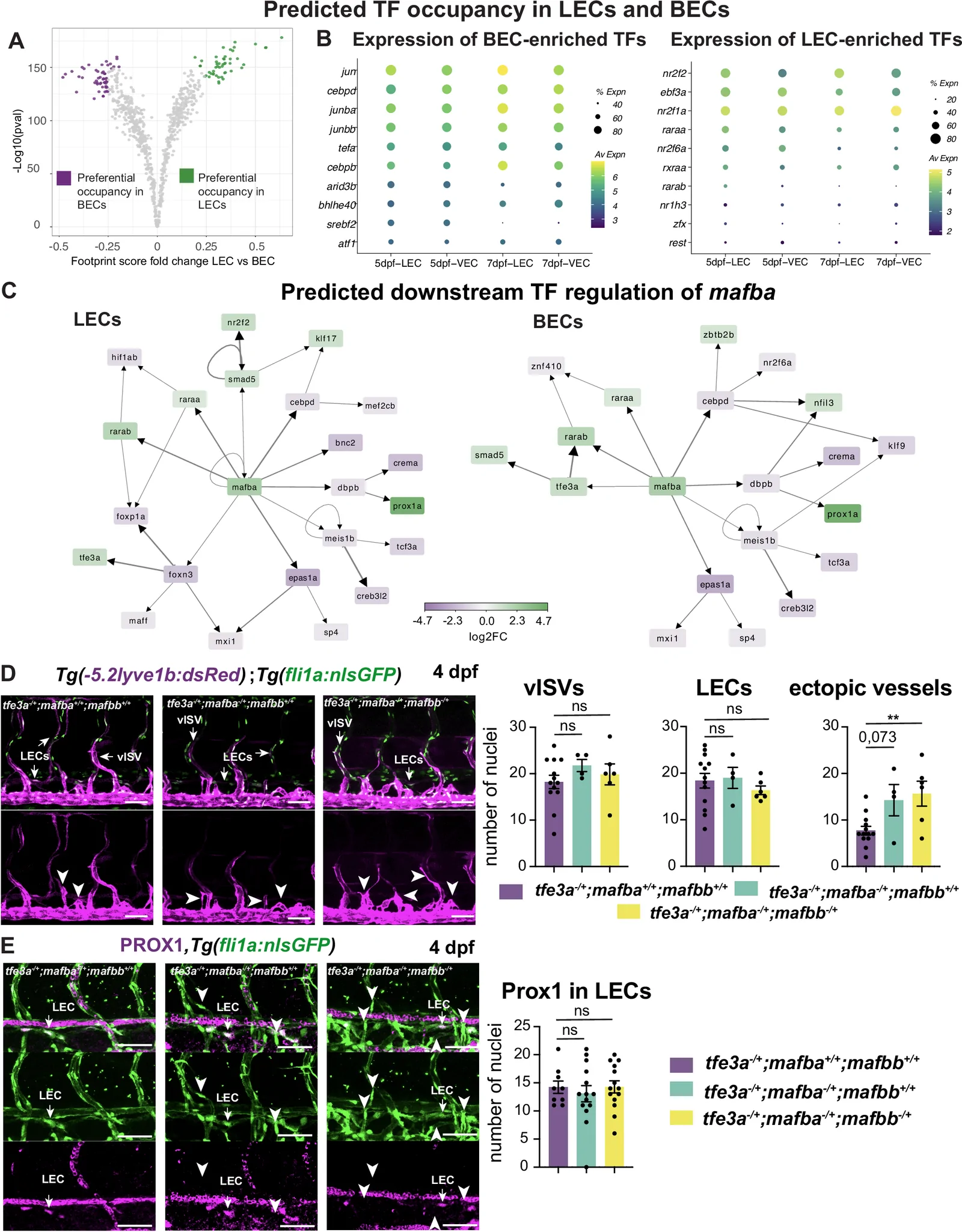
Characterisation of the *mafba* downstream regulatory network in LECs and BECs. (**A**) Comparison of TF footprints in LECs vs. BECs. The top/bottom 5% by score difference is highlighted. (**B**) Dot plots showing expression of BEC- (left) and LEC-enriched (right) TFs at 5 dpf and 7 dpf. Dot size indicates % of cells expressing the transcript (% Expn). Viridis scale illustrates average normalised expression (Av Expn) within a cluster. (**C**) Two-layered TF networks downstream of *mafba* in LECs and BEcs, predicted by TOBIAS. Green: expression enriched in LECs at 5 dpf. Purple: expression enriched in BECs at 5 dpf. (**D**) Ectopic vessel formation in triple-heterozygous *tfe3a-/+;mafba-/+;mafbb-/+* zebrafish. Left: Confocal images of *Tg(-5.2lyve1b:dsRed);Tg(fli1a:nlsGFP)* trunk vasculature at 4 dpf in *tfe3a−/+* (*N* = 13), *tfe3a−/+;mafba−/+* (*N* = 4) and *tfe3a−/+;mafba−/+;mafbb−/+* (*N* = 6) embryos. Arrows: vessel identity. LECs: lymphatic endothelial cells, vISV: venous intersegmental vessels. Arrowheads: ectopic vessels. Right: Quantification of nuclei number in vISV, LECs and ectopic vessels: one-way ANOVA, mean ± SEM, ns=no significance, **p value 0,0097. (**E**) Quantification of Prox1-expressing LECs at 4 dpf. Left: immunostaining against Prox1 in the trunk of *Tg(fli1a:nlsGFP) tfe3a−/+* (*N* = 9), *tfe3a−/+;mafba−/+* (*N* = 13) and *tfe3a−/+;mafba−/+;mafbb−/+* (*N* = 14) embryos. Arrows: Lymphatic vessels. Arrowheads: LECs nuclei. Right: Quantification of PROX1- and GFP-positive nuclei number in LECs. one-way ANOVA, mean ± SEM, ns no significance. Scale bars: 50 µm.

In our analysis so far, we have focused on the role of chromatin accessibility on cis-regulatory elements activity. However, an equally important aspect of gene regulation is mediated by the transcription factors, which bind to such elements and promote the interaction with the transcriptional complex. In order to integrate these two aspects, we leveraged our identification of tissue-specific transcriptional signatures and chromatin organisation to explore the regulatory networks that establish and maintain the gene expression programs of LECs and BECs. To do so, we first identified the transcription factor binding sites, which are predicted to be bound in the accessible chromatin regions. We analysed the TF footprints in promoter ATAC peaks to predict the TF binding sites preferentially occupied in each cell type using the TOBIAS tool (Fig. 7A; Dataset EV15). Only promoters were considered for this analysis as their attribution to a specific gene is more robust than for enhancers, which can often regulate distant loci. A proportion of the genes associated with these promoters showed upregulated expression in LECs or BECs (Fig. 7A; Dataset EV15). We then asked whether the TFs whose motifs were preferentially occupied in LECs and BECs were also enriched in their corresponding cell type in the scRNA-sequencing dataset. However, with the exception of *nr2f2* (which is both preferentially occupied and enriched in LECs), all of the TFs whose motifs were preferentially occupied in one tissue showed comparable expression levels in LECs and VECs (Fig. 7B; Datasets EV16 and 17), suggesting that TF RNA levels alone do not necessarily reflect their biological relevance.

To further harness the potential of our dataset, we used the TF footprinting results to investigate the regulatory networks downstream of Mafba, aiming to determine the molecular mechanism by which it regulates lymphatic vessel formation. We identified promoters containing predicted occupied Mafba binding sites and compared them between LECs and BECs. We identified 1364 predicted sites of occupancy of the Mafba motif at gene promoters in both LECs and BECs, while in 417 sites the motif is predicted to be occupied only in BECs and in 192 sites only in LECs (Fig. S4A; Dataset EV18). Out of the common targets, 17 genes showed enriched expression in LECs, while 11 were enriched in the VECs at 5 dpf (scRNA-sequencing data with a cutoff average log2FC of 0.3, Fig. S4B; Dataset EV19). Within the promoters predicted to have sites exclusively occupied in LECs, 12 genes showed enriched expression in LECs, and seven genes showed enriched expression in the VECs at 5 dpf. On the other hand, in the promoters predicted to have sites exclusively occupied in BECs, 0 genes showed enriched expression in LECs and three showed enriched expression in VECs at 5 dpf (Fig. S4B; Dataset EV19). This suggests a complex setup of gene transcription regulatory network, with the likely cooperation of multiple TFs to drive LEC and VEC gene expression and hints at the potential of Mafba to act as both an activator and repressor.

To fully understand the complexity of Mafba downstream regulation, we built a TF-based two-layered transcriptional network for LECs and BECs (Fig. 7C). To focus on the differences between LECs and BECs, we included only TFs which were differentially expressed at 5 dpf. In addition to the consensus Mafb motifs from Jaspar, we also considered an endothelial-specific binding motif for *mafba* previously identified in a BEC ChIP-sequencing dataset (Jeong et al, 2017). The number of occupied motif instances in LECs and BECs was lower for the endothelial Mafba motif compared to the Jaspar consensus motifs, with few genes showing enriched expression in LECs and VECs (Fig. S4C,D; Datasets EV20 and 21). As a result, the network built on the endothelial motif is composed of only a few genes (Fig. S4E). This limited predictive ability suggests that this motif might be restricted to BECs. We therefore considered the predictions based on the common motifs going forward, as we were interested in a wider variety of endothelial cells. Noticeably, in both networks we found genes enriched in both LECs or VECs, further supporting the idea that chromatin accessibility does not necessarily indicate active expression (Fig. 7C). These networks contain known genes involved in vascular and lymphatic development, including members of the retinoic acid signalling family (Bowles et al, 2014), the BMP signalling pathway (Dunworth et al, 2014), and the TFs Prox1 (Koltowska et al, 2015a; Wigle et al, 1999) and Nr2f2 (Srinivasan et al, 2010) (Fig. 7C).

We have identified the transcriptional signatures characteristic of LECs and BECs, and mapped the downstream regulatory networks of Mafba, uncovering a complex, context-dependent regulatory landscape. The disconnect between lineage-specific gene expression and predicted TF activity highlights the necessity of integrating analysis on multiple layers of gene regulation. Altogether, our multi-omics approach reveals the intricacies of transcriptional control during LEC differentiation and emphasises the critical importance of in vivo validation to decode cell type-specific regulatory programmes.

### Mafba and Tfe3a cooperate to limit ectopic vessel formation

To validate the biological relevance of the identified Mafba transcriptional networks, we focused on the *tfe3* gene (Fig. 7C). This gene has not been previously associated with lymphatic development in zebrafish, but has been shown in mice to repress lymphatic fate (Tai-Nagara et al, 2020). Tfe3a also cooperates with Mafb to coordinate macrophage differentiation (Zanocco-Marani et al, 2009). *tfe3a* was present in both the LEC and BEC Mafb transcriptional networks; thus, we wanted to investigate its role in the regulation of lymphatic vessel formation. To test if *tfe3a* and *mafba* genetically interact during lymphatic vessel development, we have generated *tfe3a* mutants by introducing a 13 bp insertion in exon 5, resulting in a premature stop upstream of the Helix-loop-helix DNA-binding domain 1 (Fig. S4F). The *tfe3a* mutants grow to adulthood and show no apparent phenotypes. As *mafba* and *mafbb* compensate for each other, and the double-mutants do not develop lymphatic vessels (Arnold et al, 2022) we decided to test the interaction in triple-heterozygous embryos (Fig. 7D). When comparing *tfe3a* heterozygotes with *tfe3a, mafba* double-heterozygotes or *tfe3a, mafba, mafbb* triple-heterozygotes we noticed no apparent defects in lymphatic or venous vessel formation (Fig. 7D). Lymphatic vessels differentiate correctly, and show Prox1 expression as well as regular morphology (Fig. 7E). Similarly, the venous vessels appear correctly formed and functional (Fig. 7D). However, in *tfe3a, mafba, mafbb* triple-heterozygous embryos we found a significant increase in ectopic vessels sprouting out of the PCV. Such vessels do not connect to the intersegmental arteries and remain un-luminised (Fig. 7D). The ectopic vessels did not acquire neither lymphatic identity nor venous functionality. These findings demonstrate that *tfe3a* genetically interacts with *mafba* and *mafbb* to limit ectopic vessel formation and ensure correct vascular differentiation, validating the predictive power of our regulatory networks in uncovering functional genetic interactions shaping vessel formation.

## Discussion

Since the debut of HiC as a powerful tool to investigate chromatin 3D conformation, the consensus has been that TADs are stable structures across different cell types, while the loops they contain shift in a tissue-specific manner to modulate transcriptional activity (Pongubala and Murre, 2021). However, this consensus has recently come under scrutiny, as examples of TADs changing during development (Boya et al, 2017; Marti-Marimon et al, 2021; Narendra et al, 2016; Bonev et al, 2017) or between similar cell lineages (Cresswell and Dozmorov, 2020) emerged. In this work, we have identified a large number of TADs showing differences between two types of endothelial cells, despite LECs and BECs being overall very closely related cell types. These differences indicate that TADs are likely a much more complex feature than initially believed, featuring a nested structure and dynamic changes in their boundaries, and likely play a large role in regulating developmental processes.

Differences in chromatin organisation were also identified at the level of chromatin loops, and the accessible regions they connect. Interestingly, we found that not only the location of loops varies between LECs and BECs, but also that the classes of genomic elements interacting through these loops differ between these two populations. Although the standard model for *cis*-regulation sees the contact of a promoter with an enhancer, different interactions, such as promoter–promoter or enhancer–enhancer, have been reported (Li et al, 2012). Promoter–promoter interactions, which in our data are enriched in BECs, have been associated with negative gene regulation, in which one promoter works as a silencer for the other (Wei et al, 2022), while interactions among enhancer elements, enriched in LECs, have a role in driving and coordinating the expression of lineage-specific genes (Madsen et al, 2020). On the other hand, promoter-distal enhancer interactions are more prominent in BECs. Work in *Drosophila* has identified that such interactions are more prominent in differentiated cells (Pollex et al, 2024). As BECs differentiate prior to LECs, we speculate that these differential interactions are an indicator of the differentiated status of these cell types. Overall, these data provide a first look into a more complex level of the regulatory mechanisms active on a genomic level in separating the BEC and LEC lineages.

Chromatin accessibility has long been used as a standard for predicting positive gene regulation (Klemm et al, 2019). However, in our data, we did not observe a direct connection between chromatin accessibility and gene activity. Instead, open chromatin is associated with both over- and under-expressed genes in LECs vs VECs. On the other hand, the combination of gene expression data and tissue-specific chromatin accessibility peaks proved a reliable tool to identify vascular enhancers. In general, the predictive power of chromatin accessibility and other markers of enhancer activity in the endothelial population has been a point of discussion, with some studies finding them a solid tool (Sissaoui et al, 2020), and others showing significant discrepancies between the in silico prediction and the activity of tested elements (Nornes et al, 2025). In our case, the high rate of success for enhancer identification based on chromatin accessibility alone was likely helped by the relatively short range of the tested sequences, all under 70 kb, and the high selectivity for strongly differential peaks. Overall, this observation confirms that while a link undoubtedly exists between chromatin accessibility and gene activity, the exact relation at the individual loci level is often much more complex.

LECs arise from multiple developmental origins, with progenitors specified from VECs (Küchler et al, 2006; Sabin, 1902; Srinivasan et al, 2007; Wigle et al, 1999; Yaniv et al, 2006) mesenchymal lineage including paraxial mesoderm-derived angioblast (Huntington and McClure, 1910; Lupu et al, 2025), hemogenic endothelium (Stanczuk et al, 2015) or other endothelial sources, such as endothelial angioblast (Eng et al, 2019; Martinez-Corral et al, 2015; Nicenboim et al, 2015; Pichol-Thievend et al, 2018). To differentiate into mature LECs and establish a stable lymphatic identity, these progenitors must coordinate and unify their gene expression programs. This process requires lineage-specific chromatin organisation and precise enhancer-promoter interactions. Previous work in mice and zebrafish has begun to map chromatin accessibility during LECs progenitor specification, mostly focusing on *Prox1* gene regulation and its downstream effectors, and has identified transcription factor signatures marking the lymphatic progenitor pools (Grimm et al, 2023; Kazenwadel et al, 2023; Lupu et al, 2025; Panara et al, 2024). However, to gain a holistic understanding of lymphatic differentiation, gene regulation must be studied at multiple levels. Our goal was to characterise the complexity of gene regulation in LECs, and to define how it differs from that in BECs, a relatively similar cell population. By integrating HiC, bulk ATAC-sequencing and scRNA-sequencing, we uncovered a highly complex regulatory system. Both short- and long-range enhancers are key in driving lymphatic-specific expression of several genes. Moreover, dynamic changes in TADs boundaries selectively isolate or join regulatory elements in a regulatory hub with gene promoters, thereby contributing to the establishment of the LEC molecular identity. These findings lay the foundation for future studies investigating how chromatin architecture is reorganised during the specification of LEC progenitors, regardless of their cellular origin.

In conclusion, our study provides the first description of  3D chromatin organisation in zebrafish LECs and BECs, and validates its connection to differential gene expression in vivo. Our datasets provide a powerful tool to further investigate both the developmental mechanism of LEC identity acquisition, and the association of *cis-*dysregulation in lymphatic disease.

## Methods

**Table:** Reagents and tools table

Reagent/resource	Reference or source	Identifier or catalog number
**Experimental models**		
*Tg(fli1a:nEGFP)*^*y7*^ *(D. rerio)*	Lawson and Weinstein, 2002	ZDB-TGCONSTRCT-070117-152
*TgBAC(prox1a:KalTA4-4xUAS-ADV.E1b: TagRFP)*^*nim5*^ *(D. rerio)*	van Impel et al, 2014	ZDB-TGCONSTRCT-140521-2
*Tg(-5.2lyve1b:Venus)*^*uu1kk*^ *(D. rerio)*	Arnold et al, 2022	ZDB-ALT-230131-6
*Tg(-5*.*2lyve1b*: *DsRed2)*^*nz101*^*(D. rerio)*	Okuda et al, 2012	ZDB-TGCONSTRCT-120723-4
*Tg(fli1a:H2B-mCherry)*^*uq37bh*^ *(D. rerio)*	Baek et al, 2019	ZDB-ALT-191011-5
*TgBAC*(*ve-cad:ve-cadTS*)^*uq11bh*^ *(D. rerio)*	Lagendijk et al, 2017	ZDB-ALT-181106-3
*mafba*^*uq4bh*^ *(D. rerio)*	Koltowska et al, 2015b	ZDB-ALT-151231-5
*mafbb*^*uq47bh*^ *(D. rerio)*	Arnold et al, 2022	ZDB-ALT-230131-5
*Tg(+24nrf2f2:basEGFP;ACry:GFP)*^*uu18kk*^ *(D. rerio)*	This study	Not yet registered
*Tg(+47mafba:basEGFP;ACry:GFP)*^*uu19kk*^ *(D. rerio)*	This study	Not yet registered
*Tg(+127tbx1:basEGFP;ACry:GFP)*^*uu20kk*^ *(D. rerio)*	This study	Not yet registered
*Tg(+461mafba:basEGFP;ACry:GFP)*^*uu21kk*^ *(D. rerio)*	This study	Not yet registered
*Tg(-64nrf2f2:basEGFP; ACry:GFP)*^*uu22kk*^ *(D. rerio)*	This study	Not yet registered
*Tg(+41lgmn:basEGFP; ACry:GFP)*^*uu23kk*^ *(D. rerio)*	This study	Not yet registered
*Tg(-70mafba:basEGFP; ACry:GFP)*^*uu24kk*^ *(D. rerio)*	This study	Not yet registered
*tfe3a* ^*uu16kk*^	This study	Not yet registered
**Antibodies**		
Anti-DIG-AP antibody	Roche	Cat# 11093274910
Anti-digoxigenin POD	Roche	Cat# 11207733910
Chicken Anti-GFP	Abcam	Cat# ab13970
Anti-chicken 488	Jackson Immuno Research	Cat# 703-545-155
Rabbit anti-Prox1	AngioBio Co.	Cat# 11-002 P
α-rabbit IgG-HRP	Cell Signalling	Cat# 7074
**Oligonucleotides and other sequence-based reagents**		
ZED vector	(Bessa et al, 2009)	N/A
*+13tbx1:EGFP;XCA: DsRed* vector	This study	N/A
*-1.2cxcl12a:EGFP; XCA:DsRed* vector	This study	N/A
*-34marcksl1a:EGFP; XCA:DsRed* vector	This study	N/A
*-3hyal2b:EGFP; XCA:DsRed* vector	This study	N/A
*-48cdh6*:basGFP; ACry:GFP vector	Genscript/This study	N/A
*-70mafba*:basGFP; ACry:GFP vector	Genscript/This study	N/A
*+41lgmn*:basGFP; ACry:GFP vector	Genscript/This study	N/A
*+24nr2f2*:basGFP; ACry:GFP vector	Genscript/This study	N/A
*-64nr2f2*:basGFP; ACry:GFP vector	Genscript/This study	N/A
*+461mafba*:basGFP; ACry:GFP vector	Genscript/This study	N/A
*+47mafba*:basGFP; ACry:GFP vector	Genscript/This study	N/A
*+127tbx1*:basGFP; ACry:GFP vector	Genscript/This study	N/A
*Tfe3a* crRNA ATTTATGACGCTGATCGACT	IDT Alt-R^TM^ CRISPR-Cas9 System/This study	N/A
Alt-R™ CRISPR-Cas9 tracrRNA	IDT	Cat# 1072532
*Tfe3a* forward sequencing primer (M13 tag in capital letters) TGTAAAACGACGGCCAGccagctaagccgttcgttct	IDT/This study	N/A
*Tfe3a* reverse sequencing primer (PIGtail tag in capital letters) GTGTCTTgggtgaactatcccttccaagca	IDT/This study	N/A
**Chemicals, Enzymes and other reagents**		
DPBS	Thermo Fisher	Cat# 14190250
Paraformaldehyde	Sigma	Cat# P6148-1kg
Bovine Serum Albumin	Roche	Cat# 10735078001
CaCl_2_	Fisher Science	Cat# 15445389
FBS	Fisher Science	Cat# 11543407
EDTA	Fisher Science	Cat# 11568896
Sytox^TM^ Blue Dead Cell Stain	Thermo Fisher Scientific	Cat# S34857
Liberase	Sigma-Aldrich	Cat# 5401119001
Alt-R Cas9 Nuclease V3	IDT	Cat# 1081058
In-Fusion® HD cloning	Takara Bio	Cat# 639649
BalmHI	BioNordika	Cat# NEB-R0136S
SnaBI	BioNordika	Cat# NEB-R0130S
MAXIscript T7 Transcription Kit	Invitrogen	Cat# AM1312
Proteinase K	Thermo Fisher	Cat# AM2544
SSC 20X	Sigma	Cat# S6639-1L
Tween-20	Sigma	Cat# P9416
Formamide	Roche	Cat# 11814320001
Yeast tRNA	Thermo Fisher	Cat# 15401029
Heparin Sodium Salt from porcine	Sigma	Cat# H3149-50KU
Dextran Sulphate	Abcam	Cat# ab146569
Normal Goat Serum Control	Invitrogen	Cat# 31873
NBT	Roche	Cat# 11383213001
BCIP	Roche	Cat# 11383221001
TSA™ Plus Cyanine 3 System	Perkin Elmer	Cat# NEL744B001KT
Omnipaque	Apoteket	Cat# 019112
Agarose, low gelling temperature	Sigma	Cat# A9414
Ethyl 3-aminobenzoate methanesulfonate (Tricaine)	Sigma	Cat# E10521
**Software**		
Juicer v 1.6	Durand et al, 2016	N/A
OnTAD v 1.4	An et al, 2019	N/A
TADCompare	Cresswell and Dozmorov, 2020	N/A
GPU HiCCUPS	juicer_tools v 2.20.00	N/A
bedtools v 2.29.0	Quinlan and Hall, 2010	N/A
nf-core ATAC-sequencing pipeline v 1.2.1	Ewels et al, 2020	N/A
MACS2	Zhang et al, 2008	N/A
ATACseqQC	Ou et al, 2018	N/A
ChIPseeker	Yu et al, 2015	N/A
edgeR	Robinson et al, 2010	N/A
TOBIAS v 0.16.0	Bentsen et al, 2020	N/A
Cytoscape v 3.10.1	Shannon et al, 2003	N/A
deepTools v 3.3.2	Ramírez et al, 2016	N/A
pyGenomeTracks v 3.6	Lopez-Delisle et al, 2021	N/A
STAR v2.5.3a	Dobin et al, 2013	N/A
RSeQC v 2.6.1	Wang et al, 2012	N/A
rpkmforgenes	Ramsköld et al, 2009	N/A
Seurat v 5.1.0	Hao et al, 2021	N/A
ggplot2	Wickham, 2016	N/A
enrichplot	Yu G. enrichplot: Visualisation of Functional Enrichment Result. R package version 1.24.2,” 2024	N/A
Rstudio V 2023.12.1 + 402	Posit team, 2024; R Core Team, 2023	N/A
bedops v2.4.41	Neph et al, 2012	N/A
mVISTA	Brudno et al, 2003; Dubchak et al, 2000; Frazer et al, 2004; Mayor et al, 2000	N/A
Imaris v9.3.0	Oxford Instruments	N/A
ImageJ 2.9.0	Schneider et al, 2012	https://imagej.nih.gov/ij
Prism 9	Graphpad Software Inc.	N/A
NovaSeq Control Software 1.7.5/RTA v3.4.4	NovaSeq	N/A
NextSeq 1000/2000 Control Software 1.2.0.36376/RTA 3.7.17	NovaSeq	N/A
**Other**		
Arima-HiC kit	Arima Genomics	N/A
Nextera DNA CD kit	Illumina	N/A
Nextera XT index kits	Illumina	N/A

### Zebrafish

Zebrafish work was carried out under ethical approval from the Swedish Board of Agriculture (5.8.18-10590/2018 and 5.8.18-06282/2023). Zebrafish were housed at the Centre for In Vivo Uppsala University (CFVUU, Uppsala, Sweden) and adults and embryos were housed according to the standard laboratory procedure (Aleström et al, 2020).

### Cell collection for HiC and bulk ATAC-sequencing

Embryos of *Tg(prox1a:RFP)*^*nim5*^;*Tg(fli1a:EGFP)*^*y7*^ were collected at 5 days post-fertilisation (dpf). Isolation of cells was performed as previously described (Kartopawiro et al, 2014). For HiC, the crosslinking step of the Arima protocol was performed before sorting. Briefly, the dissociated cells were resuspended in 3% BSA in PBS, then incubated in 1.34% formaldehyde in PBS for 10 min at RT. Stop solution 1 was then added as per protocol, and the cells were incubated for 5 min at RT, then 15 min on ice. Cells were then washed by repeated centrifugation and resuspension in PBS and used for FACS sorting as described for ATAC-sequencing.

The dissociated cells were sorted using a FACS Aria III (BD Biosciences) directly into 300 μl PBS. We based the selection for the desired populations on FSC and SSC, singlets, and alive cells based on their Sytox^TM^ (Thermo Fisher) profile. Double-positive cells were then sorted for the *Tg(prox1a:RFP)*^*nim5*^ and *Tg(fli1a:EGFP)*^*y7*^ transgenes (LECs), whereas single-positive cells were sorted for the *Tg(fli1a:EGFP)*^*y7*^ transgene (BECs), as depicted in Fig. 1A. At least 50,000 cells per population were collected for each repeat. For bulk ATAC-sequencing, cells were then centrifuged and the supernatant removed, after which the tagmentation step was performed as described by the Nextera DNA CD protocol and the material was then snap-frozen in dry ice.

### HiC sequencing

Library preparation was performed using an Arima-HiC kit (Arima Genomics). Samples were sequenced on NovaSeq6000 (NovaSeq Control Software 1.7.5/RTA v3.4.4) with a 151nt(Read1)-10nt(Index1)-10nt(Index2)-151nt(Read2) setup using ‘NovaSeqXp’ workflow in ‘S4’ mode flowcell.

### HiC data analysis and TADs characterisation

Data were processed using Juicer 1.6 (Durand et al, 2016). The reference genome was GRCz11 with alt contigs removed. The restriction map was generated using the script generate_site_positions.py from Juicer version 1.6. The QC metrics and matrices of HiC contacts are derived from data containing reads aligned with MAPQ ≥30, after removal of duplicates and intra-fragment pairs. TADs were detected using OnTAD v 1.4 (An et al, 2019), with parameters: “-lsize 7 -penalty 0.075 -maxsz 300” on merged replicate matrices at bin size 10 kb. Reproducible TAD boundaries were identified using TADCompare (Cresswell & Dozmorov, 2020), interrogating TAD boundaries by comparing signal in replicates’ matrices (z_thres = 2.5, 500 kb window). Non-differential boundaries were considered reproducible. TADs delimited by two reproducible boundaries were labelled as reproducible. Differential boundaries between LEC and BEC were detected using TADCompare. TADs were stratified by length into three groups (<350 kb, 350kb-1Mb, >1 Mb) and the contacts were summarised at their boundaries in three window lengths (200, 350 and 500 kb), respectively, to account for TAD length. Boundaries passing cutoff were considered differential (z_thres = 1.8). TADs delimited by at least one differential boundary were labelled differential. For plotting overlapping TADs in Venn and Euler diagrams, TADs with boundaries within 30 kb in LECs and BECs were considered equivalent.

### Chromatin loops analysis

Chromatin loops were identified by GPU HiCCUPS (juicer_tools version 2.20.00) using default parameters. Loops from merged calls from 5, 10 and 25 kb matrix resolutions (output file merged_loops.bedpe) files were used in further analyses. Loops shared between replicates (tissue-reproducible loops) within a distance of 25 kb were identified using compare (juicer_tools version 1.9.9). Common and tissue-specific loops were identified by intersecting tissue-reproducible loops using bedtools v. 2.29.0 (Quinlan and Hall, 2010) (bedtools pairtopair -f 0.4; bedtools pairtopair -type notboth -slop 40,000, respectively). We required that common loops overlap at 0.4 of their lengths, and tissue-specific loops are separated by an interval of at least 40 kb.

Regions of accessible chromatin were annotated to the end of a loop when they fell within 10 kb of the loop matrix bin. Genomic features were assigned to the region of accessible chromatin as described in the 'Bulk ATAC-sequencing data analysis' section, and used to select the loop-mediated interactions of interest.

### Bulk ATAC-sequencing

Library preparation was performed using the Nextera DNA CD kit (Illumina). Samples were sequenced on NextSeq2000 (NextSeq 1000/2000 Control Software 1.2.0.36376/RTA 3.7.17) with a 76nt(Read1)-8nt(Index1)-8nt(Index2)-76nt(Read2) setup using ‘P2’ flowcell.

### Bulk ATAC-sequencing data analysis

Data were processed by the nf-core ATAC-sequencing pipeline v. 1.2.1 (Ewels et al, 2020). Peaks were called using MACS2 in broad mode with FDR cutoff 0.1 (Zhang et al, 2008). Quality control was performed using ATACseqQC (Ou et al, 2018) and peak annotation with Ensembl GRCz11 gene models was performed using ChIPseeker (Yu et al, 2015). Peaks were annotated to their closest genomic features, and regions up to 3 kb upstream of TSS were considered promoters. Peaks reproducible in at least two out of three replicates were merged to generate a consensus peak set used for differential accessibility analysis, after filtering out features with low counts. Counts were normalised by trimmed mean of *M* values (TMM) (Robinson and Oshlack, 2010) and differential accessibility (DA) analysis performed using edgeR (Robinson et al, 2010). FDR cutoff of 0.05 was applied to select significant differential peaks.

### Transcription factor footprinting and network construction

TF motifs were downloaded from JASPAR CORE 2024 vertebrate non-redundant collection. For Mafb-specific footprints, we also included Mafb motifs from (Jeong et al, 2017) and the Hocomoco MAFB.H12CORE.1.P.B motif (Kulakovskiy et al, 2018). TF site occupancy in consensus peaks was interrogated using TOBIAS (0.16.0) (Bentsen et al, 2020) in differential mode.

TF IDs were mapped to their zebrafish orthologs based on file ORTHOLOGY-ALLIANCE_COMBINED.tsv downloaded from www.alliancegenome.org.

The nodes of the TF networks were constructed from TOBIAS output files based on the annotation of the TF binding site in the target promoter (up to 3 kb from TSS). The nodes were selected where the target was expressed in 30% of the cells in a tissue-specific cluster in scRNA-sequencing data, with log2FC ≥0.5. The networks were visualised using Cytoscape v. 3.10.1 (Shannon et al, 2003).

### Genomic visualisations

Composite scaled ATAC-sequencing tracks were generated using deepTools (3.3.2) (Ramírez et al, 2016). The tracks were normalised to 1x average coverage and summarised to produce the average for three replicates. HiC signal was visualised as ICE-corrected merged replicate matrices at 5 kb resolution. Log2(LEC/BEC) HiC maps were generated after scaling the matrices to the same number of contacts using HiCExplorer (3.7.2) (Ramírez et al, 2018). Loop coordinates were converted from bedpe format to bed, including the outermost bin coordinates. The reproducible TADs were visualised on HiC heatmaps as triangles. Genomic visualisations were generated using pyGenomeTracks (3.6) (Lopez-Delisle et al, 2021).

### Zebrafish cell dissociation and fluorescence-activated cell sorting (FACS) for scRNA-sequencing

*Tg(-5.2lyve1b:Venus)*^*uu1kk*^*;Tg(fli1a:H2B-mCherry)*^*uq37bh*^ embryos were harvested at 5 dpf and 7 dpf. Heads were dissected by making an incision posterior to the otolithic lymph vessel. Trunks were dissected by making an incision posterior to the yolk and an incision posterior to the yolk extension. Isolation of cells was performed by dissociation of the whole embryos, heads and trunks following an optimised published protocol (Kartopawiro et al, 2014). Briefly, we deyolked collected or dissected embryos by pipetting up and down and rinsing in calcium-free Ringer’s solution. Then, we centrifuged at 2000 rpm for 5 min at 4 °C and removed the supernatant. Then, we dissociated the cells by incubating whole embryos, heads and trunks in 2.5 mg/ml Liberase (Sigma-Aldrich) diluted at a 1:35 ratio in DPBS at 28.5 °C. Whole 5 dpf and 7 dpf embryos were incubated for 9–10 min, whereas heads and trunks of 5 dpf and 7 dpf embryos were incubated for 7–8 min, homogenising the samples during and after the incubation. To stop the reaction, we added CaCl2 to a final concentration of 1–2 mM and FBS to a final concentration of 5–10%. We centrifuged at 2000 rpm for 5 min at 4 °C, discarding the supernatant. In order to be able to assess live vs. dead cells, we resuspend the cell solution in 1 μM Sytox^TM^ (Thermo Fisher Scientific) in DPBS/EDTA plus 0.5% FBS. The cell suspension was filtered through a strainer. The dissociated cells were sorted using a FACS Aria III (BD Biosciences) directly into buffered 384-well plates prepared by the Single Cell Core Facility of Flemingsberg Campus (SICOF) on a cold block. We based the selection for the desired populations on FSC and SSC, singlets, and alive cells based on their Sytox^TM^ (Thermo Fisher Scientific) profile. Double-positive cells were sorted for both *Tg(-5.2lyve1b:Venus)*^*uu1kk*^ and *Tg(fli1a:H2B-mCherry)*^*uq37bh*^ transgenes (LECs and VECs), as depicted in Fig. 2A. After sorting, the 384-well plates were snap-frozen on dry ice and stored at −80 °C before being shipped to the Single Cell Core Facility of Flemingsberg Campus (SICOF).

### scRNA-sequencing

Library preparation and sequencing was performed at the Single Cell Core Facility of Flemingsberg Campus (SICOF). Single-cell cDNA libraries were prepared according to the previously described Smart Seq2 protocol (Vanlandewijck and Betsholtz, 2018). Briefly, mRNA was transcribed into cDNA using oligo(dT) primer and SuperScript II reverse transcriptase (Thermo Fisher Scientific). Second-strand cDNA was synthetised using a template switching oligo. The synthetised cDNA was then amplified by polymerase chain reaction (PCR) for 23–26 cycles, depending on the tissue origin of the respective mRNA sample. Purified cDNA was quality controlled (QC) by analysing on a TapeStation 4200 or 2100 Bioanalyzer with a DNA High Sensitivity chip (Agilent Biotechnologies). When the sample passed the QC, the cDNA was fragmented and tagged (tagmented) using Tn5 transposase, and each single well was uniquely indexed using the Illumina Nextera XT index kits (Set A–D). Thereafter, the uniquely indexed cDNA libraries from one 384-well plate were pooled into one sample to be sequenced on one lane of a HiSeq3000 sequencer (Illumina), using dual indexing and single 50-base-pair reads.

The reads were aligned to the zebrafish genome (GRCz11) and spike-in sequences (from the External RNA Controls Consortium, ERCC92) using STAR v2.5.3a (Dobin et al, 2013), and filtered for uniquely mapping reads. Counts per gene were calculated for each transcript in Ensembl release 97 using rpkmforgenes (Ramsköld et al, 2009). Various QC metrics were generated with RSeQC 2.6.1 (Wang et al, 2012).

### scRNA-sequencing data analysis

Bioinformatics analyses were performed with the R programming language, using Seurat version 5.1.0 (Hao et al, 2021). Reads were normalised to transcripts per million reads (TPMs) in order to compensate for gene length in transcript count estimation. Values were then multiplied by the read length and rounded to the nearest integer. Cell libraries were considered of low quality and filtered out if: (1) the percentages of mitochondrial and ribosomal gene families were above 25%; (2) the percentage of protein-coding genes was below 90%; (3) the number of unique genes in a cell was below two standard deviations from the mean of the plate (Fig. EV2A,B). After filtering, the datasets contained 1264 cells for 5 dpf, and 1139 cells for 7 dpf. Genes detected in less than three cells were excluded from the analysis. For further downstream analysis, only protein-coding genes were used.

To remove batch differences, the Seurat SCT integration (Hafemeister and Satija, 2019; Stuart et al, 2019) pipeline was used. The dataset was divided based on the different anatomical parts of the zebrafish. Each subset was normalised individually, and variable features were identified. Based on these features, integration anchors were derived and used for integrating the subsets into one dataset used for subsequent analysis. Similarly, integration was performed for subdivisions of the dataset for the different developmental time points individually.

The datasets were further processed for identification of highly variable features and linear data compression using principal component analysis (PCA). Non-linear dimensionality reduction was run on the top 50 PCs to obtain a UMAP embedding. Unsupervised graph clustering was run using the Louvain algorithm with the modularity resolution parameter (res = 0.5). Cluster marker genes were identified using the Wilcoxon rank-sum test. Genes with an average logarithmic fold change of expression (log_2_FC) ≥0.25 at a Bonferroni-adjusted *p* value <0.01 were considered.

For all scRNA-sequencing datasets, exclusion of contamination clusters, as well as identification of the relevant LEC and VEC clusters, was determined using DEG and the expression patterns of the described markers. All downstream analysis and visualisation were performed using Seurat version 5.1.0 (Hao et al, 2021) with default settings, and utilising the ggplot2 (Wickham, 2016) R package and enrichplot ('Yu G. enrichplot: Visualisation of Functional Enrichment Result. R package version 1.24.2,' 2024) Bioconductor package.

### Enhancer identification

ATAC-based candidate enhancers were identified as peaks of open chromatin in LECs in the vicinity of genes whose expression was enriched in LECs at either 5 dpf or 7 dpf. HiC-based candidate enhancers were defined as differentially accessible regions of non-coding DNA which interact with the open promoter of a LEC-enriched gene through a LEC-specific loop. First, the coordinates of the left and right ends of LEC-specific loops, LEC-accessible open chromatin regions (OCRs) corresponding to TSS and intronic and distal peaks were retrieved. The open TSS and LEC-specific distal or intronic OCR closest to the loop anchors were identified using bedops (v 2.4.41) (Neph et al, 2012). Regions within 10 kb from loop anchors were considered to ensure their relation to the loop. Loops marked by an accessible TSS on one end, and an LEC-specific OCR on the other, were further refined by intersecting with the list of DE genes in LECs at 5 dpf and 7 dpf.

### Generation of transgenic lines

To test the activity of ATAC-based enhancers, the elements of interest were either cloned into the ZED vector as previously described (Bessa et al, 2009). For HiC-based enhancers, reporter vectors using a minimal E1b promoter as previously described (Panara et al, 2024) were ordered from GenScript. Sequences are in the Dataset EV22.

To generate mosaic transgenic lines, 1 μl of construct at 20 ng/μl and tol2 transposase mRNA at 100 ng/μl was injected into the one-cell stage *Tg(prox1a:RFP)*^*nim5*^ zebrafish embryos. Embryos were imaged in F0. At least four embryos per round of injection were imaged (Dataset EV23).

To generate F1 transgenic lines and assess the enhancer activity, 1 μl of construct at 20 ng/μl and tol2 transposase mRNA at 100 ng/μl was injected into the one-cell stage *Tg(prox1a:RFP)*^*nim5*^ zebrafish embryos and raised to adulthood. The F0 adults were outcrossed to *Tg(prox1a:RFP)*^*nim5*^, and F1 embryos were screened for expression. At least 2 injected adult F0 were screened per enhancer. Embryos with positive green lenses were imaged using a confocal microscope to determine enhancer-driven expression (Dataset EV23).

### Tfe3a CRISPR mutant generation

*Tfe3a* mutants were generated using the IDT Alt-R^TM^ CRISPR-Cas9 System. The guide was designed using IDT’s Predesigned Alt-R^TM^ CRISPR-Cas9 guide RNA Design Tool in the exon 5 of the *tfe3a* gene. Primers were designed upstream and downstream of the guide site as described in the protocol originally developed in the Burgess Lab (Carrington et al, 2015) and adapted for the DanioReadout facility (Habicher et al, 2022).

Zebrafish embryos were injected at the one-cell stage with 50–100 ng/μL of gRNA complex (crRNA plus tracrRNA) and 200 ng/μL Alt-R Cas9 Nuclease V3. F0 fish were raised to adulthood and outcrossed to wildtype. F1 embryos from three individual crosses were raised. F1 fin clips were genotyped using Fragment Length Analysis. A founder containing an insertion of 13 bp was confirmed by Sanger sequencing, and a stable line was established.

### In situ probe synthesis

Probes were generated by In-Fusion® HD cloning (Takara Bio) to the PCS2+ vector using the primers listed in Dataset EV24. Vectors were then linearised with BalmHI for antisense probes and SnaBI for sense probes, prior to probe synthesis, was then carried out using the MAXIscript T7 Transcription Kit (Invitrogen).

### In situ hybridisation

Embryos of the desired stage were dechorionated prior to fixation in 4% paraformaldehyde/PBS for 3 h at room temperature or overnight at 4 °C. Fixed embryos were dehydrated with progressive washes of 50% MeOH, 75% MeOH and 100% MeOH, then stored in 100% methanol at −20 °C. Embryos at 2 dpf were incubated in 10 mg/ml PK in PBST for 30 min at RT, and embryos at 5 dpf were incubated in 20 mg/ml PK in PBST for 30 min at RT. Subsequently, embryos were fixed in 4% PFA for 20 min prior to processing by in situ hybridisation as previously described by (Xu et al, 1994) with BCIP/NBT. After colour development, embryos were fixed in 4% PFA for 20 min then cleared in 75% glycerol/PBS, and mounted for imaging using a Leica Fluorescent Stereo Microscope M165 FC and 1x objective (objective number: 10450028). Embryos were mounted in 3% methylcellulose.

### Fluorescent in situ hybridisation

The protocol for standard in situ hybridisation was followed until the blocking step. From here, embryos were blocked in 2 mg/ml BSA (Sigma)/2% sheep serum (Thermo Fisher) for 3 h, prior to antibody incubation in 1:5000 anti-digoxigenin fluorescein POD (Roche) and 1:400 Chicken Anti-GFP (Abcam) at 4 °C for 16 h. Embryos were washed 8 times in PBST for 30 min prior to incubation with 1:100 tyramide-Cy3 in 1x amplification diluent (Perkin Elmer). Embryos were washed a further eight times in PBST for 30 min prior to blocking in 2 mg/ml BSA (Sigma)/2% sheep serum (Thermo Fisher) for a minimum of 1 h. Embryos were incubated in 1:400 Anti-chicken 488 (Jackson Immuno Research) at 4 °C for 16 h. Embryos were then washed eight times in PBST for 15 min prior to fixation in 4% PFA for 30 min. Embryos were imaged on a Leica TCS SP8 DLS with HC PL APO CS2 40× water objective (objective number: 11506360).

### Immunostainings

Embryos were fixed at 4 dpf overnight at 4 °C, and a previously described protocol was followed (Koltowska et al, 2015a; Shin et al, 2002). We applied the following modification: the Proteinase K treatment was performed at 20 μg/ml for 35 min, and blocking prior to primary antibody was done for 48 h. For primary antibodies, Chicken anti-GFP (1:400, Abcam) and Rabbit anti-Prox1 (1:500, AngioBio Co.) were used and for secondary antibodies, anti-Rabbit IgG-HRP (1:1000 CellSignalling Technology) and anti-Chicken Alexa-488 (1:200 703-545-155, Jackson Immuno Research)) followed by the TSA™ Plus Cyanine 3 System amplification according to the manufacturer's instructions (NEL744001KT Perkin Elmer) and DAPI (1:1000 D1306 Thermo Fisher Scientific) staining. The trunks were mounted in the clearing solution Omnipaque (350 mg litre^–1^ concentration per 1 ml iohexol, GE Healthcare) for imaging.

### Imaging, image processing and quantification

Embryos for in vivo fluorescent imaging were anesthetised with tricaine and mounted in 1% low melting agarose. Embryos were imaged using a Leica TCS SP8 DLS inverted confocal microscope with a Fluotar VISR 25X water objective (objective number: 11506375). The 488 nm (for GFP) and 552 nm (for RFP or DsRed) laser lines were used. For the trunk vasculature, the images were taken between 8–15 somites across the width of the fish, generating a full z-stack. For facial lymphatic vessels, the whole z-stack consisted of the vessels on one side of the fish.

Masking was performed in Imaris v9.3.0 with a surface detail of 1μm. Images were processed using ImageJ 2.9.0 (Schneider et al, 2012). All representative images are Maximum Intensity Projections.

Manual quantification of lymphatic vessels, vIVS and ectopic vessel cell numbers was performed using full z-stacks. The number was counted using the overlay of DsRed- and GFP-channels over two somites in the trunks. The vessel type was distinguished based on the morphology (Okuda et al, 2012). Quantification was performed by scoring the amount of nuclei from corresponding vessel types for each genotype, as previously described (Arnold et al, 2022). Quantification of Prox1-positive lymphatic endothelial cells was conducted using a full z-stack of trunk vessels, based on the co-presence of *nfli:GFP* and Prox1 signal. The double-positive GFP and Prox1 nuclei were recorded for thoracic duct and intersegmental lymphatic vessels. Single-blinding with regard to genotype was used during quantifications.

### Statistical analysis

Unless otherwise stated, data processing and statistical analysis of RNA-seq, ATAC-seq, and HiC was performed in R (Posit team, 2024; R Core Team, 2023).

Fisher’s exact test was used to investigate the overlap of differential TADs with LEC and BEC enriched gene loci, differentially expressed genes (Fig. EV2G).

Positional association of differential TADs and boundaries with ATAC peaks and CTCF occupancy was performed using permutation tests as previously described (Malinverni et al, 2023), randomising TAD intervals (*n* = 5000 permutations).

For loop interaction classification (Fig. S3B–D), the expected distribution of features in loops was calculated based on the frequency of the features in either the open chromatin peaks or the differentially accessible peaks from bulk ATAC-sequencing analysis. The expected distribution was compared with the observed one with a chi-square test for goodness of fit using Prism 9 (Graphpad). Observed distributions in LECs and BECs were compared using a chi-square test. Residuals were calculated by computing the expected number of loops in each category and subtracting the value from the observed count. For multiple group comparisons (Fig. 7D,E) the normality was tested with a Shapiro–Wilk test, all quantification but ectopic vessels passed the normality test. The ordinary one-way ANOVA test was used for normally distributed data, Kruskal–Wallis test was used for non-normally distributed data. All error bars represent the standard error of the mean (SEM).

## Supplementary information

Appendix

Peer Review File

Dataset EV1

Dataset EV2

Dataset EV3

Dataset EV4

Dataset EV5

Dataset EV6

Dataset EV7

Dataset EV8

Dataset EV9

Dataset EV10

Dataset EV11

Dataset EV12

Dataset EV13

Dataset EV14

Dataset EV15

Dataset EV16

Dataset EV17

Dataset EV18

Dataset EV19

Dataset EV20

Dataset EV21

Dataset EV22

Dataset EV23

Dataset EV24

Source data Fig. 2

Source data Fig. 3

Source data Fig. 4

Source data Fig. 5

Source data Fig. 6

Figure EV2 Source Data

Figure EV5 Source Data

Appendix Figures Source Data

Expanded View Figures

## Data Availability

ScRNA-sequencing data were available on ArrayExpress (accession number: E-MTAB-16797, https://www.ebi.ac.uk/biostudies/ArrayExpress/studies/E-MTAB-16797?key=06ce094c-9d03-4d6f-a685-6e1f7a30dc3e) and for consultation, the Shiny app is hosted at https://lymphatic-proliferation-zebrafish.serve.scilifelab.se/app/lymphatic-proliferation-zebrafish. HiC and ATAC-sequencing data were available on ArrayExpress (accession number: E-MTAB-16939, https://www.ebi.ac.uk/biostudies/ArrayExpress/studies/E-MTAB-16939?key=52845bed-8dc8-4c06-aee2-60c8b41a4409), and UCSC Genome browser tracks are available at https://github.com/LymphaticsLab/Panara-Arnold-Gloger-2026-tracks. The code used for the analysis are available at https://github.com/NBISweden/NBIS-5931-lymphatics-zebrafish. The vectors utilised to generate the zebrafish transgenic lines are available upon reasonable request to the corresponding author. The source data of this paper are collected in the following database record: biostudies:S-SCDT-10_1038-S44319-026-00895-1.
