# Supplementary material for: 3D chromatin organisation contributes to the regulatory logic in differentiating lymphatic endothelium
Source: EMBO Rep. 2026 Aug 14;27(18):5262–91. doi: 10.1038/s44319-026-00895-1 (PMC13601605; doi:10.1038/s44319-026-00895-1)
Supplement: Supplementary file 1 [file 44319_2026_895_MOESM1_ESM.pdf]

Appendix to 3D chromatin organisation contributes to the regulatory logic in differentiating lymphatic endothelium

Table of content:

Appendix Figure S1: Additional characterisation of four novel ATAC-based LEC enhancers. (related to Figure 4)……………………………….…………………Page 2

Appendix Figure S2: Full-body images of stable and transient enhancer reporter lines (related to Figures 4, 5, 6, EV5)……………………………….…………………Page 4

Appendix Figure S3: Additional characterisation of novel HiC-based LEC enhancers (related to Figure 5)…………….……………………………….…………………Page 6

Appendix Figure S4: Additional characterisation of the mafba downstream regulatory network in LECs and BECs (related to Figure 7) ……………………..…………….……………………………….…………………Page 8

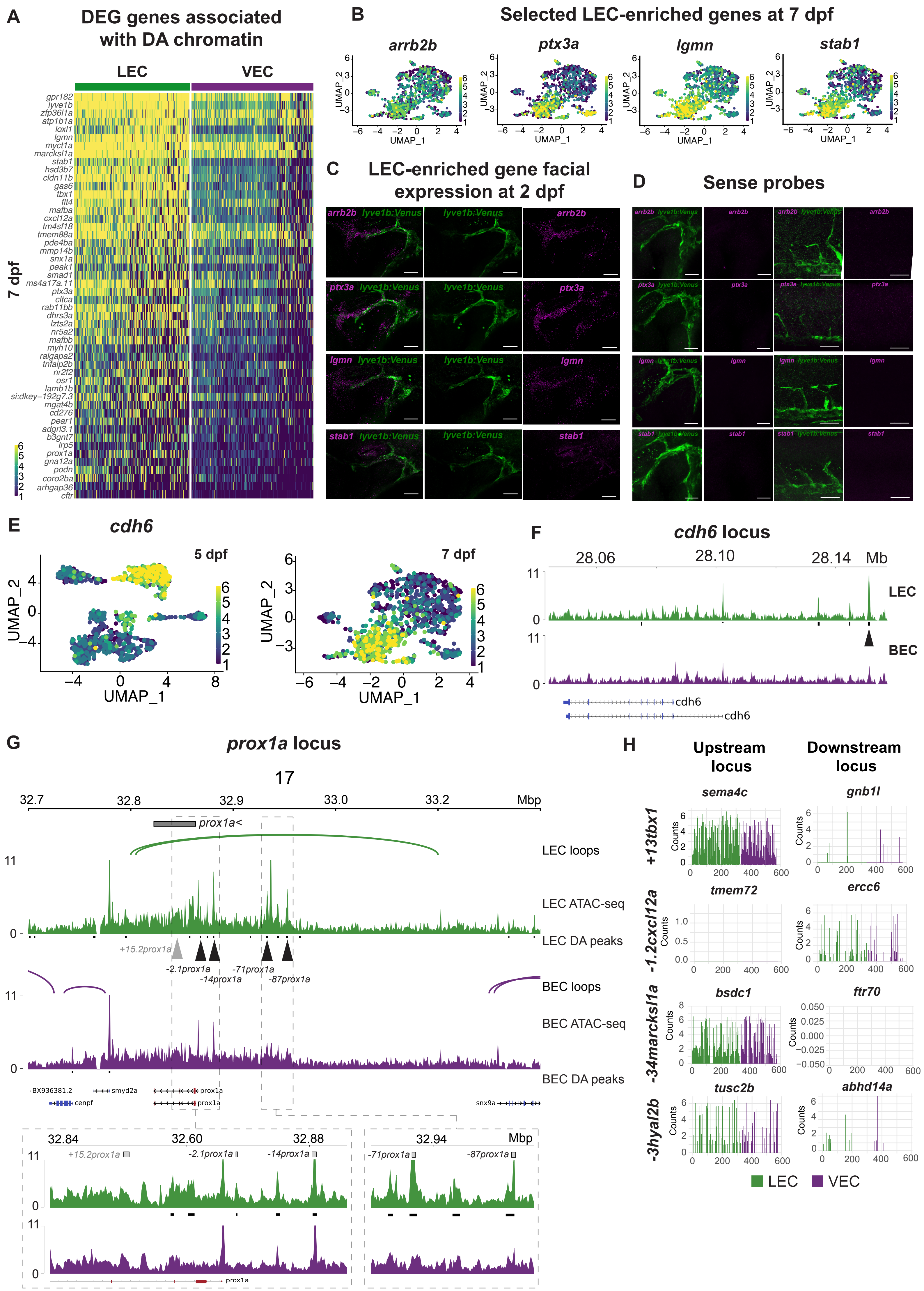
A
DEG genes associated with DA chromatin
LEC
VEC
7 dpf
gpr182
lyve1b
zfp36l1a
atp1b1a
loxl1
lgmn
myct1a
marcksl1a
stab1
hsd3b7
cldn11b
gas6
tbx1
flt4
mafba
cxcl12a
tm4sf18
tmem88a
pde4ba
mmp14b
snx1a
peak1
smad1
ms4a17a.11
ptx3a
cltca
rab11bb
dhrs3a
lzts2a
nr5a2
mafbb
myh10
ralgapa2
tnfaip2b
nr2f2
osr1
lamb1b
si:dkey-192g7.3
mgat4b
cd276
pear1
adgrl3.1
b3gnt7
lrp5
prox1a
gna12a
podn
coro2ba
arhgap36
cftr
B
Selected LEC-enriched genes at 7 dpf
arrb2b
ptx3a
lgmn
stab1
UMAP_1
UMAP_2
C
LEC-enriched gene facial expression at 2 dpf
arrb2b lyve1b:Venus
lyve1b:Venus
arrb2b
ptx3a lyve1b:Venus
ptx3a
lgmn lyve1b:Venus
lgmn
stab1 lyve1b:Venus
stab1
D
Sense probes
E
cdh6
5 dpf
7 dpf
UMAP_1
UMAP_2
F
cdh6 locus
28.06
28.10
28.14
Mb
LEC
BEC
cdh6
cdh6
G
prox1a locus
17
32.7
32.8
32.9
33.0
33.2
Mbp
prox1a<
LEC loops
LEC ATAC-seq
LEC DA peaks
+15.2prox1a
-2.1prox1a
-14prox1a
-71prox1a
-87prox1a
BEC loops
BEC ATAC-seq
BEC DA peaks
BX936381.2
smyd2a
cenpf
prox1a
prox1a
snx9a
32.84
32.60
32.88
32.94
Mbp
H
Upstream locus
Downstream locus
+13tbx1
sema4c
gnb1l
-1.2cxcl12a
tmem72
ercc6
-34marcksl1a
bsdc1
ftr70
-3hyal2b
tusc2b
abhd14a
Counts
LEC
VEC

Appendix Figure S1: Additional characterisation of four novel ATAC-based LEC enhancers (related to Figure 4).

(A) scRNA-seq heatmap showing LECs and VECs expression of genes associated with the top DA peaks at 7 dpf. Cells ordered by clusters. Colour indicates log-normalized read counts.

(B) Expression of the genes selected for *in vivo* validation at 7 dpf.

(C) Fluorescent *in situ* hybridisation (N = 45) showing lymphatic expression of the selected genes in the face at 2 dpf. *In situs* were performed in three technical repeats.

(D) Sense-probe control *in situ* hybridisation (N= 45), showing no endothelial signal at 2 dpf. Control *in situs* were performed in two technical repeats.

(E) LEC-enriched *cdh6* expression at 5 dpf and 7 dpf. Colour scale indicates normalised expression.

(F) *cdh6* chromatin accessibility in LECs and BECs. Black boxes: DA peaks. Arrow: -*48cdh6* enhancer.

(G) *prox1a* chromatin accessibility in LECs and BECs. LEC-specific loops in green, BEC-specific loops in purple. Black boxes: DA peaks. Black arrows: previously identified *-2.1prox1a*, *-14prox1a*, *-71prox1a* and *-87prox1a* enhancers, marked by DA peaks. Grey arrow: previously identified *+15.2prox1a* enhancer. Grey boxes: zoomed-in view of the ATAC-seq tracks.

(H) Cell counts of genes upstream and downstream of the identified enhancers, in LECs and VECs at 5 dpf.

Scale bars: 50 µm.

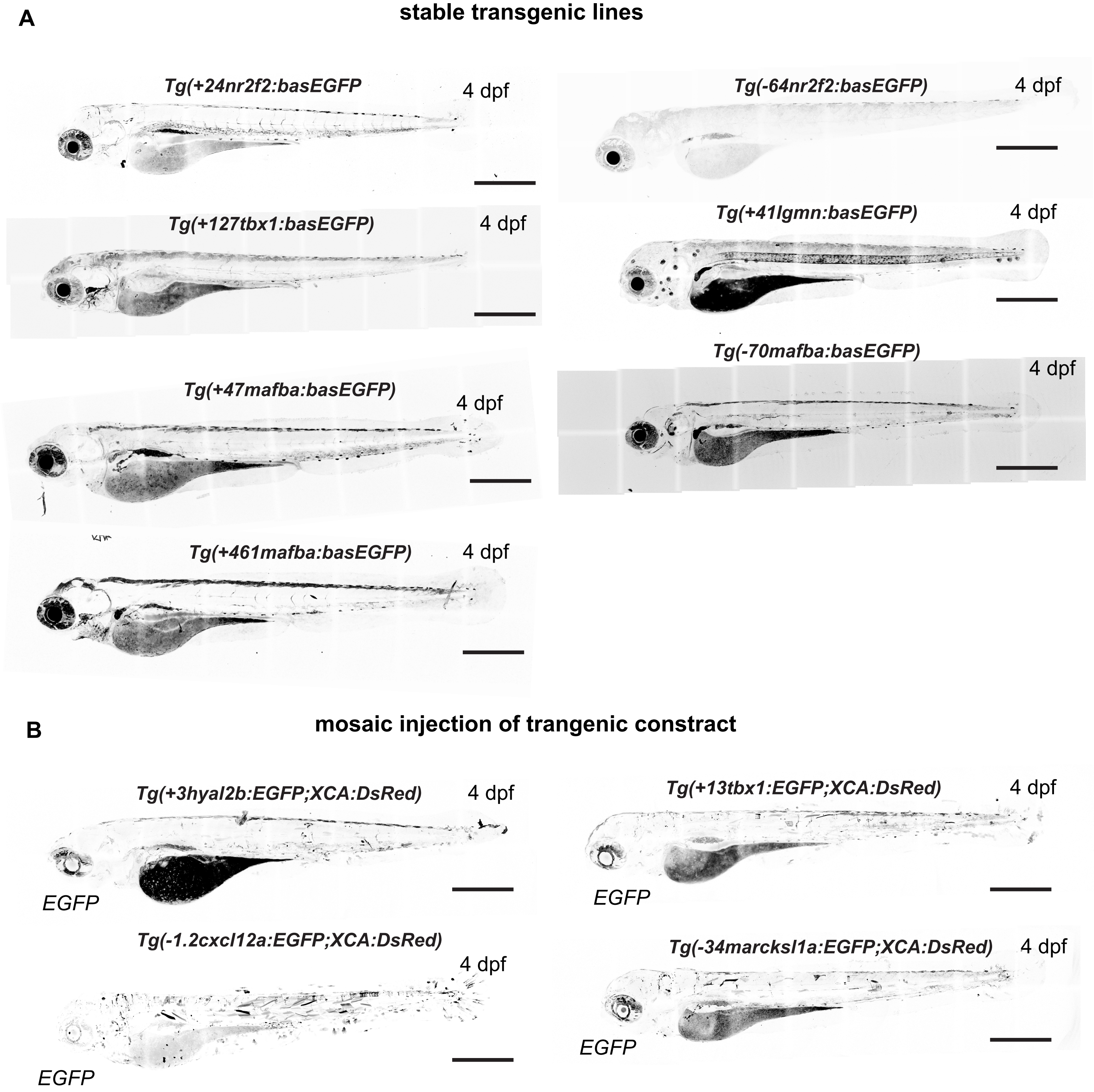
A
stable transgenic lines
Tg(+24nr2f2:basEGFP
4 dpf
Tg(-64nr2f2:basEGFP)
4 dpf
Tg(+127tbx1:basEGFP)
4 dpf
Tg(+41lgmn:basEGFP)
4 dpf
Tg(-70mafba:basEGFP)
4 dpf
Tg(+47mafba:basEGFP)
4 dpf
Tg(+461mafba:basEGFP)
4 dpf
B
mosaic injection of trangenic constract
Tg(+3hyal2b:EGFP;XCA:DsRed)
4 dpf
EGFP
Tg(+13tbx1:EGFP;XCA:DsRed)
4 dpf
EGFP
Tg(-1.2cxcl12a:EGFP;XCA:DsRed)
4 dpf
EGFP
Tg(-34marcksl1a:EGFP;XCA:DsRed)
4 dpf
EGFP

Appendix Figure S2: Full-body images of stable and transient enhancer reporter lines (related to Figures 4, 5, 6, EV5).

A) Maximum projection of full-body signal in stable transgenic 4 dpf embryos of enhancer reporter lines generated for this study.

B) Maximum projection of full-body signal in injected mosaic 4 dpf embryos of enhancer reporter lines generated for this study.

Scale bar: 500 µm. Skin signal has been removed to allow visualisation in deeper tissues.

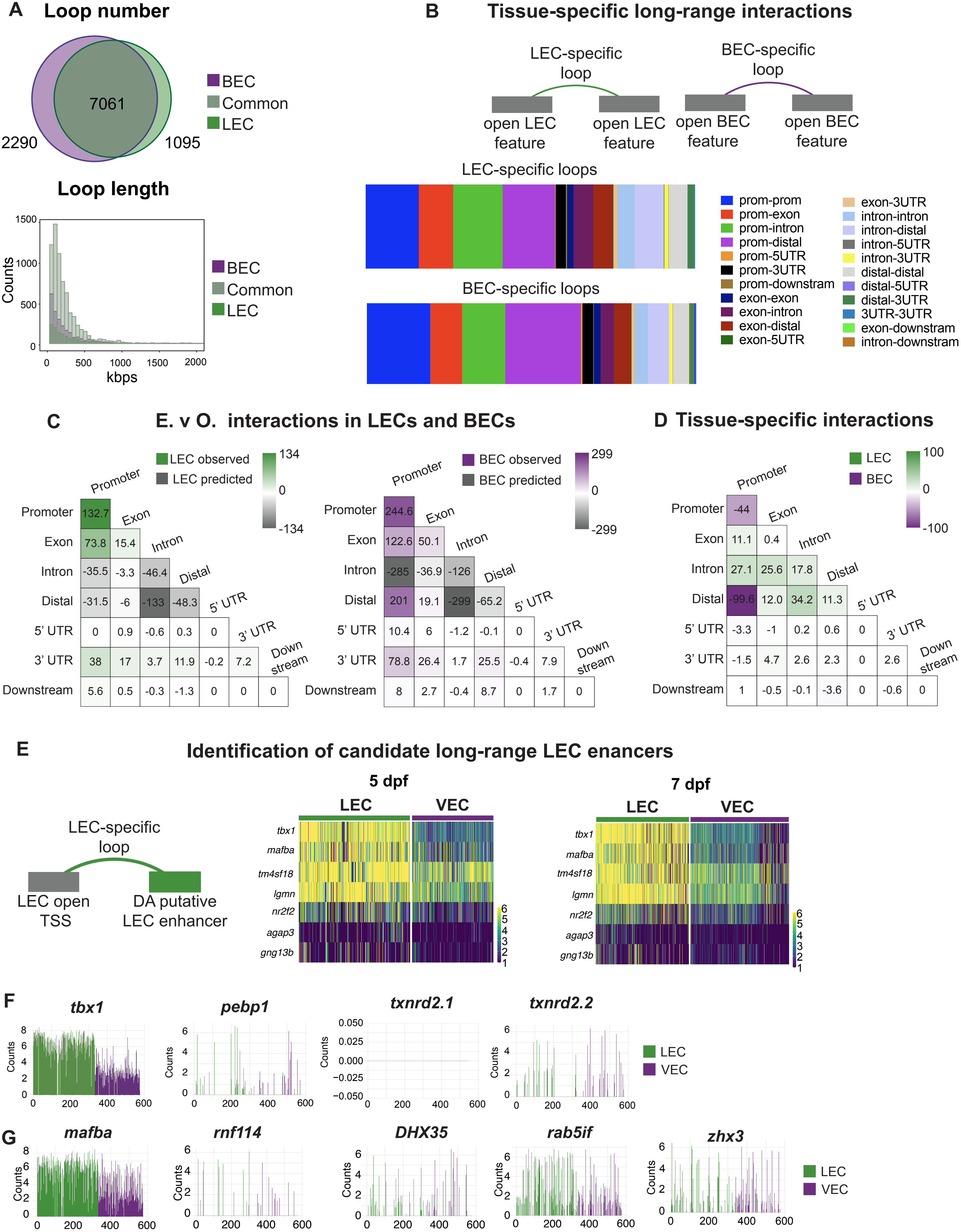
A
Loop number
7061
2290
1095
BEC
Common
LEC
Loop length
Counts
kbps
B
Tissue-specific long-range interactions
LEC-specific loop
open LEC feature
open LEC feature
BEC-specific loop
open BEC feature
open BEC feature
LEC-specific loops
BEC-specific loops
prom-prom
prom-exon
prom-intron
prom-distal
prom-5UTR
prom-3UTR
prom-downstream
exon-exon
exon-intron
exon-distal
exon-5UTR
exon-3UTR
intron-intron
intron-distal
intron-5UTR
intron-3UTR
distal-distal
distal-5UTR
distal-3UTR
3UTR-3UTR
exon-downstream
intron-downstream
C
E. v O. interactions in LECs and BECs
LEC observed
LEC predicted
Promoter
Exon
Intron
Distal
5' UTR
3' UTR
Down stream
Downstream
132.7
73.8 15.4
-35.5 -3.3 -46.4
-31.5 -6 -133 -48.3
0 0.9 -0.6 0.3 0
38 17 3.7 11.9 -0.2 7.2
5.6 0.5 -0.3 -1.3 0 0 0
BEC observed
BEC predicted
244.6
122.6 50.1
-285 -36.9 -126
201 19.1 -299 -65.2
10.4 6 -1.2 -0.1 0
78.8 26.4 1.7 25.5 -0.4 7.9
8 2.7 -0.4 8.7 0 1.7 0
D
Tissue-specific interactions
LEC
BEC
-44
11.1 0.4
27.1 25.6 17.8
-99.6 12.0 34.2 11.3
-3.3 -1 0.2 0.6 0
-1.5 4.7 2.6 2.3 0 2.6
1 -0.5 -0.1 -3.6 0 -0.6 0
E
Identification of candidate long-range LEC enancers
LEC-specific loop
LEC open TSS
DA putative LEC enhancer
5 dpf
7 dpf
LEC
VEC
tbx1
mafba
tm4sf18
lgmn
nr2f2
agap3
gng13b
F
tbx1
pebp1
txnrd2.1
txnrd2.2
Counts
LEC
VEC
G
mafba
rnf114
DHX35
rab5if
zhx3
Counts
LEC
VEC

Appendix Figure S3: Additional characterisation of novel HiC-based LEC enhancers (related to Figure 5).

(A) Characterisation of chromatin loops in LECs and BECs. Top: quantification of tissue-specific and common loops. Bottom: length distribution of these loops.

(B) Tissue-specific long-range interactions. Left: schematic of the considered features. Right: Frequency of each interaction type in LECs and BECs.

(C) Residual map of the observed vs expected interaction types within each cell population. Chi-square test, positive values indicate enrichment; negative values indicate depletion.

(D) Quantification of tissue-specific interactions. Chi-square test residuals show enrichment of different interaction types in LECs and BECs. Positive and negative values indicate enrichment in LECs and BECs, respectively. Scale: percentage of residuals for the interaction.

(E) Identification of candidate HiC-based enhancers. Left: Schematics of selection criteria. Right: Heatmaps showing expression in LECs and VECs at 5 dpf and 7 dpf. Cells ordered by clusters. Viridis scale indicates log-scale normalised read counts.

(F) Cell counts of gene loci surrounding *tbx1* in LEC and VEC clusters at 5 dpf, showing enriched expression at selected loci.

(G) Cell counts of gene loci around *+461mafba* in LEC and VEC clusters at 5 dpf, showing enriched LEC expression of *mafba*.

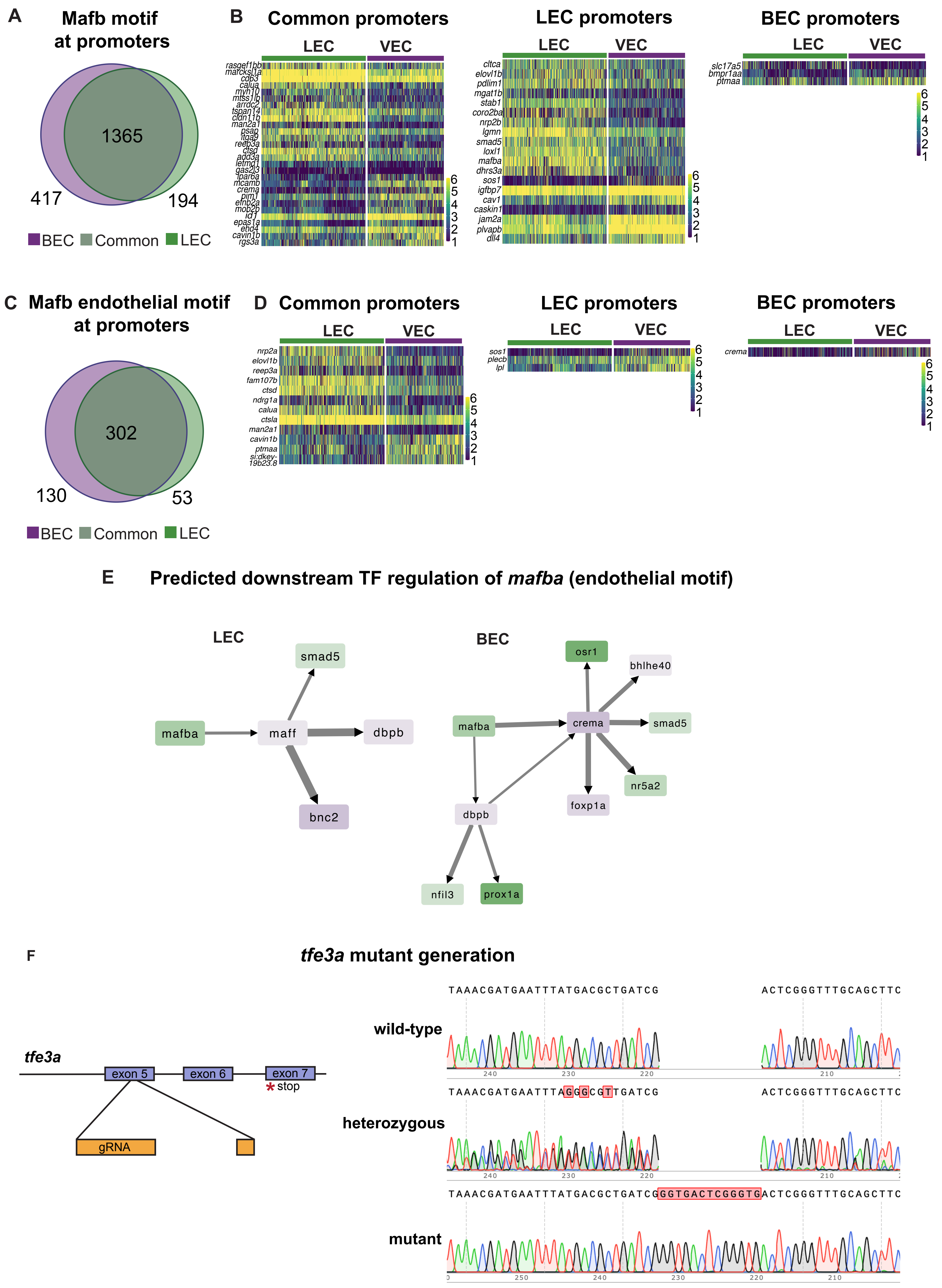
A
Mafb motif at promoters
1365
417
194
BEC Common LEC
B
Common promoters
LEC VEC
rasgef1bb
marcksl1a
cd63
calua
myh10
mtss1lb
arrdc2
tspan14
cldn11b
man2a1
psap
itga9
reep3a
ctsd
add3a
letmd1
gas2l3
lparba
mcamb
crema
pim1
efnb2a
mob2b
id1
epas1a
ehd4
cavin1b
rgs3a
LEC promoters
LEC VEC
cltca
elovl1b
pdlim1
mgat1b
stab1
coro2ba
nrp2b
lgmn
smad5
loxl1
mafba
dhrs3a
sos1
igfbp7
cav1
caskin1
jam2a
plvapb
dll4
BEC promoters
LEC VEC
slc17a5
bmpr1aa
ptmaa
6 5 4 3 2 1
C
Mafb endothelial motif at promoters
302
130
53
BEC Common LEC
D
Common promoters
LEC VEC
nrp2a
elovl1b
reep3a
fam107b
ctsd
ndrg1a
calua
ctsla
man2a1
cavin1b
ptmaa
si:dkey-19b23.8
LEC promoters
LEC VEC
sos1
plecb
lpl
BEC promoters
LEC VEC
crema
6 5 4 3 2 1
E
Predicted downstream TF regulation of mafba (endothelial motif)
LEC
mafba
maff
smad5
dbpb
bnc2
BEC
mafba
crema
osr1
bhlhe40
smad5
nr5a2
foxp1a
dbpb
nfil3
prox1a
F
tfe3a mutant generation
tfe3a
exon 5
exon 6
exon 7
* stop
gRNA
wild-type
TAAACGATGAATTTATGACGCTGATCG
ACTCGGGTTTGCAGCTTC
heterozygous
TAAACGATGAATTTAGGGCGTTGATCG
ACTCGGGTTTGCAGCTTC
mutant
TAAACGATGAATTTATGACGCTGATCGGGTGACTCGGGTGACTCGGGTTTGCAGCTTC
240 230 220 210
250 240 230 220 210

Appendix Figure S4: Additional characterisation of the *mafba* downstream regulatory network in LECs and BECs (related to Figure 7).

(A) Quantification of accessible promoters in LECs, BECs and common predicted to be bound by Mafb.

(B) Heatmaps of differentially expressed genes (log2FC >= 0.3 or log2FC <= -0.3) marked by predicted Mafba occupancy in the promoter region at 5 dpf. Left: Mafba predicted occupancy in LECs and BECs. Centre: LEC-specific Mafba predicted occupancy. Right: BEC-specific Mafba predicted occupancy.Cells ordered by clusters. Colour scale indicates log-scale normalised read counts.

(C) Quantification of accessible promoters in LECs, BECs and in common predicted to be bound by Mafb using the endothelial motif.

(D) Heatmaps showing differentially expressed genes (log2FC >= 0.3 or log2FC <= -0.3) marked by predicted Mafba occupancy in their promoter region at 5 dpf. Left: Mafba predicted occupancy in both LECs and BECs. Centre: LEC-specific Mafba predicted occupancy. Right: BEC-specific Mafba predicted occupancy. Cells ordered by clusters. Colour scale clour indicates log-scale normalised read counts.

(E) Two-layered TF networks downstream of *mafba* in LEC and BEC, based on the TF footprinting results using the endothelial motif. Green: expression enriched in LECs at 5 dpf. Purple: expression enriched in BECs at 5dpf.

(F) *tfe3a* CRISPR mutant generation. The introduction of a 13 bp insertion created a premature stop codon in exon 7 (151 bp downstream of the mutation in the mRNA). Validation of the 13 bp insertion by Sanger sequencing showing wild-type, heterozygous, and mutant sequences.
