# Supplementary material for: 3D chromatin organisation contributes to the regulatory logic in differentiating lymphatic endothelium
Source: EMBO Rep. 2026 Aug 14;27(18):5262–91. doi: 10.1038/s44319-026-00895-1 (PMC13601605; doi:10.1038/s44319-026-00895-1)
Supplement: Supplementary file 2 [file 44319_2026_895_MOESM2_ESM.pdf]

EMBO reports

3D chromatin organisation contributes to regulatory logic in differentiating lymphatic endothelium

Virginia Panara, Hannah Arnold, Marleen Gloger, Renae Skoczylas, Victoria Vidal Gutierrez, Anna Johansson, Agata Smialowska, and Katarzyna Koltowska

Corresponding author(s): Katarzyna Koltowska (kaska.koltowska@igp.uu.se)

Review Timeline:

Transfer Date:	3rd Oct 25
Editorial Decision:	5th Nov 25
Revision Received:	17th Mar 26
Editorial Decision:	22nd May 26
Revision Received:	24th Jun 26
Accepted:	20th Jul 26

Editor: Kurt Weir

Transaction Report: This manuscript was transferred to EMBO Reports following peer review at Review Commons.

Review COMMONS

(Note: With the exception of the correction of typographical or spelling errors that could be a source of ambiguity, letters and reports are not edited. Depending on transfer agreements, referee reports obtained elsewhere may or may not be included in this compilation. Referee reports are anonymous unless the Referee chooses to sign their reports.)

Review #1

1. Evidence, reproducibility and clarity:

Evidence, reproducibility and clarity (Required)

Summary

In this paper, Panara and colleagues utilize both Hi-C and ATAC-seq techniques to profile chromatin organisation in zebrafish lymphatic endothelial cells, and to compare this to that of blood endothelial cells from 5-7 day old embryos. They focus on genes differentially expressed in lymphatic ECs, using changes in chromatin openness to identify novel enhancers and infer regulatory logics. They also look at the downstream targets of mafba, and describe a novel interaction with tfe3a to limit ectopic vascularisation. Overall, this paper contains an impressive array of useful and informative data, although this may be occasionally over-interpreted in the discussion of results.

Major comments:

1. I do not doubt the bioinformatics underpinning the analysis in Figure 2, but the TAD organisational changes shown in 2E-F look slight, to the point that I would not have noted them without the blue lines. Given that e.g. prox1a is expressed in LECs only, it is hard to appreciate that such a minimal change in chromatin structure could lead to such extreme changes in expression. I was also surprised that the lower order TAD domains changed so little. This also appears true in Figure 5 although potentially to a lesser extent. I would have been interested to have had this (and the ATAC-seq data) discussed in a more agnostic manner - would this level of chromatin change have been expected, how does it compare to similar analysis in non-EC cell types (e.g. smooth muscle cells after activation), what does this say about gene regulation per se. As it is, I did not feel that the text fully connected with the images of the results.
2. Related to Figure 2, is the Hi-C data able to establish if promoter contacts change significantly between LECs and BECs for genes that are differentially expressed? E.g. well-studied genes such as prox1a or mafba, which have defined lymphatic enhancers also?
3. It is unclear how much value can be placed on differential openness in identifying novel lymphatic genes (Figure 3 D), since the analysis was limited to genes differentially expressed in LECs, and did not test the lymphatic-specificity of regions near DEG that were not differentially open (e.g. equally open in both cell types but near a lymphatic only gene). In fact, the data presented here seems to indicate that differential openness often has little correlation with gene expression. I think this is interesting (alongside the Hi-C data), not

problematic. Within the endothelial field there are papers suggesting chromatin openness is and isn't associated with differential enhancer activity within different subtypes (e.g. Sissaoui et al 2020 suggests it is associated; Nornes et al 2025 suggests it is not), it would be interesting to discuss how the data here fits this discussion.

4. Figure 4 utilizes the ATAC-seq dataset to identify novel enhancers. It would also be interesting to see the results around previously identified lymphatic enhancers in addition to the cdh6 enhancer (this also applies to later chromatin loop data). Previous work by the senior author identified a variety of lymphatic-specific enhancers for prox1a, would it be possible to clearly annotate within this paper the ATAC-seq/HiC/chromatin looping landscape around these validated enhancers?
5. It would be beneficial to the reader to show images of entire transient and stable transgenic zebrafish expressing the various enhancer:GFP constructs (potentially in the supplement).
6. The MAFBA binding motif is shared across the MAF family and is fairly broad, therefore I have significant reservations about the analysis in Figure 7. The text should clarify that analysis is on motifs, not directly assessing protein binding, and should explain why the focus is on MAFBA binding at promoters only. This part is also poorly validated, and does not really improve the paper.
7. I am unconvinced about the rational to look at Mafba/Tfe3a cooperation (the last part of the results), as this feels unconnected to the rest of the paper (e.g. there does not appear to be a link to chromatin occupation/motifs).
8. It would be usefull to more clearly discuss the number of differential consensus TADs between LECs and BECs in comparison with the number of differentially expressed genes between these two cell types. The discussion states that a large number of TADs show differences between LECs and BECs, but reading the paper this was not my impression. It would therefore help to know what percentage of differentially regulated genes had clear TAD boundary differences when discussing this (I apologise if I missed this information).
9. In general, there is a lot of different analyses here, and it would potentially be beneficial to remove some (e.g. TF motif search within characterised enhancers via TOBIAS, transcriptional signatures from ATAC-seq data) as it is not validated to the level required for publication yet neither is it essential for the paper and the underlying message.
10. I would have appreciated (in the Discussion) a more agnostic discussion of the chromatin changes between BEC and LECs - do all DEGs have differences in chromatin openness or organisation? Are all differentially open regions enhancers? Do some loci have differentially open chromatin but no detectable changes in TADs, and vice versa? And why? But

Minor comments:

In Figure 5, the locations of the enhancers on the HiC images are not clear.

I am unsure of the utility of listing TFs with potential motifs within each enhancer, and believe this could be left out without impacting the paper. This type of prediction is very dependent on the program used and the settings used. If the authors prefer to keep this information, it should be annotated onto the sequence of the enhancers alongside the information provided by the prediction software (e.g. stringency, strength of fit to consensus).

The expression pattern of the +24nr2f2 enhancer does not mimic nr2f2, this could be discussed?

The chromatin accessibility plots for mafba appear to vary in height, and in regions marked as DA, between Figure 6C and G/J. It would be helpful to mark all three enhancers on the plot on C, and to ensure the scale is accurate. This may be an issue with IGV viewer, not the authors.

2. Significance:

Significance (Required)

One of the challenges in understanding differential lymphatic gene regulation has been the lack of genomic resources. Consequently, the datasets contained within this paper are likely to be greatly appreciated by anyone studying this subject, both in the context of the vasculature and beyond.
Gene regulation within different types of endothelial cells is still relatively poorly understood, and the role of chromatin changes in this regulation is not established. In fact, I wish that the authors had discussed this aspect more: is it likely that changes in chromatin accessibility or organisation could explain all differential gene expression seen during lymphatic specification, and if not, what does? And how does this compare to other cell types?

3. How much time do you estimate the authors will need to complete the suggested revisions:

Estimated time to Complete Revisions (Required)

(Decision Recommendation)

Less than 1 month

4. *Review Commons* values the work of reviewers and encourages them to get credit for their work. Select 'Yes' below to register your reviewing activity at Web of Science Reviewer Recognition Service (formerly Publons); note that the content of your review will not be visible on Web of Science.

Web of Science Reviewer Recognition

No

Review #2

1. Evidence, reproducibility and clarity:

Evidence, reproducibility and clarity (Required)

In the study "Distinct topologically associated domains underlie regulatory logic in lymphatic endothelial cells contributing to proper cell differentiation," the authors investigate the cis-regulatory landscape that distinguishes lymphatic endothelial cells (LECs) from blood endothelial cells (BECs) in zebrafish, representing two closely related endothelial lineages that derived from common progenitors during vascular development. To uncover the regulatory mechanisms driving this lineage divergence, the authors combined fluorescence-activated cell sorting (FACS) with a multi-omics strategy, including Hi-C and ATAC-seq, as well as single-cell RNA sequencing. Using this approach, they identified cell-type-specific TADs, chromatin loops, and enhancer elements that likely underpin the distinct gene expression programs of LECs vs BECs. Beyond these structural differences, the authors observed broader shifts in the type of chromatin interactions (gene-gene, enhancer-gene, etc) between the two lineages, which could reflect differences in their differentiation states.
Their findings are functionally validated by testing the expression of candidate regulators of LEC specification and several tissue-specific enhancers using enhancer-reporter assays. Finally, by integrating transcription factor footprinting from open chromatin regions with single-cell transcriptomic data, the study reveals that distinct, context-dependent TF binding landscapes drive LEC and BEC gene regulatory programs.

The data well support the main conclusions; however, the manuscript requires some additional clarification regarding data analysis and experimental approach.

Major comments:

- The relationship between the mixed LEC and VEC population used for the scRNA-seq experiment and the isolated LECs and BECs analysed in the Hi-C and ATAC-seq experiments should be clarified. Explaining the use of different cell sorting strategies across these assays would help the reader understand the experimental design and ensure confidence in data integration.
In the manuscript, the importance of using consistent sorting strategies for ATAC-seq and Hi-C is emphasized. Given that a different sorting approach was used for scRNA-seq, it should be explicitly addressed whether comparability across datasets is still maintained. Additionally, clarification is needed on the relationship between VECs and BECs. If VECs are considered a subset of BECs, how might this affect the interpretation and integration of the datasets? Addressing or discussing this point will help assess data compatibility and the robustness of the conclusions from the analyses.
In the paragraph beginning with "We leverage the scRNA-sequencing data…," the authors refer to both "BECs and LECs" and "LEC- or VEC-specific" single-cell expression data. As mentioned before, the relationship between VECs and BECs should be clarified to enhance clarity and avoid potential confusion.
- The main sections describing the association of DA peaks to promoter, promoter-proximal sites, or short-range enhancers (primarily related to Figures 3 and 4) are not well structured and are, at times, confusing. A clearer explanation of the analytical criteria, particularly how DA peaks are assigned to genes, and more precise terminology would significantly improve this part of the manuscript.
First, the authors compare DA peaks at the promoters of the top 50 differentially expressed genes (DEGs), but find no strong correlation between promoter accessibility and gene expression levels (Figure S3F). However, it is unclear which genomic intervals were considered "promoters" for this analysis. Was a specific window (e.g., +/- 1 kb around the transcription start site) used to define promoter-associated peaks? Furthermore, it is unclear how this analysis differs from the one shown in Supplementary Figure S3K.
Second, the description in the section "Identification of candidate lymphatic regulators based on cell type-enriched gene expression and chromatin accessibility" is at times misleading. The analysis primarily focused on promoter-proximal regions. However, phrases such as "To assess in detail the differential chromatin accessibility across the whole locus…" suggest a broader investigation of the entire regulatory landscape of a gene, while the analysis is restricted to regions within 3 kb of promoters or transcription start sites. A better distinction would help to differentiate this section from the one that follows.
Third, the section "LEC chromatin landscape reveals accessible enhancers in the proximity to LEC-enriched genes" appears to shift the focus to distal DA peaks, but it is not clearly explained how this analysis differs from the previous one. Is the analysis limited to peaks

located more than 3 kb from the TSS? The inclusion of a -1.2 kb enhancer of cxcl12a suggests otherwise. Did the previously identified candidate gene loci also include DA peaks in non-coding regions? Additionally, the rationale for selecting the loci analysed in this section, as well as the choice of tested enhancers, should be explained.
Finally, the manuscript refers to certain elements as "short-range enhancers." However, it is not specified how these are defined. Did the authors apply a specific distance cutoff from the TSS to identify short-range enhancers? If not solely distance-based, what additional criteria were used to classify a regulatory element as short-range? A clearer definition of this term would help the reader better understand the results and the logic of the analysis.
- A rationale for the selection of candidate genes and enhancers should be provided: "We selected four genes for further characterisation: arrb2b, ptx3a, lgmn and stab1." However, it is not clear what criteria were applied for this selection. Did the number of DA ATAC-peaks play a role in the selected candidate genes?
Similarly, for the enhancers +461mafba, +47mafba, and -70mafba, the rationale for their selection should be provided. Figure panels 6C, G, and J show LEC-specific differential accessibility (DA) peaks, but it remains unclear why these enhancers were chosen over others nearby. The selection of the -70mafba enhancer seems understandable due to its isolated and distinct DA peak. However, the +47mafba region lies within a broader cluster of DA peaks, raising the question of why this specific element was prioritized. Was it selected based on peak intensity, proximity to transcription factor motifs, evolutionary conservation, or some other feature? Clarifying the basis for selecting these candidates, whether based on specific quantitative thresholds or functional relevance, would greatly strengthen the conclusions in this part.
- (Optional) The chromatin loop analysis could benefit from additional information regarding the overlap with CTCF binding sites at chromatin loops. Are common loops associated with CTCF motifs, and do tissue-specific loops operate independently of CTCF? While only CTCF binding data from whole zebrafish embryos are publicly available (GEO GSM5344493), could motif-based predictions be used to infer potential CTCF binding at loop ends? Such an analysis could help determine whether differential CTCF binding may underlie changes in TAD structure between LECs and BECs. For instance, could the LEC-specific TAD reorganisation, observed for example at the nr2f2 locus, be explained by tissue-specific CTCF occupancy? (I marked this comment as optional, but I strongly believe that a more extensive analysis of stable and tissue-specific chromatin interactions could significantly enhance the study and broaden its impact.)
- The manuscript states that the "+461mafba enhancer falls at the end of a LEC-specific loop, supporting direct chromatin interaction with the mafba promoter." Could the authors specify the window size (? kb) used to assign DA peaks or DEGs at loops? What distance from the loop start and end was considered when assigning regulatory features?

Additionally, there appears to be some inconsistency between the description and the data shown in the figures. In the main text, the authors state: "Out of these, tbx1 (Figure 5A-B), mafba (Figure 6B) and nr2f2 (Figure 5E-F) showed a clear interaction between the promoter and a putative distal regulatory element." However, the chromatin loop shown in Figure 5B does not appear to overlap with the +127kb tbx1 element. Likewise, the loop interaction involving +461kb mafba and its promoter in Figure 6C is not clear. Clarification is needed regarding how enhancers and promoters were linked to loop interaction. Was this based on overlap with loop, proximity to loop, or additional data?
Finally, it would be helpful if the authors described the method used to annotate genomic features (e.g., promoters, enhancers) at chromatin loops.
- Why are cell clusters other than LECs and VECs not annotated in the scRNA-seq dataset? An extended annotation by top marker genes and literature (optional) would strengthen the data analysis and increase the utility of the dataset as a resource.

Minor comments:

- Could the authors comment on whether the use of the gata2 promoter in the F0 enhancer assays may have biased the observed reporter activity, particularly in the vascular GFP expression domains that were scored?
- "...on average 300M number of chromatin contacts per sample..." Does this number refer to the total number of valid Hi-C read pairs? Please clarify in the text.
- The legend of Figure 1 refers to three biological replicates for both ATAC-seq and Hi-C, whereas the Hi-C description in the main text and Methods section mentions only two replicates. Please clarify the number of biological replicates
- The colour code legend in Figure 2A for LEC (green) and VEC (purple) at 5 dpf is switched at 7dpf. I believe the one at 5 dpf is correct. Please clarify.
- The expressions "The chromatin organisation shows rearrangements in loci of genes enriched in LECs and BECs" or "LEC-enriched loci undergo chromosomal rearrangements" may be confusing to the reader and, in the latter case, are inaccurate. The term chromosomal rearrangements typically refers to structural changes in the DNA sequence itself. To avoid misunderstanding, it would be more appropriate to refer to these observations as changes in chromatin conformation or reorganization of chromatin topology, or similar.
- In the manuscript, the term "closest locus" is used frequently. For clarity, the authors should be more specific, for example, by referring to the "closest gene" or "closest gene locus" when discussing genes.
- In the legend of Figure 2E, F, it states: "Differential TAD boundaries are plotted in red." However, I do not see any red markings in the figure. This requires clarification. Additionally,

the arrowheads indicate major changes in TAD boundaries associated with differential gene expression. However, the track labelled "Differential TAD boundaries" does not appear to include these boundaries. Please describe this track and clarify whether the arrowheads represent a subset of the differential TAD boundaries by adding their coordinates to the track.
- For Figure 3B, it would be helpful to indicate the identity of the LEC and BEC/VEC cell clusters for clarity.
- Regarding Figure 5: It should be considered to place the gene track and annotated enhancer regions directly below the Hi-C contact maps. Then indicate a zoom into the enhancer region (e.g., panel B of tbx1 locus). Alternatively, better describe zoom into the enhancer region. Additionally, the tbx1 locus includes another enhancer region (+13 kb tbx1), which may be confusing. Clarifying this in the figure or legend would be sufficient.
- "We identified LEC clusters at 5 and 7 dpf by the expression of known LEC genes..." However, plots in S2B show 8 clusters (5dpf) and 6 clusters (7dpf). Please clarify the discrepancy.
- I suggest indicating the sorted cell population used for scRNA-seq (double-positive) in Figure 2A, similar to what is shown in Figure 1A.
- Editing: In the introduction, "LEC progenitors across the embryo though its downstream components Mafba and Mafbb" should be corrected to: "...through...."
- Editing: "For nr2f2 we identified two enhances:" please change to "enhancers"
- Editing: "in zebrafish, but has been shown in mice *shown* to repress lymphatic fate"
- Editing: "SCC" likely refers to SSC, Side scatter. Please correct.
- Editing: Legend Figure 1F "All lever TADs", please correct.
- Editing: Parts of Figure 5 legend (lgmn enhancer) refer to Supplementary Figure 6.

Referee cross-commenting

Major comment 1: I believe that computational methods are likely better suited for detecting differential TAD organization than visual inspection. That said, I also share the reviewer's concern that changes in chromatin interactions are hard to assess "by eye," and the blue annotation lines may strongly influence perceptions of these differences. To address this, in addition to emphasizing the point in the Discussion, it might be beneficial to zoom in on the region of interest to illustrate the differences between LECs and BECs, particularly since the current genomic region in Figure 2 spans several megabases. This could be done in a supplementary figure. Optional: Alternatively, difference maps may be a useful tool for visualising these differences.

2. Significance:

Significance (Required)

The identification of tissue-specific chromatin interactions and the annotation of regulatory elements in animal models require carefully designed and precisely executed experimental approaches. The present manuscript elegantly identifies LEC- and BEC-specific chromatin interactions based on changes in TADs and chromatin loops in a genome-wide manner. Remarkably, the observed patterns are highly dynamic, even between these closely related endothelial lineages, underscoring that tissue-specific 3D genome organization is closely linked to lineage-specific gene regulation. The quality of the dataset is supported by the high rate of tested enhancers that show lineage-specific activity in vivo. This description of tissue-specific chromatin conformation in highly differentiated cells is relevant to the 3D genome field. Moreover, the integration of lineage-specific enhancer landscapes with a comprehensive atlas of endothelial cell types offers important insights into the gene regulatory logic underlying vascular development and its associated diseases.

Reviewer expertise (Keywords):

Gene regulation, 3D chromatin architecture, embryonic development, TADopathies, enhancers

3. How much time do you estimate the authors will need to complete the suggested revisions:

Estimated time to Complete Revisions (Required)

(Decision Recommendation)

Between 1 and 3 months

4. *Review Commons* values the work of reviewers and encourages them to get credit for their work. Select 'Yes' below to register your reviewing activity at [Web of Science Reviewer Recognition Service] (formerly Publons); note that the content of your review will not be visible on Web of Science.

Web of Science Reviewer Recognition

Yes

Revision Plan

Manuscript number: RC--2025-03079
Corresponding author(s): Katarzyna, Koltowska

1. General Statements [optional]

We would like to thank the reviewers for their insightful comments which has helped us significantly improve our manuscript and identify further experiments needed to strengthen our conclusions.
Please find the reviewers comments below in black and our responses in blue.

2. Description of the planned revisions

Reviewer 1

1. I do not doubt the bioinformatics underpinning the analysis in Figure 2, but the TAD organisational changes shown in 2E-F look slight, to the point that I would not have noted them without the blue lines. Given that e.g. prox1a is expressed in LECs only, it is hard to appreciate that such a minimal change in chromatin structure could lead to such extreme changes in expression. I was also surprised that the lower order TAD domains changed so little. This also appears true in Figure 5 although potentially to a lesser extent. I would have been interested to have had this (and the ATAC-seq data) discussed in a more agnostic manner - would this level of chromatin change have been expected, how does it compare to similar analysis in non-EC cell types (e.g. smooth muscle cells after activation), what does this say about gene regulation per se. As it is, I did not feel that the text fully connected with the images of the results.

Referee 2 cross-commenting

Major comment 1: I believe that computational methods are likely better suited for detecting differential TAD organization than visual inspection. That said, I also share the reviewer's concern that changes in chromatin interactions are hard to assess "by eye," and the blue annotation lines may strongly influence perceptions of these differences. To address this, in addition to emphasizing the point in the Discussion, it might be beneficial to zoom in on the region of interest to illustrate the differences between LECs and BECs, particularly since the current genomic region in Figure 2 spans several megabases. This could be done in a supplementary figure. Optional: Alternatively, difference maps may be a useful tool for visualising these differences.

As pointed out by Reviewer 2, computational methods are considered more valid than visual inspections to determine the presence of differences at the TAD levels. However, we agree with both reviewers that our visualization of HiC data can be significantly improved. We therefore

plan to explore different methods to represent the HiC matrices, in order to make the differences in TAD organization more visually evident.
We also agree with Reviewer 1 that at the moment the lower-level TADs are under-described in the text. We will therefore add a characterization of these structures in LECs and BECs.

5- It would be beneficial to the reader to show images of entire transient and stable transgenic zebrafish expressing the various enhancer:GFP constructs (potentially in the supplement).

We agree that whole-body images of the transient or stable transgenic lines are needed. Such imaging will be acquired and included in the final version of the paper.

Reviewer 2

4- (Optional) The chromatin loop analysis could benefit from additional information regarding the overlap with CTCF binding sites at chromatin loops. Are common loops associated with CTCF motifs, and do tissue-specific loops operate independently of CTCF? While only CTCF binding data from whole zebrafish embryos are publicly available (GEO GSM5344493), could motif-based predictions be used to infer potential CTCF binding at loop ends? Such an analysis could help determine whether differential CTCF binding may underlie changes in TAD structure between LECs and BECs. For instance, could the LEC-specific TAD reorganisation, observed for example at the nr2f2 locus, be explained by tissue-specific CTCF occupancy? (I marked this comment as optional, but I strongly believe that a more extensive analysis of stable and tissue-specific chromatin interactions could significantly enhance the study and broaden its impact.)

We agree with Reviewer 2 that an analysis of the predicted CTCF binding sites, as well as a comparison with available zebrafish datasets, would improve our manuscript. We therefore plan to run such analysis, leveraging the available data from Franke et al (2021) for comparison.

13- In the legend of Figure 2E, F, it states: "Differential TAD boundaries are plotted in red." However, I do not see any red markings in the figure. This requires clarification. Additionally, the arrowheads indicate major changes in TAD boundaries associated with differential gene expression. However, the track labelled "Differential TAD boundaries" does not appear to include these boundaries. Please describe this track and clarify whether the arrowheads represent a subset of the differential TAD boundaries by adding their coordinates to the track.

The boundaries are indeed plotted in red, however, we realized that due to the figure size they appear as black. The text has been corrected accordingly.

With regards to the differential TAD boundaries, we agree with Reviewer 2 that there is a discrepancy between out TADs and boundary predictions. We think this is because we used two different methods, OnTADs and TADcompare, to compute the two analyses. The two programs use different algorithms and work on different scales, which might explain why the results do not

align. During revision, we plan to try other methods to obtain better consistency between these two aspects of the dataset.

3. Description of the revisions that have already been incorporated in the transferred manuscript

We have indicated the changes in the main text by grey highlighting.

Reviewer 1

2- Related to Figure 2, is the Hi-C data able to establish if promoter contacts change significantly between LECs and BECs for genes that are differentially expressed? E.g. well-studied genes such as prox1a or mafba, which have defined lymphatic enhancers also?

We thank Reviewer 1 for suggesting the inclusion of these data.
For *both gene*, we have added a panel in Figure S6, showing the presence of tissue specific loops at the gene locus, and addressed these observations in the text: "*Both tissue-specific and common chromatin loops could be identified at the gene loci of known lymphatic regulators, such as prox1a (**Figure** **S6A**) and mafba (**Figure** **S6B**).".*
For *mafba*, a description of the interaction between the *+461mafba* enhancer and a LEC-specific chromatin loop is present in Figure 6. In addition, we have inserted a panel in Figure S6B showing the general chromatin organisation at the *mafba* locus and the presence of several tissue-specific and common loops in the region. This observation was addressed in the text: *"The complexity of mafba regulation, with multiple enhancers being capable of driving the expression in different vascular beds, suggests that coordinated enhancer activity may be critical for modulating lineage-specific expression. In addition, the presence of several chromatin loops, both common and tissue-specific, surrounding the mafba locus, further strengthen the idea that mafba regulation in the vasculature depends on complex mechanisms, involving several layers of chromatin organisation. In this context, it must be noted that several mafba candidate vascular enhancers remain untested, and likely contribute to this regulatory mechanism."*

3- It is unclear how much value can be placed on differential openness in identifying novel lymphatic genes (Figure 3D), since the analysis was limited to genes differentially expressed in LECs, and did not test the lymphatic-specificity of regions near DEG that were not differentially open (e.g. equally open in both cell types but near a lymphatic only gene). In fact, the data presented here seems to indicate that differential openness often has little correlation with gene expression. I think this is interesting (alongside the Hi-C data), not problematic. Within the endothelial field there are papers suggesting chromatin openness is and isn't associated with differential enhancer activity within different subtypes (e.g. Sissaoui et al 2020 suggests it is associated; Nornes et al 2025 suggests it is not), it would be interesting to discuss how the data here fits this discussion.

Reviewer 1 comment highlights two important aspects of our paper that were previously under-addressed:

1) Does chromatin accessibility in a locus correlate with gene expression level?
2) Is chromatin accessibility a good predictive tool to identify tissue-specific enhancers?

With regard to point 1, we agree with Reviewer 1 that our data suggests that chromatin state alone is a poor predictor of gene activity. We have expanded the result paragraph to more explicitly address this point: "*This indicates that chromatin accessibility alone is not a reliable indicator of gene activity, and that gene expression depends not only on chromatin state, but on the specific function of the accessible regions.*".

We also addressed our finding in the wider context of the available literature on the topic in the Discussion: *"Chromatin accessibility has long been used as a standard for predicting positive gene regulation (Klemm et al., 2019). However, in our data we did not observe a direct connection between chromatin accessibility and gene activity. Instead, open chromatin is associated with both over- and under-expressed genes in LECs vs VECs. On the other hand, the combination of gene expression data and tissue-specific chromatin accessibility peaks proved a reliable tool to identify vascular enhancers. In general, the predictive power of chromatin accessibility and other markers of enhancer activity in endothelial population has been a point of discussion, with some studies finding them a solid tool (Sissaoui et al., 2020), and other showing significant discrepancies between the in-silico prediction and the activity of tested elements (Nornes et al., 2025). In our case, the high rate of success for enhancer identification based on chromatin accessibility alone was likely helped by the relatively short range of the tested sequences, all under 70kb, and the high selectivity for strongly differential peaks. Overall, this observation confirms that while a link undoubtedly exists between chromatin accessibility and gene activity, the exact relation at a single locus level is often much more complex."*

4- Figure 4 utilizes the ATAC-seq dataset to identify novel enhancers. It would also be interesting to see the results around previously identified lymphatic enhancers in addition to the cdh6 enhancer (this also applies to later chromatin loop data). Previous work by the senior author identified a variety of lymphatic-specific enhancers for prox1a, would it be possible to clearly annotate within this paper the ATAC-seq/HiC/chromatin looping landscape around these validated enhancers?

We thank Reviewer 1 for this suggestion. We have added a panel to Figure S4 showing the open state in LECs of the previously described *prox1a* lymphatic enhancers, and addressed this in the text: *"Moreover, our previous work (Kazenwadel et al., 2023; Panara et al., 2024) described five prox1a enhancer active in different compartments of the lymphatics. Out of these, four (-84prox1a, -71prox1a, -14prox1a and -2.1prox1a) could also be identified as a peak of open chromatin in LECs (Figure S4C). The fifth lymphatic enhancer, +15.2prox1a, does not shown a clear signature of open chromatin, in accordance to what observed in the original*

publication. Overall, these observations validate the potential of the dataset for revealing tissue-specific cis-regulatory sequences."

6- The MAFBA binding motif is shared across the MAF family and is fairly broad, therefore I have significant reservations about the analysis in Figure 7. The text should clarify that analysis is on motifs, not directly assessing protein binding, and should explain why the focus is on MAFBA binding at promoters only. This part is also poorly validated, and does not really improve the paper.

7- I am unconvinced about the rational to look at Mafba/Tfe3a cooperation (the last part of the results), as this feels unconnected to the rest of the paper (e.g. there does not appear to be a link to chromatin occupation/motifs).

(Comments 6 and 7 are being addressed together).

We understand Reviewer 1 reservations around TF binding predictions, as indeed they can be quite unspecific. However, we consider the *in vivo* validation of the interaction between *mafba* and *tfe3* in Figure 7, and the vascular phenotype that is causes, as proof that important hints about regulatory novel interactions can be extracted from this analysis.

We have however better clarified in the text that the analysis was run on motifs, and not on bound proteins: "*To integrate these two aspects, we leveraged our identification of tissue-specific transcriptional signatures and chromatin organisation to explore the regulatory networks that establish and maintain the gene expression programs of LECs and BECs. To do so, we next explored the regulatory networks using our multi-omic datasets. Specifically we identified the transcription factor binding sites predicted to be located in the accessible chromatin regions. We then analysed the TF footprints in promoter ATAC peaks to predict the TF binding sites preferentially occupied in each cell type using the TOBIAS tool. (…) We identified promoters containing predicted occupied Mafba binding sites and compared them between LECs and BECs."*

We moreover addressed why we focused our network only on promoters: *"We focused on promoters as their attribution to a specific gene is more robust than for enhancers, which can often regulate distant loci."*

Finally, we agree that in the previous version of the manuscript the connection between Figure 7 and the rest of the manuscript was unclear. We have edited the text to better explain the logic connecting Figure 7 to the rest of the paper: *"In our analysis thus far, we have focused on the role of chromatin accessibility on cis-regulatory elements activity. An equally important aspect of gene regulation is mediated by transcription factors, which binds these elements and engage the transcriptional complex. To integrate these two aspects, we leveraged our identification of tissue-specific transcriptional signatures and chromatin organisation to explore the regulatory networks that establish and maintain the gene expression programs of LECs and BECs."*

8- It would be useful to more clearly discuss the number of differential consensus TADs between LECs and BECs in comparison with the number of differentially expressed genes between these two cell types. The discussion states that a large number of TADs show differences between LECs and BECs, but reading the paper this was not my impression. It

would therefore help to know what percentage of differentially regulated genes had clear TAD boundary differences when discussing this (I apologise if I missed this information).

We thank Reviewer 1 for suggesting this analysis. The percentage of consensus TADs in LECs and BECs is shown in Figure S1A-B, and is now addressed in the text: "*We found that LECs and BECs have a similar distribution and number of TADs at all hierarchical levels before and after filtering for consensus, with around 26% of TADs being specific for both LECs and BECs (**Figure S1A-B**).*"
The number and percentage of DEGs in LECs and BECs associated with a changing TAD boundary has also been added to the text, as well as a commentary on the significance of these observations: *"In total, we identified 54 LEC-enriched and 53 BEC-enriched genes associated with changing boundaries, corresponding respectively to 33.3% and 35.1% of all DE genes. This suggests that while not all differences in gene expression are associated with a change in TAD structure, these changes do play an important role in regulating tissue-specific gene expression."*
Note: the analysis in Figure 2D has been repeated in order to consider all DEG genes.

9- In general, there is a lot of different analyses here, and it would potentially be beneficial to remove some (e.g. TF motif search within characterised enhancers via TOBIAS, transcriptional signatures from ATAC-seq data) as it is not validated to the level required for publication yet neither is it essential for the paper and the underlying message.

We agree with Reviewer 1 that the predicted binding sites for the identified enhancers are superfluous, as we do not follow up on this topic, and therefore they have been removed from the paper. As for the TOBIAS analysis, we discussed why in our opinion it is coherent and important for the message of the paper at points 6 and 7 above.

10- I would have appreciated (in the Discussion) a more agnostic discussion of the chromatin changes between BEC and LECs - do all DEGs have differences in chromatin openness or organisation? Are all differentially open regions enhancers? Do some loci have differentially open chromatin but no detectable changes in TADs, and vice versa? And why? But

A paragraph discussing the significance of differences in chromatin accessibility and their relationship to enhancers has been added to the Discussion, as described in point 3.
As for the connection between changes in TADs and changes in chromatin accessibility, it must be noted that the two do not necessarily need to go hand in hand. The changes in TADs facilitate chromatin contacts and bring regulatory regions closer to their target gene, but such rearrangements do not necessarily imply changes in the open state of the underlying chromatin. Indeed, whether TAD boundaries are positively or negatively associated with accessible chromatin region is still under debate, with some studies supporting a negative correlation (Bediga et al, 2021), while other support a positive one (Chen et al, 2024). Overall, the nature and degree of correlation between TAD changes and chromatin accessibility is still unclear, and we think that addressing it goes beyond the scope of this manuscript.

11- In Figure 5, the locations of the enhancers on the HiC images are not clear.

We thank Reviewer 1 for pointing out this confusing detail. Arrowheads have been added to Figure 5 to show the position of the enhancers in the HiC maps

12- I am unsure of the utility of listing TFs with potential motifs within each enhancer, and believe this could be left out without impacting the paper. This type of prediction is very dependent on the program used and the settings used. If the authors prefer to keep this information, it should be annotated onto the sequence of the enhancers alongside the information provided by the prediction software (e.g. stringency, strength of fit to consensus).

As discussed above at point 9, we have decided to remove this analysis as we agree it does not provide much to the paper.

13- The expression pattern of the +24nr2f2 enhancer does not mimic nr2f2, this could be discussed?

We have added a paragraph commenting on the activity of both +127tbx1 and +24nr2f2 in unexpected endothelial populations*: "Noticeably, for both +24nr2f2 and +127tbx1, reporter activity was detected in endothelial population which are marked by low expression of the corresponding gene: for +127tbx1, in the veins (Figure 4A) and for nr2f2, the artery (DanioCell, Sur et al., 2023). This expanded domain of activity reflects the capacity of these DNA fragments to drive broader expression across endothelia, but at the endogenous gene locus, additional regulatory interactions probably constrain activity of these enhancers."*

14- The chromatin accessibility plots for mafba appear to vary in height, and in regions marked as DA, between Figure 6C and G/J. It would be helpful to mark all three enhancers on the plot on C, and to ensure the scale is accurate. This may be an issue with IGV viewer, not the authors.

This is indeed, as suggested by Reviewer 1, an issue with the visualization software, which "shortens" peaks as the zoom decreases. However, following Reviewer 2 suggestion, we have opted to use zoom-ins of the end of loops, which allowed us to show the peaks in their native size.

Reviewer 2

1a- The relationship between the mixed LEC and VEC population used for the scRNA-seq experiment and the isolated LECs and BECs analysed in the Hi-C and ATAC-seq experiments should be clarified. Explaining the use of different cell sorting strategies across these assays would help the reader understand the experimental design and ensure confidence in data integration. In the manuscript, the importance of using consistent sorting strategies for ATAC-

seq and Hi-C is emphasized. Given that a different sorting approach was used for scRNA-seq, it should be explicitly addressed whether comparability across datasets is still maintained. Additionally, clarification is needed on the relationship between VECs and BECs. If VECs are considered a subset of BECs, how might this affect the interpretation and integration of the datasets? Addressing or discussing this point will help assess data compatibility and the robustness of the conclusions from the analyses.

As the reviewer pointed out, we have utilized two different sorting strategies for HiC and ATAC sequencing vs scRNA sequencing. This is due to technical limitations regarding the number of cells needed for the techniques. At the time of the experiments, the technical request for HiC and ATAC sequencing was of 50.000 cells per repeat. The relative low signal of the *lyve1b* line did not allow us to sort enough cells within one replicate. We therefore decided to use the *TgBAC(prox1a:RFP) ;Tg(fli1a:nGFP)* line, which ensure we could proceed with sequencing, albeit at the cost of sorting a different population of non-lymphatic endothelial cells (all BEC instead of just veins).
In order to clarify the relationship between BECs and VECs, the following paragraph has been addressed in the text: "*To obtain a genome-wide understanding of which LEC-enriched gene loci undergo chromosomal rearrangements, we have characterised the transcriptional signature of LECs and venous endothelial cells (VECs), the subset of BECs more closely related to LECs, by single-cell (sc) RNA-sequencing. As LECs and VECs share several genes and developmental dynamics, we reasoned that the differences between them must largely recapitulate the general differences between LECs and the general BEC population.*"

1b- In the paragraph beginning with "We leverage the scRNA-sequencing data...," the authors refer to both "BECs and LECs" and "LEC- or VEC-specific" single-cell expression data. As mentioned before, the relationship between VECs and BECs should be clarified to enhance clarity and avoid potential confusion.

We thank the reviewer for pointing out this imprecision. We have edited the text to correctly indicate whether the results refer to BECs or VECs.

2a- The main sections describing the association of DA peaks to promoter, promoter-proximal sites, or short-range enhancers (primarily related to Figures 3 and 4) are not well structured and are, at times, confusing. A clearer explanation of the analytical criteria, particularly how DA peaks are assigned to genes, and more precise terminology would significantly improve this part of the manuscript.

We thank Reviewer 2 for pointing out the confusion in the text. A more detailed description of how peaks were annotated to genomic features has been added to the "*Bulk ATAC-sequencing data analysis*" section of the Methods. Moreover, the criteria for promoter identification have been added in the main text *"Using this data we identified the top 500 differentially accessible peaks (Figure S3D, Table S7) and the top 100 promoters (defined as regions up to 3 kb upstream of the TSS) (Figure S3E, Table S8)"*

2b- First, the authors compare DA peaks at the promoters of the top 50 differentially expressed genes (DEGs), but find no strong correlation between promoter accessibility and gene expression levels (Figure S3F). However, it is unclear which genomic intervals were considered "promoters" for this analysis. Was a specific window (e.g., +/- 1 kb around the transcription start site) used to define promoter-associated peaks? Furthermore, it is unclear how this analysis differs from the one shown in Supplementary Figure S3K.

A more thorough explanation of how peaks were assigned their identity has been added to the text as explained at point 2a, 2c and 5c.
With regard to panel S3K and the corresponding analysis, this is work that was done during the first draft of this paper. We agree with Reviewer 2 that, given the shape and scope of the manuscript, this analysis impedes the clarity of the current version of the paper without adding significant information. We have therefore opted to remove it.

2c- Second, the description in the section "Identification of candidate lymphatic regulators based on cell type-enriched gene expression and chromatin accessibility" is at times misleading. The analysis primarily focused on promoter-proximal regions. However, phrases such as "To assess in detail the differential chromatin accessibility across the whole locus..." suggest a broader investigation of the entire regulatory landscape of a gene, while the analysis is restricted to regions within 3 kb of promoters or transcription start sites. A better distinction would help to differentiate this section from the one that follows.

As previously stated (2a and 2b), we have removed the analysis at S3K and rewritten the explanation of how peaks were assigned their identity. With regard to the question of which DA regions were considered in the analysis in Figure 3B-D, we considered all DA regions without filtering for their assigned identity or position, as stated in the text "*we selected the top LEC-specific differentially accessible (DA) regions by average logarithmic fold change (logFC)*".

2d- Third, the section "LEC chromatin landscape reveals accessible enhancers in the proximity to LEC-enriched genes" appears to shift the focus to distal DA peaks, but it is not clearly explained how this analysis differs from the previous one. Is the analysis limited to peaks located more than 3 kb from the TSS? The inclusion of a -1.2 kb enhancer of cxcl12a suggests otherwise. Did the previously identified candidate gene loci also include DA peaks in non-coding regions? Additionally, the rationale for selecting the loci analysed in this section, as well as the choice of tested enhancers, should be explained.

We agree with Reviewer 2 that in the previous version of the manuscript it was not clear how this analysis differed from the previous one. We have rewritten the beginning of the paragraph to better highlight this difference: *"Gene expression is regulated through active enhancers marked by an open chromatin conformation. Therefore, in order to further validate the ATAC-sequencing database, we looked for potential LEC enhancers to be tested in vivo. Such candidates were defined as LEC-specific peaks of open chromatin in the non-coding regions*

surrounding genes enriched in the LEC clusters. Four of these putative enhancers were selected based on signal intensity, easy attribution to a gene and connection to endothelial cell biology (Figure 4A-B, 4D-E, 4G-H, 4J-K), and tested in vivo by cloning into the ZED vector (Bessa et al., 2009) (Figure 4).”

2e- Finally, the manuscript refers to certain elements as "short-range enhancers." However, it is not specified how these are defined. Did the authors apply a specific distance cutoff from the TSS to identify short-range enhancers? If not solely distance-based, what additional criteria were used to classify a regulatory element as short-range? A clearer definition of this term would help the reader better understand the results and the logic of the analysis.

We agree with Reviewer 2 that this nomenclature is confusing. This is indeed a left-over from previous versions of the manuscript, and we thank the reviewer for pointing it out, as it is quite confusing for a reader. Throughout the text, the definition has been changed to “ATAC-based enhancer” and “HiC-based enhancer”, as it better reflects the difference in methodology used to identify them.

3a- A rationale for the selection of candidate genes and enhancers should be provided:
"We selected four genes for further characterisation: arrb2b, ptx3a, lgmn and stab1." However, it is not clear what criteria were applied for this selection. Did the number of DA ATAC-peaks play a role in the selected candidate genes?

The rational for picking the ATAC-based enhancers to be tested is provided at point 2d.
For the selection of candidate genes, we have clarified in the text: *“We selected four genes for further characterisation: arrb2b, ptx3a, lgmn and stab1. Out of these, stab1 and ptx3a were selected due to their previously described expression in the lymphatic endothelium (Kzhyshkowska, 2006; Xiang et al., 2020). arrb2b and lgmn do not have a well-described role in the vasculature, and were therefore selected to test the potential for discovery of new lymphatic endothelial factors.”*

3b- Similarly, for the enhancers +461mafba, +47mafba, and -70mafba, the rationale for their selection should be provided. Figure panels 6C, G, and J show LEC-specific differential accessibility (DA) peaks, but it remains unclear why these enhancers were chosen over others nearby. The selection of the -70mafba enhancer seems understandable due to its isolated and distinct DA peak. However, the +47mafba region lies within a broader cluster of DA peaks, raising the question of why this specific element was prioritized. Was it selected based on peak intensity, proximity to transcription factor motifs, evolutionary conservation, or some other feature? Clarifying the basis for selecting these candidates, whether based on specific quantitative thresholds or functional relevance, would greatly strengthen the conclusions in this part.

We agree that the rationale was not sufficiently explained. The question posed by Reviewer 2 is now addressed in the text: *“To understand how this gene regulation is achieved in LECs and*

VECs, we have focused on identifying and characterising some of its vascular enhancers. Although no TAD rearrangements were detected in the mafba locus, we have selected three candidate sequences, +461mafba, +47mafba and -70mafba (Figure 6A-C, 6F, 6H), based on a combination of ATAC-sequencing and Hi-C. Specifically, +461mafba was picked based on the presence of a LEC-specific chromatin loop connecting it to the mafba gene, while +47mafba was selected at random among the numerous LEC-specific peaks of chromatin accessibility found in the large gene desert downstream of the mafba locus. Finally, -70mafba was selected in order to test if the region upstream of mafba also contained vascular enhancers."

We also further clarified that this analysis likely did not identify all the vascular *mafba* enhancers, but only a subset: "*The complexity of mafba regulation, with multiple enhancers being capable of driving the expression in different vascular beds, suggests that coordinated enhancer activity may be critical for modulating lineage-specific expression. (…) It must in addition be noted that several mafba candidate vascular enhancers remain untested, and likely contribute to this regulatory mechanism."*

5a- The manuscript states that the "+461mafba enhancer falls at the end of a LEC-specific loop, supporting direct chromatin interaction with the mafba promoter." Could the authors specify the window size (? kb) used to assign DA peaks or DEGs at loops? What distance from the loop start and end was considered when assigning regulatory features?

We agree with Reviewer 2 that this point was not sufficiently explained.

We have added in the main text in the "The genomic features connected by LEC- and BEC-specific chromatin loops differ" section the rational for assigning a DA peak to a loop end: *"An open chromatic region was assigned to the end of a loop when it fell within 10 kb of the loop matrix bin (25 kb)"*, and we refer back to it when necessary.

We moreover added a more detailed explanation of the methodology of loop calling in the "Chromatin loop analysis" section of methods "*Chromatin loops were identified by GPU HiCCUPS (juicer_tools version 2.20.00) using default parameters. Loops from merged calls from 5kb, 10kb and 25kb matrix resolutions (output file merged_loops.bedpe) files were used in further analyses. Loops shared between replicates (tissue consensus loops) within a distance of 25 kb were identified using compare (juicer_tools version 1.9.9)."*

5b- Additionally, there appears to be some inconsistency between the description and the data shown in the figures. In the main text, the authors state: "Out of these, tbx1 (Figure 5A-B), mafba (Figure 6B) and nr2f2 (Figure 5E-F) showed a clear interaction between the promoter and a putative distal regulatory element." However, the chromatin loop shown in Figure 5B does not appear to overlap with the +127kb tbx1 element. Likewise, the loop interaction involving +461kb mafba and its promoter in Figure 6C is not clear. Clarification is needed regarding how enhancers and promoters were linked to loop interaction. Was this based on overlap with loop, proximity to loop, or additional data?

We thank Reviewer 2 for pointing this out. As mentioned in point 5a, we have added a line explaining how accessible regions were assigned to loop ends. Upon further inspection, we also

found an annotation mistake in the figure, which reported the wrong position for *-64nr2f2*, much further away from the loop end that it is. The mistake has been corrected. However, it must be noted that there will always be a discrepancy between figures and data: graphically, the end of the loop is represented as a point, but in reality, it will always be a genomic region, which size depends on bin size and chosen approach.

5c- Finally, it would be helpful if the authors described the method used to annotate genomic features (e.g., promoters, enhancers) at chromatin loops.

We thank Reviewer 2 for spotting the missing Methods section. It has now been added: "*Regions of accessible chromatins were annotated to the end of a loop when they fell within 10kb of the loop matrix bin. Genomic features were assigned to the region of accessible chromatin as described in the "Bulk ATAC-sequencing data analysis" section, and used to select the loop-mediated interactions of interest."*

6- Why are cell clusters other than LECs and VECs not annotated in the scRNA-seq dataset? An extended annotation by top marker genes and literature (optional) would strengthen the data analysis and increase the utility of the dataset as a resource.

We agree with Reviewer 2 that fully annotating clusters is fundamental for correct scRNA analysis. However, the full characterization of the additional endothelial clusters in this scRNA dataset is part of a second paper currently under revision in Nature Communications.
Still, from Figure S2C-E it can be seen that all the clusters are indeed endothelial, as shown by the ubiquitous expression of *fli1a* and the presence of other vascular markers. In addition, we have clarified in the text that for this study we are considering only the main LEC and VEC cluster respectively: *"We identified the main LEC cluster at 5 and 7 dpf by the expression of known LEC genes, such as prox1a, mafba and cdh6 (Figure 2A, S2C-F, Table S4-5). The main VEC cluster was determined by the expression of known genes enriched in BECs, such as cdh5 and kdrl (Figure 2A, S2C-F, Table S4-5)."*

7- Could the authors comment on whether the use of the gata2 promoter in the F0 enhancer assays may have biased the observed reporter activity, particularly in the vascular GFP expression domains that were scored?

The presence of a *gata2* promoter in the ZED vector is indeed a valid source of worry. However, in a previous publication (Grimm et al, 2023) we have tested the empty ZED vector extensively and shown that although this tool does indeed drive unspecific reporter expression in several tissue, including neuronal and dorsal root ganglion expression, it does not drive unspecific expression in lymphatic endothelial structures.

8- "...on average 300M number of chromatin contacts per sample..." Does this number refer to the total number of valid Hi-C read pairs? Please clarify in the text.

We have added a clarification on which reads were considered in the main text: "*Sequencing of 50000 cells per sample from two replicates identified on average 300M number of chromatin contacts per sample (read pairs after filtering duplicates, intra-fragment pairs, low quality alignments).*"

We also better addressed the methodology used the Method section: *"The QC metrics and heatmaps of Hi-C contacts are derived from data containing reads aligned with MAPQ ≥ 30, after removal of duplicates and intra-fragment pairs."*

9- The legend of Figure 1 refers to three biological replicates for both ATAC-seq and Hi-C, whereas the Hi-C description in the main text and Methods section mentions only two replicates. Please clarify the number of biological replicates

We thank Reviewer 2 for spotting this imprecision. The number of repeats for the HiC experiment is indeed 2. This detail has been corrected throughout the text.

14- For Figure 3B, it would be helpful to indicate the identity of the LEC and BEC/VEC cell clusters for clarity.

The legend has been changed to indicate that the clusters represented in the heatmap correspond to the main LEC and VEC clusters: *"Heatmap showing expression of genes associated with the top DA ATAC-seq peaks at 5 dpf in the main LEC and VEC clusters described in Figure 2A. Cells were ordered by their assigned clusters. Colour scale represents log-normalised read counts."*

15- Regarding Figure 5: It should be considered to place the gene track and annotated enhancer regions directly below the Hi-C contact maps. Then indicate a zoom into the enhancer region (e.g., panel B of tbx1 locus). Alternatively, better describe zoom into the enhancer region. Additionally, the tbx1 locus includes another enhancer region (+13 kb tbx1), which may be confusing. Clarifying this in the figure or legend would be sufficient.

We agree that the previous version of the figure was not sufficiently clear. As suggested, we have added zoom-ins of the regions at the end of the loops, to better show which elements are interacting.
The position of the *+13tbx1* enhancer, described in Figure 4, has been marked with a grey arrow in order to clarify its identity and position.

16- "We identified LEC clusters at 5 and 7 dpf by the expression of known LEC genes..." However, plots in S2B show 8 clusters (5dpf) and 6 clusters (7dpf). Please clarify the discrepancy.

This question was addressed as part of point 6.

17- I suggest indicating the sorted cell population used for scRNA-seq (double-positive) in Figure 2A, similar to what is shown in Figure 1A.

An arrow has been added to indicate that the double- positive population is the one that was sorted for this experiment.

Finally, we would like to thank Reviewer 2 for bringing to our attention a number of imprecisions and mistakes in our text and figures. The manuscript has been adjusted accordingly to the comments listed below:

10- The colour code legend in Figure 2A for LEC (green) and VEC (purple) at 5 dpf is switched at 7 dpf. I believe the one at 5 dpf is correct. Please clarify
11- The expressions "The chromatin organisation shows rearrangements in loci of genes enriched in LECs and BECs" or "LEC-enriched loci undergo chromosomal rearrangements" may be confusing to the reader and, in the latter case, are inaccurate. The term chromosomal rearrangements typically refers to structural changes in the DNA sequence itself. To avoid misunderstanding, it would be more appropriate to refer to these observations as changes in chromatin conformation or reorganization of chromatin topology, or similar.
12- In the manuscript, the term "closest locus" is used frequently. For clarity, the authors should be more specific, for example, by referring to the "closest gene" or "closest gene locus" when discussing genes.
- Editing: In the introduction, "LEC progenitors across the embryo though its downstream components Mafba and Mafbb" should be corrected to: "...through...."
- Editing: "For nr2f2 we identified two enhances:" please change to "enhancers"
- Editing: "in zebrafish, but has been shown in mice *shown* to repress lymphatic fate"
- Editing: "SCC" likely refers to SSC, Side scatter. Please correct.
- Editing: Legend Figure 1F "All lever TADs", please correct.
- Editing: Parts of Figure 5 legend (Igmn enhancer) refer to Supplementary Figure 6.

4. Description of analyses that authors prefer not to carry out

N/A

Dear Dr. Koltowska

Thank you for the transfer of your research manuscript to our journal. I have now read and discussed your work with my colleagues here and assessed the accompanying referee reports from Review Commons. In addition, I have also consulted an expert editorial advisor, who is familiar both with the field and with our journal and its scope.

The Review Commons referees, as well as our scientific advisor, express interest in the work's value for the vascular endothelial field.

Given this support and given that you plan to address all referee concerns and to further strengthen your data, we would like to invite you to revise your manuscript in line with your submitted revision plan. Please address all referee concerns in a complete point-by-point response. Acceptance of the manuscript will depend on a positive outcome of a second round of review. It is EMBO Reports policy to allow a single round of revision only and acceptance or rejection of the manuscript will therefore depend on the completeness of your responses included in the next, final version of the manuscript.

In particular, we would like to emphasize point 7 below: the revised manuscript should include a data availability statement with a link that resolves directly to the dataset and should give reviewer access. In addition, we feel that it will be important for the readers to understand the annotation of the scRNA-seq dataset, so please adjust the text to clarify the relationship with the parallel Nature Communications submission. Citations across the two manuscripts will need to be harmonized prior to publication.

We realize that it is difficult to revise to a specific deadline. In the interest of protecting the conceptual advance provided by the work, we recommend a revision within 3 months (February 1st). Please discuss the revision progress ahead of this time with the editor if you require more time to complete the revisions.

I am also happy to discuss the revision further via e-mail or a video call, if you wish.

You can either publish the study as a short report or as a full article. For short reports, the revised manuscript should not exceed 27,000 characters (including spaces but excluding materials & methods and references) and 5 main plus 5 expanded view figures. The results and discussion sections must further be combined, which will help to shorten the manuscript text by eliminating some redundancy that is inevitable when discussing the same experiments twice. For a normal article there are no length limitations, but it should have more than 5 main figures and the results and discussion sections must be separate. In both cases, the entire materials and methods must be included in the main manuscript file.

================
IMPORTANT NOTE:

We perform an initial quality control of all revised manuscripts before re-review. Your manuscript will FAIL this control and the handling will be delayed IN CASE the following APPLIES:

1) A data availability section providing access to data deposited in public databases is missing. If you have not deposited any data, please add a sentence to the data availability section that explains that.

2) Your manuscript contains statistics and error bars based on n=2. Please use scatter blots in these cases. No statistics should be calculated if n=2.

When submitting your revised manuscript, please carefully review the instructions that follow below. Failure to include requested items will delay the evaluation of your revision.

================

When submitting your revised manuscript, we will require:

1) a .docx formatted version of the manuscript text (including legends for main figures, EV figures and tables). Please make sure that the changes are highlighted to be clearly visible.

2) individual production quality figure files as .eps, .tif, .jpg (one file per figure).
Please download our Figure Preparation Guidelines (figure preparation pdf) from our Author Guidelines pages https://www.embopress.org/page/journal/14693178/authorguide for more info on how to prepare your figures.

3) a .docx formatted letter INCLUDING the reviewers' reports and your detailed point-by-point responses to their comments. As

part of the EMBO Press transparent editorial process, the point-by-point response is part of the Review Process File (RPF), which will be published alongside your paper.

4) a complete author checklist, which you can download from our author guidelines (). Please insert information in the checklist that is also reflected in the manuscript. The completed author checklist will also be part of the RPF.

5) Please note that all corresponding authors are required to supply an ORCID ID for their name upon submission of a revised manuscript (). Please find instructions on how to link your ORCID ID to your account in our manuscript tracking system in our Author guidelines
()

6) We replaced Supplementary Information with Expanded View (EV) Figures and Tables that are collapsible/expandable online. A maximum of 5 EV Figures can be typeset. EV Figures should be cited as 'Figure EV1, Figure EV2" etc... in the text and their respective legends should be included in the main text after the legends of regular figures.

- For the figures that you do NOT wish to display as Expanded View figures, they should be bundled together with their legends in a single PDF file called *Appendix*, which should start with a short Table of Content. Appendix figures should be referred to in the main text as: "Appendix Figure S1, Appendix Figure S2" etc. See detailed instructions regarding expanded view here:

- Additional Tables/Datasets should be labeled and referred to as Table EV1, Dataset EV1, etc. Legends have to be provided in a separate tab in case of .xls files. Alternatively, the legend can be supplied as a separate text file (README) and zipped together with the Table/Dataset file.

7) Before submitting your revision, primary datasets (and computer code, where appropriate) produced in this study need to be deposited in an appropriate public database (see <
https://www.embopress.org/page/journal/14693178/authorguide#dataavailability>).

Please remember to provide a reviewer password if the datasets are not yet public.

The accession numbers and database should be listed in a formal "Data Availability " section (placed after Materials & Method) that follows the model below (see also < https://www.embopress.org/page/journal/14693178/authorguide#dataavailability>). Please note that the Data Availability Section is restricted to new primary data that are part of this study.

Data availability

The datasets (and computer code) produced in this study are available in the following databases:

- RNA-Seq data: Gene Expression Omnibus GSE46843 (https://www.ncbi.nlm.nih.gov/geo/query/acc.cgi?acc=GSE46843)
- [data type]: [name of the resource] [accession number/identifier/doi] ([URL or identifiers.org/DATABASE:ACCESSION])

*** Note - All links should resolve to a page where the data can be accessed. ***

8) At EMBO Press we ask authors to provide source data for the main manuscript figures. You will receive a separate email with instructions for providing source data with your revised manuscript, including how to upload and organize the files.

Additional information on source data and instruction on how to label the files are available

9) The journal requires a statement specifying whether or not authors have competing interests (defined as all potential or actual interests that could be perceived to influence the presentation or interpretation of an article). In case of competing interests, this must be specified in your disclosure statement. Further information: https://www.embopress.org/competing-interests

10) Figure legends and data quantification:
The following points must be specified in each figure legend:

- the name of the statistical test used to generate error bars and P values,
- the EXACT p-values,
- the number (n) of independent experiments (please specify technical or biological replicates) underlying each data point,
- the nature of the bars and error bars (s.d., s.e.m.)

- If the data are obtained from n {less than or equal to} 5, show the individual data points in addition to the SD or SEM.
- If the data are obtained from n {less than or equal to} 2, use scatter blots showing the individual data points.

Discussion of statistical methodology can be reported in the materials and methods section, but figure legends should contain a

basic description of n, P and the test applied.

See also the guidelines for figure legend preparation:
https://www.embopress.org/page/journal/14693178/authorguide#figureformat

- Please also include scale bars in all microscopy images and define their size in the legend, not the image.

11) Our journal encourages inclusion of *data citations in the reference list* to directly cite datasets that were re-used and obtained from public databases. Data citations in the article text are distinct from normal bibliographical citations and should directly link to the database records from which the data can be accessed. In the main text, data citations are formatted as follows: "Data ref: Smith et al, 2001" or "Data ref: NCBI Sequence Read Archive PRJNA342805, 2017". In the Reference list, data citations must be labeled with "[DATASET]". A data reference must provide the database name, accession number/identifiers and a resolvable link to the landing page from which the data can be accessed at the end of the reference. Further instructions are available at .

12) All Materials and Methods need to be described in the main text using our 'Structured Methods' format. According to this format, the Methods section includes a Reagents and Tools Table (listing key reagents, experimental models, software and relevant equipment and including their sources and relevant identifiers) followed by a Methods and Protocols section describing the methods, ideally using a step-by-step protocol format. The aim is to facilitate adoption of the methodologies across labs. Please download and fill our Reagents and Tools Table template (.docx), which you can find in our author guidelines: https://www.embopress.org/page/journal/14693178/authorguide#structuredmethods.
When submitting your revised manuscript, please do not include the Reagents and Tools Table in the Methods section of the manuscript but upload it as a separate file choosing the file type "Reagent Table".
An example of a Method paper with Structured Methods can be found here:
https://www.embopress.org/doi/10.15252/msb.20178071.

13) As part of the EMBO publication's Transparent Editorial Process, EMBO Reports publishes online a Review Process File to accompany accepted manuscripts. This File will be published in conjunction with your paper and will include the referee reports, your point-by-point response and all pertinent correspondence relating to the manuscript.

You are able to opt out of this by letting the editorial office know (emboreports@embo.org). If you do opt out, the Review Process File link will point to the following statement: "No Review Process File is available with this article, as the authors have chosen not to make the review process public in this case."

We would also welcome the submission of cover suggestions, or motifs to be used by our Graphics Illustrator in designing a cover.

I look forward to seeing a revised form of your manuscript when it is ready and please let me know if you have questions or comments regarding the revision.

Yours sincerely,

Kurt Weir
Editor
EMBO Reports

We would like to thank the reviewers for positive comments and suggestions that improved the quality of our work. Our detail responses to comments are below, we have indicated in the text the changes made in grey. Figure numbers have been updated throughout the document to reflect the current figure organisation.

Reviewer 1

1. I do not doubt the bioinformatics underpinning the analysis in Figure 2, but the TAD organisational changes shown in 2E-F look slight, to the point that I would not have noted them without the blue lines. Given that e.g. prox1a is expressed in LECs only, it is hard to appreciate that such a minimal change in chromatin structure could lead to such extreme changes in expression. I was also surprised that the lower order TAD domains changed so little. This also appears true in Figure 5 although potentially to a lesser extent. I would have been interested to have had this (and the ATAC-seq data) discussed in a more agnostic manner - would this level of chromatin change have been expected, how does it compare to similar analysis in non-EC cell types (e.g. smooth muscle cells after activation), what does this say about gene regulation per se. As it is, I did not feel that the text fully connected with the images of the results.

Referee 2 cross-commenting

Major comment 1: I believe that computational methods are likely better suited for detecting differential TAD organization than visual inspection. That said, I also share the reviewer's concern that changes in chromatin interactions are hard to assess "by eye," and the blue annotation lines may strongly influence perceptions of these differences. To address this, in addition to emphasizing the point in the Discussion, it might be beneficial to zoom in on the region of interest to illustrate the differences between LECs and BECs, particularly since the current genomic region in Figure 2 spans several megabases. This could be done in a supplementary figure. Optional: Alternatively, difference maps may be a useful tool for visualising these differences.

As pointed out by Reviewer 2, computational methods are considered more valid than visual inspections to determine the presence of differences at the TAD levels. However, we agree with both reviewers that our visualization of HiC data can be significantly improved. We have therefore used an alternative way to visualise the difference in megamaps between LECs and BECs, which can be seen for example in **Figure 2C-D, 5C, 5G** and supplementary **Figure EV4A**. This visualisation is based on a normalised log2(LEC/BEC) signal, and it reflects LEC enriched HiC signal in green and BEC enriched signal in purple. We think that these plots allow better appreciation of the differences in TAD organisation. We also computationally indicated with lines the shift in the different level of TADs in LECs and BECs supplementary Figure 4A.
We also agreed that a more agnostic characterisation of the ATAC signal was needed. Therefore, we investigated the association of accessible chromatin with differential TADs and differentially expressed genes. We found that LEC-specific peaks of open chromatin annotated to genes enriched in LECs are significantly associated with differential TADs, suggesting that changes in TAD structure may affect chromatin accessibility and gene expression associated with LEC identity. We address this new analysis in the text and in

Figure EV3G: *We therefore investigated whether regions of open chromatin are preferentially associated with the previously identified differential TADs. We found that LEC DA were more accessible in differential TADs (p=0.0000047, Chi-squared test). We further investigated whether ATAC peaks preferentially accessible in LEC and associated with differential TADs can be linked to altered gene expression using a permutation test (n=5000) (Figure EV3G). We found that while no positive association could be found between BEC DA regions and differential TADs, regardless of gene expression, LEC DA regions annotated to LEC DE genes show a significant association with differential TADs. Therefore, while a generally there is no strong association between differential TAD boundaries and DE genes (Figure EV2G), LEC-specific genes tend to be associated with differential TAD boundaries, suggesting that changes in TAD structure may affect chromatin accessibility and gene expression associated with LEC identity.*
We also agree with Reviewer 1 that at in the previous version the lower-level TADs are under-described in the text. We have therefore explored the association of these TADs with known site of CTCF occupancy. These analyses are discussed under Comment 4 from Reviewer 2.

5- It would be beneficial to the reader to show images of entire transient and stable transgenic zebrafish expressing the various enhancer:GFP constructs (potentially in the supplement).

We have added whole-body images for all the tested enhancers (transient or stable transgenic lines), they can be found in Figure A2.

Reviewer 2

4- (Optional) The chromatin loop analysis could benefit from additional information regarding the overlap with CTCF binding sites at chromatin loops. Are common loops associated with CTCF motifs, and do tissue-specific loops operate independently of CTCF? While only CTCF binding data from whole zebrafish embryos are publicly available (GEO GSM5344493), could motif-based predictions be used to infer potential CTCF binding at loop ends? Such an analysis could help determine whether differential CTCF binding may underlie changes in TAD structure between LECs and BECs. For instance, could the LEC-specific TAD reorganisation, observed for example at the nr2f2 locus, be explained by tissue-specific CTCF occupancy? (I marked this comment as optional, but I strongly believe that a more extensive analysis of stable and tissue-specific chromatin interactions could significantly enhance the study and broaden its impact.)

We agree with Reviewer 2 that an analysis of the predicted CTCF binding sites, as well as a comparison with available zebrafish datasets, would improve our manuscript. We added such an analysis, leveraging the available data from Franke et al (2021) for comparison. We have found that the boundaries of consensus TADs show a strong association with CTCF occupancy regardless of hierarchical level (Figure 1G). However, the boundaries of deeper level differential TADs are not significantly associated with peaks of CTCF occupancy at 48hpf. This might suggest that these tissue-specific TADs emerge later during development, accompanying the acquisition of mature LEC and BEC identities. These observations are addressed in the text: *We then asked whether published CTCF ChIP-seq peaks are associated with common and differential TAD boundaries in LECs and BECs to underscore*

their structural relevance. To do so, we leveraged the previously published dataset of CTCF occupancy in whole embryos at 48 hpf (Franke et al., 2021). The associations between consensus and differential TAD boundaries and regions of CTCF occupancy were evaluated using permutation tests. Boundaries were stratified into three groups: level 1, including the outermost TADs; levels 2 and 3, including intermediate inner TADs; and level 4, including TADs deeper in the TAD hierarchy, of level 4 or higher. As expected, the boundaries of consensus TADs show a strong association with CTCF occupancy regardless of hierarchical level (Figure 1G). However, we observed that the boundaries of deeper level differential TADs are not significantly associated with peaks of CTCF occupancy at 48hpf. This might suggest that these tissue-specific TADs emerge later during development, accompanying the acquisition of mature LEC and BEC identities.

13- In the legend of Figure 2E, F, it states: "Differential TAD boundaries are plotted in red." However, I do not see any red markings in the figure. This requires clarification. Additionally, the arrowheads indicate major changes in TAD boundaries associated with differential gene expression. However, the track labelled "Differential TAD boundaries" does not appear to include these boundaries. Please describe this track and clarify whether the arrowheads represent a subset of the differential TAD boundaries by adding their coordinates to the track.

The boundaries are indeed plotted in red, however, we realized that due to the figure size they appear as black. The text has been corrected accordingly.

With regards to the differential TAD boundaries, we agree with Reviewer 2 that in the previous manuscript there was a large discrepancy between out TADs and boundary predictions. We think this is because we used two different methods, OnTADs and TADcompare, to compute the two analyses. We have therefore opted to repeat the TADcompare boundary scoring using three window sizes in order to better capture the variety of TAD size we observe in our data. Although this approach has improved the consistency of the data, it ultimately has to be observed that the two methods are based on fundamentally different mathematical approaches, and that full consistency cannot be expected. However, we do believe that while this analysis might miss some of the finer differences in TAD organisation, it does capture the most prominent changes between chromatin organisation in LECs and BECs

---

The following comments were already addressed upon the transferer of the manuscript to EMBO Reports.

Reviewer 1

2- Related to Figure 2, is the Hi-C data able to establish if promoter contacts change significantly between LECs and BECs for genes that are differentially expressed? E.g. well-studied genes such as prox1a or mafba, which have defined lymphatic enhancers also?

We thank Reviewer 1 for suggesting the inclusion of these data.

For both genes, we have added a panel in Figure EV4, showing the presence of tissue specific loops at the gene locus, and addressed these observations in the text: *Both tissue specific and common chromatin loops could be identified at the gene loci of known lymphatic regulators, such as prox1a (Figure EV4A) and mafba (Figure EV4B).*
For *mafba*, a description of the interaction between the *+461mafba* enhancer and a LEC-specific chromatin loop is present in Figure 6. In addition, we have inserted a panel in Figure EV4B showing the general chromatin organisation at the *mafba* locus and the presence of several tissue-specific and common loops in the region. This observation was addressed in the text: *The complexity of mafba regulation, with multiple enhancers being capable of driving the expression in different vascular beds, suggests that coordinated enhancer activity may be critical for modulating lineage-specific expression. In addition, the presence of several chromatin loops, both common and tissue-specific, surrounding the mafba locus (Figure EV4B) further strengthen the idea that mafba regulation in the vasculature depends on complex mechanisms, involving several layers of chromatin organisation. In this context, it must be noted that several mafba candidate vascular enhancers remain untested, and likely contribute to this regulatory mechanism.*

3- It is unclear how much value can be placed on differential openness in identifying novel lymphatic genes (Figure 3D), since the analysis was limited to genes differentially expressed in LECs, and did not test the lymphatic-specificity of regions near DEG that were not differentially open (e.g. equally open in both cell types but near a lymphatic only gene). In fact, the data presented here seems to indicate that differential openness often has little correlation with gene expression. I think this is interesting (alongside the Hi-C data), not problematic. Within the endothelial field there are papers suggesting chromatin openness is and isn't associated with differential enhancer activity within different subtypes (e.g. Sissaoui et al 2020 suggests it is associated; Nornes et al 2025 suggests it is not), it would be interesting to discuss how the data here fits this discussion.

Reviewer 1 comment highlights two important aspects of our paper that were previously under-addressed:

1) Does chromatin accessibility in a locus correlate with gene expression level?
2) Is chromatin accessibility a good predictive tool to identify tissue-specific enhancers?

With regard to point 1, we agree with Reviewer 1 that our data suggests that chromatin state alone is a poor predictor of gene activity. We have expanded the result paragraph to more explicitly address this point: *This indicates that change in chromatin accessibility alone is not a reliable indicator of altered gene activity*.

We also addressed our finding in the wider context of the available literature on the topic in the Discussion: *Chromatin accessibility has long been used as a standard for predicting positive gene regulation (Klemm et al., 2019). However, in our data we did not observe a direct connection between chromatin accessibility and gene activity. Instead, open chromatin is associated with both over- and under-expressed genes in LECs vs VECs. On the other hand, the combination of gene expression data and tissue-specific chromatin accessibility peaks proved a reliable tool to identify vascular enhancers. In general, the predictive power of chromatin accessibility and other markers of enhancer activity in endothelial population has been a point of discussion, with some studies finding them a solid tool (Sissaoui et al., 2020), and other showing significant discrepancies between the in silico prediction and the*

activity of tested elements (Nornes et al., 2025). In our case, the high rate of success for enhancer identification based on chromatin accessibility alone was likely helped by the relatively short range of the tested sequences, all under 70kb, and the high selectivity for strongly differential peaks. Overall, this observation confirms that while a link undoubtedly exists between chromatin accessibility and gene activity, the exact relation at a single locus level is often much more complex.

4- Figure 4 utilizes the ATAC-seq dataset to identify novel enhancers. It would also be interesting to see the results around previously identified lymphatic enhancers in addition to the cdh6 enhancer (this also applies to later chromatin loop data). Previous work by the senior author identified a variety of lymphatic-specific enhancers for prox1a, would it be possible to clearly annotate within this paper the ATAC-seq/HiC/chromatin looping landscape around these validated enhancers?

We thank Reviewer 1 for this suggestion. We have added a panel to Figure A1 showing the open state in LECs of the previously described *prox1a* lymphatic enhancers, and addressed this in the text: *Moreover, our previous work (Kazenwadel et al., 2023; Panara et al., 2024) described five prox1a enhancers active in different compartments of the lymphatics. Out of these, four (-84prox1a, -71prox1a, -14prox1a and -2.1prox1a) could also be identified as a peak of open chromatin in LECs (Figure A1G). The fifth lymphatic enhancer, +15.2prox1a, did not show a clear signature of open chromatin, in accordance with the original publication. Overall, these observations validate the potential of the dataset for revealing tissue-specific cis-regulatory sequences.*

6- The MAFBA binding motif is shared across the MAF family and is fairly broad, therefore I have significant reservations about the analysis in Figure 7. The text should clarify that analysis is on motifs, not directly assessing protein binding, and should explain why the focus is on MAFBA binding at promoters only. This part is also poorly validated, and does not really improve the paper.
7- I am unconvinced about the rational to look at Mafba/Tfe3a cooperation (the last part of the results), as this feels unconnected to the rest of the paper (e.g. there does not appear to be a link to chromatin occupation/motifs).

(Comments 6 and 7 are being addressed together).
We understand Reviewer 1 reservations around TF binding predictions, as indeed they can be quite unspecific. However, we consider the *in vivo* validation of the interaction between *mafba* and *tfe3* in Figure 7, and the vascular phenotype that is causes, as proof that important hints about regulatory novel interactions can be extracted from this analysis.
We have however better clarified in the text that the analysis was run on motifs, and not on bound proteins: *we leveraged our identification of tissue-specific transcriptional signatures and chromatin organisation to explore the regulatory networks that establish and maintain the gene expression programs of LECs and BECs. To do so, we first identified the transcription factor binding sites which are predicted to be bound in the accessible chromatin regions. We analysed the TF footprints in promoter ATAC peaks to predict the TF binding sites preferentially occupied in each cell type using the TOBIAS tool (…) We identified promoters containing predicted occupied Mafba binding sites and compared them between LECs and BECs.*

We moreover addressed why we focused our network only on promoters: *Only promoters were considered for this analysis as their attribution to a specific gene is more robust that for enhancers, which can often regulate distant loci.*
Finally, we agree that in the previous version of the manuscript the connection between Figure 7 and the rest of the manuscript was unclear. We have edited the text to better explain the logic connecting Figure 7 to the rest of the paper: *In our analysis this far, we have focused on the role of chromatin accessibility on cis-regulatory elements activity. However, and equally important aspect of gene regulation is mediated by the transcription factors that binds to such elements and mediate the interaction with the transcriptional complex. In order to integrate these two aspects, we leveraged our identification of tissue-specific transcriptional signatures and chromatin organisation to explore the regulatory networks that establish and maintain the gene expression programs of LECs and BECs.*

8- It would be useful to more clearly discuss the number of differential consensus TADs between LECs and BECs in comparison with the number of differentially expressed genes between these two cell types. The discussion states that a large number of TADs show differences between LECs and BECs, but reading the paper this was not my impression. It would therefore help to know what percentage of differentially regulated genes had clear TAD boundary differences when discussing this (I apologise if I missed this information).

We thank Reviewer 1 for suggesting this analysis. The percentage of consensus TADs in LECs and BECs is shown in Figure EV1A-B, and is now addressed in the text: *We found that LECs and BECs have a similar distribution and number of TADs at all hierarchical levels before and after filtering for consensus, with around 25% of TADs being specific for both LECs and BECs (Figure EV1A-B).*
The number and percentage of DEGs in LECs and BECs associated with a changing TAD boundary has also been added to the text, as well as a commentary on the significance of these observations: *In total, we identified 46 LEC-enriched and 39 BEC-enriched genes associated with changing boundaries, corresponding respectively to 29% and 26% of all DE genes. This suggests that while not all differences in gene expression are associated with a change in TAD structure, these changes play an important role in regulating tissue-specific gene expression.*
Note: the analysis in Figure 2B has been repeated in order to consider all DEG genes.

9- In general, there is a lot of different analyses here, and it would potentially be beneficial to remove some (e.g. TF motif search within characterised enhancers via TOBIAS, transcriptional signatures from ATAC-seq data) as it is not validated to the level required for publication yet neither is it essential for the paper and the underlying message.

We agree with Reviewer 1 that the predicted binding sites for the identified enhancers are superfluous, as we do not follow up on this topic, and therefore they have been removed from the paper. As for the TOBIAS analysis, we discussed why in our opinion it is coherent and important for the message of the paper at points 6 and 7 above.

10- I would have appreciated (in the Discussion) a more agnostic discussion of the chromatin changes between BEC and LECs - do all DEGs have differences in chromatin openness or organisation? Are all differentially open regions enhancers? Do some loci have

differentially open chromatin but no detectable changes in TADs, and vice versa? And why? But

A paragraph discussing the significance of differences in chromatin accessibility and their relationship to enhancers has been added to the Discussion, as described in point 3.
As for the connection between changes in TADs and changes in chromatin accessibility, it must be noted that the two don't necessarily need to go hand in hand. The changes in TADs facilitate chromatin contacts and bring regulatory regions closer to their target gene, but such rearrangements don't necessarily imply changes in the open state of the underlying chromatin.
Indeed, whether TAD boundaries are positively or negatively associated with accessible chromatin region is still under debate, with some studies supporting a negative correlation (Bediga et al, 2021), while other support a positive one (Chen et al, 2024). Overall, the nature and degree of correlation between TAD changes and chromatin accessibility is still unclear, and we think that addressing it goes beyond the scope of this manuscript.

11- In Figure 5, the locations of the enhancers on the HiC images are not clear.

We thank Reviewer 1 for pointing out this confusing detail. Arrowheads have been added to Figure 5 to show the position of the enhancers in the HiC maps

12- I am unsure of the utility of listing TFs with potential motifs within each enhancer, and believe this could be left out without impacting the paper. This type of prediction is very dependent on the program used and the settings used. If the authors prefer to keep this information, it should be annotated onto the sequence of the enhancers alongside the information provided by the prediction software (e.g. stringency, strength of fit to consensus).

As discussed above at point 9, we have decided to remove this analysis as we agree it does not provide much to the paper.

13- The expression pattern of the +24nr2f2 enhancer does not mimic nr2f2, this could be discussed?

We have added a paragraph commenting on the activity of both *+127tbx1* and *+24nr2f2* in unexpected endothelial populations*: Noticeably, for both +24nr2f2 and +127tbx1, reporter activity was detected in endothelial population which are marked by low expression of the corresponding gene: for +127tbx1, in the veins (Figure 4A) and for nr2f2, the artery (DanioCell, Sur et al., 2023). This expanded domain of activity likely reflects the presence of additional regulatory interactions at the endogenous gene locus, which inhibit the expression in these populations.*

14- The chromatin accessibility plots for mafba appear to vary in height, and in regions marked as DA, between Figure 6C and G/J. It would be helpful to mark all three enhancers on the plot on C, and to ensure the scale is accurate. This may be an issue with IGV viewer, not the authors.

This is indeed, as suggested by Reviewer 1, an issue with the visualization software, which "shortens" peaks as the zoom decreases. However, following Reviewer 2 suggestion, we

have opted to use zoom-ins of the end of loops, which allowed us to show the peaks in their native size.

Reviewer 2

1a- The relationship between the mixed LEC and VEC population used for the scRNA-seq experiment and the isolated LECs and BECs analysed in the Hi-C and ATAC-seq experiments should be clarified. Explaining the use of different cell sorting strategies across these assays would help the reader understand the experimental design and ensure confidence in data integration. In the manuscript, the importance of using consistent sorting strategies for ATAC-seq and Hi-C is emphasized. Given that a different sorting approach was used for scRNA-seq, it should be explicitly addressed whether comparability across datasets is still maintained. Additionally, clarification is needed on the relationship between VECs and BECs. If VECs are considered a subset of BECs, how might this affect the interpretation and integration of the datasets? Addressing or discussing this point will help assess data compatibility and the robustness of the conclusions from the analyses.

As the reviewer pointed out, we have utilized two different sorting strategies for HiC and ATAC sequencing vs scRNA sequencing. This is due to technical limitations regarding the number of cells needed for the techniques. At the time of the experiments, the technical request for HiC and ATAC sequencing was of 50.000 cells per repeat. The relative low signal of the *lyve1b* line did not allow us to sort enough cells within one replicate. We therefore decided to use the *TgBAC(prox1a:RFP) ;Tg(fli1a:nGFP)* line, which ensure we could proceed with sequencing, albeit at the cost of sorting a different population of non-lymphatic endothelial cells (all BEC instead of just veins).
In order to clarify the relationship between BECs and VECs, the following paragraph has been addressed in the text: *To obtain a genome-wide understanding of which LEC-enriched gene loci undergo changes in chromatin conformation, we have characterised the transcriptional signature of LECs and venous endothelial cells (VECs), the subset of BECs more closely related to LECs, by single-cell (sc) RNA-sequencing. As LECs and VECs share several genes and developmental dynamics, we reasoned that the differences between them must largely recapitulate the general differences between LECs and the general BEC population.*

1b- In the paragraph beginning with "We leverage the scRNA-sequencing data...," the authors refer to both "BECs and LECs" and "LEC- or VEC-specific" single-cell expression data. As mentioned before, the relationship between VECs and BECs should be clarified to enhance clarity and avoid potential confusion.

We thank the reviewer for pointing out this imprecision. We have edited the text to correctly indicate whether the results refer to BECs or VECs.

2a- The main sections describing the association of DA peaks to promoter, promoter-proximal sites, or short-range enhancers (primarily related to Figures 3 and 4) are not well structured and are, at times, confusing. A clearer explanation of the analytical criteria, particularly how DA peaks are assigned to genes, and more precise terminology would significantly improve this part of the manuscript.

We thank Reviewer 2 for pointing out the confusion in the text. A more detailed description of how peaks were annotated to genomic features has been added to the "*Bulk ATAC-sequencing data analysis*" section of the Methods. Moreover, the criteria for promoter identification have been added in the main text: *Using this data we identified the top 500 differentially accessible peaks (Figure EV3D, Table S7) and the top 100 promoters (defined as regions up to 3 kb upstream of the TSS) (Figure EV3E, Table S8), showing the presence of differences in the chromatin accessibility between LECs and BECs.*

2b- First, the authors compare DA peaks at the promoters of the top 50 differentially expressed genes (DEGs), but find no strong correlation between promoter accessibility and gene expression levels (Figure S3F). However, it is unclear which genomic intervals were considered "promoters" for this analysis. Was a specific window (e.g., +/- 1 kb around the transcription start site) used to define promoter-associated peaks? Furthermore, it is unclear how this analysis differs from the one shown in Supplementary Figure S3K.

A more thorough explanation of how peaks were assigned their identity has been added to the text as explained at point 2a, 2c and 5c.
With regard to panel S3K and the corresponding analysis, this is work that was done during the first draft of this paper. We agree with Reviewer 2 that, given the shape and scope of the manuscript, this analysis impedes the clarity of the current version of the paper without adding significant information. We have therefore opted to remove it.

2c- Second, the description in the section "Identification of candidate lymphatic regulators based on cell type-enriched gene expression and chromatin accessibility" is at times misleading. The analysis primarily focused on promoter-proximal regions. However, phrases such as "To assess in detail the differential chromatin accessibility across the whole locus..." suggest a broader investigation of the entire regulatory landscape of a gene, while the analysis is restricted to regions within 3 kb of promoters or transcription start sites. A better distinction would help to differentiate this section from the one that follows.

As previously stated (2a and 2b), we have removed the analysis at S3K and rewritten the explanation of how peaks were assigned their identity. With regard to the question of which DA regions were considered in the analysis in Figure 3, we considered all DA regions without filtering for their assigned identity or position, as stated in the text: *we selected the top LEC-specific differentially accessible (DA) regions by average logarithmic fold change (logFC).*

2d- Third, the section "LEC chromatin landscape reveals accessible enhancers in the proximity to LEC-enriched genes" appears to shift the focus to distal DA peaks, but it is not clearly explained how this analysis differs from the previous one. Is the analysis limited to peaks located more than 3 kb from the TSS? The inclusion of a -1.2 kb enhancer of cxcl12a suggests otherwise. Did the previously identified candidate gene loci also include DA peaks in non-coding regions? Additionally, the rationale for selecting the loci analysed in this section, as well as the choice of tested enhancers, should be explained.

We agree with Reviewer 2 that in the previous version of the manuscript it was not clear how this analysis differed from the previous one. We have rewritten the beginning of the paragraph to better highlight this difference: *Gene expression is regulated through active*

enhancers marked by an open chromatin conformation. To further explore the ATAC-sequencing results, we looked for potential LEC enhancers to be tested in vivo. Such candidates were defined as LEC-specific peaks of open chromatin in the non-coding regions surrounding genes enriched in the LEC clusters. Four of these putative enhancers were selected by visual inspection based on signal intensity and easy attribution to a gene (Figure 4A-B, 4D-E, 4G-H, 4J-K), and tested in vivo by cloning into the ZED vector (Bessa et al., 2009) (Figure 4, A2B).

2e- Finally, the manuscript refers to certain elements as "short-range enhancers." However, it is not specified how these are defined. Did the authors apply a specific distance cutoff from the TSS to identify short-range enhancers? If not solely distance-based, what additional criteria were used to classify a regulatory element as short-range? A clearer definition of this term would help the reader better understand the results and the logic of the analysis.

We agree with Reviewer 2 that this nomenclature is confusing. This is indeed a left-over from previous versions of the manuscript, and we thank the reviewer for pointing it out, as it is quite confusing for a reader. Throughout the text, the definition has been changed to “ATAC-based enhancer” and “HiC-based enhancer”, as it better reflects the difference in methodology used to identify them.

3a- A rationale for the selection of candidate genes and enhancers should be provided: "We selected four genes for further characterisation: arrb2b, ptx3a, lgmn and stab1." However, it is not clear what criteria were applied for this selection. Did the number of DA ATAC-peaks play a role in the selected candidate genes?

The rational for picking the ATAC-based enhancers to be tested is provided at point 2d.
For the selection of candidate genes, we have clarified in the text: *We selected four genes for further characterisation: arrb2b, ptx3a, lgmn and stab1. Out of these, stab1 and ptx3a were selected due to their previously described expression in the lymphatic endothelium in other models (Kzhyshkowska, 2006; Petkova et al., 2023; Xiang et al., 2020). arrb2b and lgmn do not have a well-described role in the vasculature, and were therefore selected to test the potential for discovery of new endothelial factors.*

3b- Similarly, for the enhancers +461mafba, +47mafba, and -70mafba, the rationale for their selection should be provided. Figure panels 6C, G, and J show LEC-specific differential accessibility (DA) peaks, but it remains unclear why these enhancers were chosen over others nearby. The selection of the -70mafba enhancer seems understandable due to its isolated and distinct DA peak. However, the +47mafba region lies within a broader cluster of DA peaks, raising the question of why this specific element was prioritized. Was it selected based on peak intensity, proximity to transcription factor motifs, evolutionary conservation, or some other feature? Clarifying the basis for selecting these candidates, whether based on specific quantitative thresholds or functional relevance, would greatly strengthen the conclusions in this part.

We agree that the rationale was not sufficiently explained. The question posed by Reviewer 2 is now addressed in the text: *To understand how this gene regulation is achieved in LECs and VECs, we have focused on identifying and characterising some of its vascular enhancers. Although no TAD structural changes were detected in the mafba locus, we have*

selected three candidate sequences, +461mafba, +47mafba and -70mafba (Figure 6A-C, 6E, 6G), based on a combination of ATAC-sequencing and Hi-C. Specifically, +461mafba was picked based on the presence of a LEC-specific chromatin loop connecting it to the mafba gene, while +47mafba was selected at random among the numerous LEC-specific peaks of chromatin accessibility found in the large gene desert downstream of the mafba locus. Finally, -70mafba was selected in order to test if the shorter region upstream of mafba also contained vascular enhancers.

We also further clarified that this analysis likely did not identify all the vascular *mafba* enhancers, but only a subset: *The complexity of mafba regulation, with multiple enhancers being capable of driving the expression in different vascular beds, suggests that coordinated enhancer activity may be critical for modulating lineage-specific expression. In addition, the presence of several chromatin loops, both common and tissue-specific, surrounding the mafba locus (Figure EV4B) further strengthen the idea that mafba regulation in the vasculature depends on complex mechanisms, involving several layers of chromatin organisation. In this context, it must be noted that several mafba candidate vascular enhancers remain untested, and likely contribute to this regulatory mechanism.*

5a- The manuscript states that the "+461mafba enhancer falls at the end of a LEC-specific loop, supporting direct chromatin interaction with the mafba promoter." Could the authors specify the window size (? kb) used to assign DA peaks or DEGs at loops? What distance from the loop start and end was considered when assigning regulatory features?

We agree with Reviewer 2 that this point was not sufficiently explained.

We have added in the main text in the "The genomic features connected by LEC- and BEC-specific chromatin loops differ" section the rational for assigning a DA peak to a loop end: *An accessible chromatin region was assigned to the end of a loop when it fell within 10 kb of the loop matrix bin (25 kb)*, and we refer back to it when necessary.

We moreover added a more detailed explanation of the methodology of loop calling in the "Chromatin loop analysis" section of methods: *Chromatin loops were identified by GPU HiCCUPS (juicer_tools version 2.20.00) using default parameters. Loops from merged calls from 5kb, 10kb and 25kb matrix resolutions (output file merged_loops.bedpe) files were used in further analyses. Loops shared between replicates (tissue consensus loops) within a distance of 25 kb were identified using compare (juicer_tools version 1.9.9).*

5b- Additionally, there appears to be some inconsistency between the description and the data shown in the figures. In the main text, the authors state: "Out of these, tbx1 (Figure 5A-B), mafba (Figure 6B) and nr2f2 (Figure 5E-F) showed a clear interaction between the promoter and a putative distal regulatory element." However, the chromatin loop shown in Figure 5B does not appear to overlap with the +127kb tbx1 element. Likewise, the loop interaction involving +461kb mafba and its promoter in Figure 6C is not clear. Clarification is needed regarding how enhancers and promoters were linked to loop interaction. Was this based on overlap with loop, proximity to loop, or additional data?

We thank Reviewer 2 for pointing this out. As mentioned in point 5a, we have added a line explaining how accessible regions were assigned to loop ends. Upon further inspection, we also found an annotation mistake in the figure, which reported the wrong position for *-64nr2f2*, much further away from the loop end that it is. The mistake has been corrected. However, it must be noted that there will always be a discrepancy between figures and data:

graphically, the end of the loop is represented as a point, but in reality, it will always be a genomic region, which size depends on bin size and chosen approach.

5c- Finally, it would be helpful if the authors described the method used to annotate genomic features (e.g., promoters, enhancers) at chromatin loops.

We thank Reviewer 2 for spotting the missing Methods section. It has now been added: *Regions of accessible chromatin were annotated to the end of a loop when they fell within 10kb of the loop matrix bin. Genomic features were assigned to the region of accessible chromatin as described in the "Bulk ATAC-sequencing data analysis" section, and used to select the loop-mediated interactions of interest.*

6- Why are cell clusters other than LECs and VECs not annotated in the scRNA-seq dataset? An extended annotation by top marker genes and literature (optional) would strengthen the data analysis and increase the utility of the dataset as a resource.

We agree with Reviewer 2 that fully annotating clusters is fundamental for correct scRNA analysis. However, the full characterization of the additional endothelial clusters in this scRNA dataset is part of a second paper currently in preprint (https://doi.org/10.21203/rs.3.rs-5333191/v1).
Still, from Figure EV2C-E it can be seen that all the clusters are indeed endothelial, as shown by the ubiquitous expression of *fli1a* and the presence of other vascular markers. In addition, we have clarified in the text that for this study we are considering only the main LEC and VEC cluster respectively: *We identified the main LEC cluster at 5 and 7 dpf by the expression of known LEC genes, such as prox1a, mafba and cdh6 (Figure 2A, EV2C-F, Table S4-5). The main VEC cluster was determined by the expression of known genes enriched in BECs, such as cdh5 and kdrl (Figure 2A, EV2C-F, Table S4-5).*

8- "...on average 300M number of chromatin contacts per sample..." Does this number refer to the total number of valid Hi-C read pairs? Please clarify in the text.

We have added a clarification on which reads were considered in the main text: *Sequencing of 50000 cells per sample from two replicates identified on average 300M number of chromatin contacts per sample (read pairs after filtering duplicates, intra-fragment pairs, low quality alignments).*

We also better addressed the methodology used the Method section: *The QC metrics and matrices of Hi-C contacts are derived from data containing reads aligned with MAPQ ≥ 30, after removal of duplicates and intra-fragment pairs.*

9- The legend of Figure 1 refers to three biological replicates for both ATAC-seq and Hi-C, whereas the Hi-C description in the main text and Methods section mentions only two replicates. Please clarify the number of biological replicates

We thank Reviewer 2 for spotting this imprecision. The number of repeats for the HiC experiment is indeed 2. This detail has been corrected throughout the text.

14- For Figure 3B, it would be helpful to indicate the identity of the LEC and BEC/VEC cell clusters for clarity.

The legend has been changed to indicate that the clusters represented in the heatmap correspond to the main LEC and VEC clusters: *Heatmap of expression of genes associated with the top DA ATAC-seq peaks at 5 dpf in the main LEC and VEC clusters (Figure 2A). Cells are ordered by clusters. Colour indicates log-normalized read counts.*

15- Regarding Figure 5: It should be considered to place the gene track and annotated enhancer regions directly below the Hi-C contact maps. Then indicate a zoom into the enhancer region (e.g., panel B of tbx1 locus). Alternatively, better describe zoom into the enhancer region. Additionally, the tbx1 locus includes another enhancer region (+13 kb tbx1), which may be confusing. Clarifying this in the figure or legend would be sufficient.

We agree that the previous version of the figure was not sufficiently clear. As suggested, we have added zoom-ins of the regions at the end of the loops, to better show which elements are interacting.
The position of the *+13tbx1* enhancer, described in Figure 4, has been marked with a grey arrow in order to clarify its identity and position.

16- "We identified LEC clusters at 5 and 7 dpf by the expression of known LEC genes..." However, plots in S2B show 8 clusters (5dpf) and 6 clusters (7dpf). Please clarify the discrepancy.

This question was addressed as part of point 6.

17- I suggest indicating the sorted cell population used for scRNA-seq (double-positive) in Figure 2A, similar to what is shown in Figure 1A.

An arrow has been added to indicate that the double- positive population is the one that was sorted for this experiment.

Finally, we would like to thank Reviewer 2 for bringing to our attention a number of imprecisions and mistakes in our text and figures. The manuscript has been adjusted accordingly to the comments listed below:

10- The colour code legend in Figure 2A for LEC (green) and VEC (purple) at 5 dpf is switched at 7 dpf. I believe the one at 5 dpf is correct. Please clarify
11- The expressions "The chromatin organisation shows rearrangements in loci of genes enriched in LECs and BECs" or "LEC-enriched loci undergo chromosomal rearrangements" may be confusing to the reader and, in the latter case, are inaccurate. The term chromosomal rearrangements typically refers to structural changes in the DNA sequence itself. To avoid misunderstanding, it would be more appropriate to refer to these observations as changes in chromatin conformation or reorganization of chromatin topology, or similar.
12- In the manuscript, the term "closest locus" is used frequently. For clarity, the authors should be more specific, for example, by referring to the "closest gene" or "closest gene locus" when discussing genes.

- Editing: In the introduction, "LEC progenitors across the embryo though its downstream components Mafba and Mafbb" should be corrected to: "...through...."
- Editing: "For nr2f2 we identified two enhances:" please change to "enhancers"
- Editing: "in zebrafish, but has been shown in mice *shown* to repress lymphatic fate"
- Editing: "SCC" likely refers to SSC, Side scatter. Please correct.
- Editing: Legend Figure 1F "All lever TADs", please correct.
- Editing: Parts of Figure 5 legend (lgmn enhancer) refer to Supplementary Figure 6.

Reviewer 2

7- Could the authors comment on whether the use of the gata2 promoter in the F0 enhancer assays may have biased the observed reporter activity, particularly in the vascular GFP expression domains that were scored?

The presence of a *gata2* promoter in the ZED vector is indeed a valid source of worry. However, in a previous publication (Grimm et al, 2023) we have tested the empty ZED vector extensively and shown that although this tool does indeed drive unspecific reporter expression in several tissue, including neuronal and dorsal root ganglion expression, it doesn't drive unspecific expression in lymphatic endothelial structures.

1st Revision - Editorial Decision 22nd May 2026

Dear Dr. Koltowska

Thank you for the submission of your revised manuscript to EMBO reports. We have now received the full set of referee reports that is copied below.

As you will see, all referees are very positive about the study and request only minor changes to clarify text and figures.

From the editorial side, there are also a few things that we need before we can proceed with the official acceptance of your study:

- Please place the manuscript sections in the following order: Title page - Abstract & Keywords - Introduction - Results - Discussion - Methods - Data Availability - Acknowledgments - Disclosure Statement & Competing Interests - References - Figure Legends - (Main Tables with legends if applicable) - Expanded View Figure Legends.

- Please rename the "Resource Availability" section to Data Availability.

- Please rename the "Statement of conflicting interests" to Disclosure and Competing Interests Statement.

- Please remove the Reagents and Tools Table from the manuscript and upload separately.

- Please note that we have a 100 character limit (including spaces) for titles and 175 words for abstract, which should be in present tense. Please revise your title and abstract to fit our requirements.

- Please ensure that the publicly deposited datasets are available by publication.

- Please expand the detail on animal husbandry conditions in the Methods section.

- Please adjust the format of the reference list and of the in-text citations according to EMBO Journal format (up to 10 author names in the reference list before et al, DOIs should only be used for preprints and datasets that have not been published yet). Please refer to our Guide to Authors for additional information on EMBO J reference format.

- Please include a separate reference for publicly available datasets that have been reanalyzed in this manuscript.
Please refer to our detailed online guidelines for further information:
https://www.embopress.org/page/journal/14602075/authorguide#referencesformat

- Please rename the supplementary tables to Dataset EV1-Dataset EV23 and upload each separately. The nomenclature should be corrected in all places including the source file names, titles in the system, legend in each file, callouts in the manuscript.

- Please add a title page to the Appendix file with the title (Appendix to [Title]) and a Table of Contents listing each item and its page number. Please update the nomenclature to Appendix Figure S1, etc. from Appendix Figure A1, etc. in all places.

- Please note that EMBO press papers are accompanied online by:
A) a short (2 sentences) summary of the findings and their significance,
B) 2-5 short bullet points highlighting the key results, and
C) a synopsis image in .jpg or .png format that is exactly 550 pixels wide and 300 pixels high. Please note that the text needs to be legible at the final size. Please upload this information along with your revised manuscript (the text for A and B should be provided in one separate Word file uploaded as Cover Art/Synopsis Image).

- Thanks for providing the source data (SD). Please upload the SD file folders, which are currently all combined in a single ZIP archive, as one separate archive per each main figure. Source data files need to be saved in a scheme one figure/folder and then uploaded as .zip files. E.g. all the Source data files for figure 1 need to be saved in a single folder and this needs to be zipped and then uploaded as "SD figure 1.zip" file. For appendix figures, ZIP together all source data and rename to Appendix Fig. S1, etc.

- Our production/data editors have asked you to clarify several points in the figure legends (see below). Please incorporate these changes in the manuscript and return the revised file with tracked changes with your final manuscript submission.
- Please indicate the statistical test used for data analysis in the legends of figures 2D, 7D, E
- Please note that information related to n is missing in the legends of figures 3A, 7D, E
- Please note that the error bars are not defined in the legends of figures 7D, E.
- Please note that the arrow heads are not defined in the legends of figures 6E, H. This needs to be rectified.

We look forward to seeing a final version of your manuscript as soon as possible.

Kurt Weir
Editor
EMBO Reports

Referee #1:

All my initial comments have been satisfactorily dealt with. Below are a few specific suggestions of alterations within the text to clarify meaning:

1. Introduction sentence :"By in vivo validation, we have identified active ATAC- and HiC-based enhancer sequences driving LEC-confined reporter expression". This doesn't reflect the research described, needs rephrasing.

2. Results page 7 sentence: "In total about 2500 TADs have been identified solely in LECs and BECs and 4429 were common to the two cell populations. Out of these, differential TAD boundaries were found in association with 272 LEC TADs, 236 BEC TADs and 592 common TADs (Figure 1F)." This is confusingly worded about a key point of the paper. Looking at the relevant figure, I think it can be tightened up as: "In total, both LECs and BECs contain around 2500 TADs that were unique to their subtype, while 4429 were common to both cell populations. Out of these, differential TAD boundaries were found in association with 272 LEC-only TADs, 236 BEC-only TADs and 592 common TADs (Figure 1F)."

3. In the same section as above, they switch between defining shared TADs as "common" and as "consensus". This should be consistent, and clarified more clearly in the text. "Common" would seem like a better definition.

4. First sentence of page 8 in the next subsection, "..of which LEC-enriched gene loci undergo changes" should be changed to "of which genes with LEC-enriched expression undergo changes in loci chromatin confirmation" or similar (a loci cannot simply be enriched). The second sentence, starting " As LECs and VECs share several genes and developmental dynamics..." could also be clarified: as it stands, why does their shared genes/dynamics mean that their differences must recapitulate those between LECs and general blood ECs??

5. Figure 3D and text describing it: At 2dpf tail images of in situ, gene expression appears to be shown in the vein, with no evidence that expression is in the "lymphatic endothelium". The later timed images, particularly in the Appendix, are convincing. However, could the language describing this be tightened up in legend and text.

6. Figure 4: In Figure 4 B E H K, could the direction of the gene be more clearly labelled please? And in K, could the tested enhancer be marked by arrowhead as in others?

6b. Related appendix A1G. Some of the Prox1 enhancers appear open in both BECs and LECs but this is hard to tell. Please indicate Prox1 enhancer location on the BEC track, and specifically mention this in the text (since the other LEC enhancers discussed contained LEC-specific peaks.

Referee #2:

I would like to thank the authors for their thorough point-by-point response. My previous questions and concerns have been fully addressed. I also agree to the changes introduced in the revised manuscript, which improve both the readability and potential significance of the study.

I have only a few minor points and suggestions:

1. The authors might consider using "topologically associating domains" throughout the manuscript and title, as this is a more commonly used term in the literature.

2. The numbers reported in the manuscript text related to Figure 1E do not appear to match those shown in the figure: "...of which 151 were classified as 'complex' changes, 707 as 'merged/split', 40 as 'shifted', and 217 as 'strength' changes or unclassified (Figure 1E, Table 3)."

3. Related to the new Figure 1G, the terminology could be improved. Are "consensus" and "all" synonymous? Does "all" include differential boundaries? In addition, the figure legend refers to "all boundaries," whereas the main text refers to "boundaries of consensus TADs." More consistent terminology would improve clarity, particularly since TADs and TAD boundaries are distinct concepts, as the authors discuss.

4. I appreciate the effort and improvements made to visualize differences in TAD organization between LECs and BECs. In

Figures 2C and 2D, non-differential LEC TADs are shown in black and differential LEC TADs in orange. The manuscript text refers to the inclusion of a 150 kb region for the tbx1 gene locus and to a large ~1100 kb TAD encompassing prox1a in LECs. However, when looking at the differential TADs in the figure, it is difficult to identify which specific regions are referenced in the text. Two possible suggestions:
a) Indicate the relevant regions described in the main text within the figure panels.
b) Alternatively, consider using the green/purple colour scheme that is used throughout the manuscript for differential TADs (currently shown in orange), which may help distinguish gains or losses of TADs in LECs relative to BECs more intuitively.

5. Perhaps I overlooked it, but could the authors provide the list of genes referred to in the following sentence as supplementary information? "In total, we identified 46 LEC-enriched and 39 BEC-enriched genes associated with changing boundaries, corresponding respectively to 29% and 26% of all DE genes."

Typos:

"Therefore, while a generally there is no ..." remove "a"

"... chromatin accessibility are also accompanied by changes in TAD between LECs and BECs ..." change "TAD" to "TADs"

"Only promoters were considered for this analysis as their attribution to a specific gene is more robust that for enhancers ..." change to "more robust than that for enhancers"

Correct "Germline GFP+ enhancer" in Supplementary Table 22

Figure 2 legend: contains previous figure labels "E" and "F"

Figure 2 legend: missing period in "in orange Differential"

We thank the editor and referees for the positive and constructive feedback on our manuscript. We have integrated their comments and suggestions into the work as follows:

Editor requests:

With regards to the following point, we think the editorial team might have been referring an older version of the figures, as the panel numbers does not match the current content of the figures. Nevertheless, we double-checked that all our figure legends reference, when appropriate, the statistical test used, the n, and the definition of the error bars.

- Our production/data editors have asked you to clarify several points in the figure legends (see below). Please incorporate these changes in the manuscript and return the revised file with tracked changes with your final manuscript submission.
- Please indicate the statistical test used for data analysis in the legends of figures 2D, 7D, E
- Please note that information related to n is missing in the legends of figures 3A, 7D, E
- Please note that the error bars are not defined in the legends of figures 7D, E.
- Please note that the arrow heads are not defined in the legends of figures 6E, H. This needs to be rectified.

The following points have been fulfilled as requested:

- Please place the manuscript sections in the following order: Title page - Abstract & Keywords - Introduction - Results - Discussion - Methods - Data Availability - Acknowledgments - Disclosure Statement & Competing Interests - References - Figure Legends - (Main Tables with legends if applicable) - Expanded View Figure Legends.

- Please rename the "Resource Availability" section to Data Availability.

- Please rename the "Statement of conflicting interests" to Disclosure and Competing Interests Statement.

- Please remove the Reagents and Tools Table from the manuscript and upload separately.

- Please note that we have a 100 character limit (including spaces) for titles and 175 words for abstract, which should be in present tense. Please revise your title and abstract to fit our requirements.

- Please ensure that the publicly deposited datasets are available by publication.

- Please expand the detail on animal husbandry conditions in the Methods section.

- Please adjust the format of the reference list and of the in-text citations according to EMBO Journal format (up to 10 author names in the reference list before et al, DOIs should only be used for preprints and datasets that have not been published yet). Please refer to our Guide to Authors for additional information on EMBO J reference format.

- Please include a separate reference for publicly available datasets that have been reanalyzed in this manuscript.
Please refer to our detailed online guidelines for further information:
https://www.embopress.org/page/journal/14602075/authorguide#referencesformat

- Please rename the supplementary tables to Dataset EV1-Dataset EV23 and upload each separately. The nomenclature should be corrected in all places including the source file names, titles in the system, legend in each file, callouts in the manuscript.

- Please add a title page to the Appendix file with the title (Appendix to [Title]) and a Table of Contents listing each item and its page number. Please update the nomenclature to Appendix Figure S1, etc. from Appendix Figure A1, etc. in all places.

- Please note that EMBO press papers are accompanied online by:
A) a short (2 sentences) summary of the findings and their significance,
B) 2-5 short bullet points highlighting the key results, and
C) a synopsis image in .jpg or .png format that is exactly 550 pixels wide and 300 pixels high. Please note that the text needs to be legible at the final size. Please upload this information along with your revised manuscript (the text for A and B should be provided in one separate Word file uploaded as Cover Art/Synopsis Image).

- Thanks for providing the source data (SD). Please upload the SD file folders, which are currently all combined in a single ZIP archive, as one separate archive per each main figure. Source data files need to be saved in a scheme one figure/folder and then uploaded as .zip files. E.g. all the Source data files for figure 1 need to be saved in a single folder and this needs to be zipped and then uploaded as "SD figure 1.zip" file. For appendix figures, ZIP together all source data and rename to Appendix Fig. S1, etc.

Referee #1:

All my initial comments have been satisfactorily dealt with. Below are a few specific suggestions of alterations within the text to clarify meaning:

1. Introduction sentence:"By in vivo validation, we have identified active ATAC- and HiC-based enhancer sequences driving LEC-confined reporter expression". This doesn't reflect the research described, needs rephrasing.

The line has been rephrased to better match the content of the paper.

2. Results page 7 sentence: "In total about 2500 TADs have been identified solely in LECs and BECs and 4429 were common to the two cell populations. Out of these, differential TAD boundaries were found in association with 272 LEC TADs, 236 BEC TADs and 592 common TADs (Figure 1F)." This is confusingly worded about a key point of the paper. Looking at the relevant figure, I think it can be tightened up as: "In total, both LECs and BECs contain around 2500 TADs that were unique to their subtype, while 4429 were common to both cell populations.

Out of these, differential TAD boundaries were found in association with 272 LEC-only TADs, 236 BEC-only TADs and 592 common TADs (Figure 1F)."

We thank Referee 1 for the suggestion and we implemented it in the text.

3. In the same section as above, they switch between defining shared TADs as "common" and as "consensus". This should be consistent, and clarified more clearly in the text. "Common" would seem like a better definition.

Common and consensus TADs were used to indicate two different concepts, but as indicated here and by a similar comment by Referee 2 the text was evidently not sufficiently clear on the distinction. To aid comprehension, we have renamed "consensus TAD (boundary)" to "reproducible TAD (boundary)".

4. First sentence of page 8 in the next subsection, ".of which LEC-enriched gene loci undergo changes" should be changed to "of which genes with LEC-enriched expression undergo changes in loci chromatin confirmation" or similar (a loci cannot simply be enriched). The second sentence, starting " As LECs and VECs share several genes and developmental dynamics..." could also be clarified: as it stands, why does their shared genes/dynamics mean that their differences must recapitulate those between LECs and general blood ECs??

We agree this line was not clear. It has now been changed to: "To obtain a genome-wide understanding of which genes with LEC-enriched expression undergo changes in loci chromatin conformation, we have characterised the transcriptional signature of LECs and venous endothelial cells (VECs), the subset of BECs more closely related to LECs, by single-cell (sc) RNA-sequencing. As LECs and VECs share several genes and developmental dynamics, this allowed us to identified clear transcriptomic differences between these cell populations without the stronger gene signature coming from arteries."

5. Figure 3D and text describing it: At 2dpf tail images of in situ, gene expression appears to be shown in the vein, with no evidence that expression is in the "lymphatic endothelium". The later timed images, particularly in the Appendix, are convincing. However, could the language describing this be tightened up in legend and text.

We agree the description was imprecise. It has been corrected to: "found that all four genes are expressed in the venous sprouts at 2 dpf and in the lymphatic endothelium at 5 dpf".

6. Figure 4: In Figure 4 B E H K, could the direction of the gene be more clearly labelled please? And in K, could the tested enhancer be marked by arrowhead as in others?

An arrow by the gene name indicating orientation has been added to all gene loci visualizations. We thank Referee 1 for spotting the missing arrow in panel K, which has been re-added.

6b. Related appendix A1G. Some of the Prox1 enhancers appear open in both BECs and LECs but this is hard to tell. Please indicate Prox1 enhancer location on the BEC track, and specifically mention this in the text (since the other LEC enhancers discussed contained LEC-specific peaks.

We agree that in the previous version it was hard to distinguish the difference between ATAC signal in LEC and BEC at the Prox1 enhancers, mainly due to the very zoomed out visualization used. Therefore, we have added panels showing the zoomed-in ATAC signals at each of the Prox1 enhancers. As it can be seen at this resolution, all the prox1 enhancers, with the exception of *+15.2prox1a*, are DA in LECs compared to BECs, as referenced in the text.

Referee #2:

I would like to thank the authors for their thorough point-by-point response. My previous questions and concerns have been fully addressed. I also agree to the changes introduced in the revised manuscript, which improve both the readability and potential significance of the study.

I have only a few minor points and suggestions:

1. The authors might consider using "topologically associating domains" throughout the manuscript and title, as this is a more commonly used term in the literature.

We thank Referee 2 for this suggestion which has been implemented throughout the study.

2. The numbers reported in the manuscript text related to Figure 1E do not appear to match those shown in the figure: "...of which 151 were classified as 'complex' changes, 707 as 'merged/split', 40 as 'shifted', and 217 as 'strength' changes or unclassified (Figure 1E, Table 3)."

We thank Referee 2 for spotting the mismatch. We corrected the numbers to match the ones produced by the final version of the analysis.

3. Related to the new Figure 1G, the terminology could be improved. Are "consensus" and "all" synonymous? Does "all" include differential boundaries? In addition, the figure legend refers to "all boundaries," whereas the main text refers to "boundaries of consensus TADs." More consistent terminology would improve clarity, particularly since TADs and TAD boundaries are distinct concepts, as the authors discuss.

As discussed with Referee 1, we realized that some of our terminology has been quite confusing. We have changed “consensus” to “reproducible” throughout the manuscript.

4. I appreciate the effort and improvements made to visualize differences in TAD organization between LECs and BECs. In Figures 2C and 2D, non-differential LEC TADs are shown in black and differential LEC TADs in orange. The manuscript text refers to the inclusion of a 150 kb region for the tbx1 gene locus and to a large ~1100 kb TAD encompassing prox1a in LECs. However, when looking at the differential TADs in the figure, it is difficult to identify which specific regions are referenced in the text. Two possible suggestions:
a) Indicate the relevant regions described in the main text within the figure panels.
b) Alternatively, consider using the green/purple colour scheme that is used throughout the

manuscript for differential TADs (currently shown in orange), which may help distinguish gains or losses of TADs in LECs relative to BECs more intuitively.

We thank Referee 2 for bringing this to our attention. We have modified the TAD visualization strategy for the indicated panes: differential TADs are now indicated as colored boxed, and TADs spanning the differential boundaries are indicated by grey boxes, while the position of the differential boundaries themselves is indicated by arrowheads.

5. Perhaps I overlooked it, but could the authors provide the list of genes referred to in the following sentence as supplementary information? "In total, we identified 46 LEC-enriched and 39 BEC-enriched genes associated with changing boundaries, corresponding respectively to 29% and 26% of all DE genes."

We thank Referee 2 for catching the missing data, which has now been added in Table 6 and referenced in text.

Typos:

"Therefore, while a generally there is no ..." remove "a"

"... chromatin accessibility are also accompanied by changes in TAD between LECs and BECs ..." change "TAD" to "TADs"

"Only promoters were considered for this analysis as their attribution to a specific gene is more robust that for enhancers ..." change to "more robust than that for enhancers"

Correct "Germline GFP+ enhancer" in Supplementary Table 22

Figure 2 legend: contains previous figure labels "E" and "F"

Figure 2 legend: missing period in "in orange Differential"

All typos listed have been fixed.

Dr. Katarzyna Koltowska
Uppsala University
Department of Immunology, Genetics and Pathology
Sweden

Dear Dr. Koltowska,

I am pleased to inform you that your manuscript has been accepted for publication in EMBO reports. Your manuscript will be processed for publication by EMBO Press. It will be copy edited and you will receive page proofs prior to publication. Please note that you will be contacted by Springer Nature Author Services to complete licensing and payment information.

You may qualify for financial assistance for your publication charges - either via a Springer Nature fully open access agreement or an EMBO initiative. Check your eligibility: https://link.springer.com/journal/44319/how-to-publish-with-us

Should you be planning a Press Release on your article, please notify the production team via this form in order to coordinate publication and release dates: https://forms.cloud.microsoft/e/eH4A8JVP6g

If you have any questions, please do not hesitate to contact the Editorial Office. Thank you for your contribution to EMBO Reports.

Yours sincerely,

Kurt Weir
Editor
EMBO Reports

>>> Please note that it is EMBO Reports policy for the transcript of the editorial process (containing referee reports and your response letter) to be published as an online supplement to each paper. If you do NOT want this, you will need to inform the Editorial Office via email immediately. More information is available here: https://link.springer.com/partners/embo-press/editorial-policies#Peer%20review

EMBO Press Author Checklist

Corresponding Author Name: Katarzyna Koltowska	
Journal Submitted to: EMBO Reports	
Manuscript Number: EMBOR-2025-62876V1-T	

USEFUL LINKS FOR COMPLETING THIS FORM
The EMBO Journal - Author Guidelines
EMBO Reports - Author Guidelines
Molecular Systems Biology - Author Guidelines
EMBO Molecular Medicine - Author Guidelines

Reporting Checklist for Life Science Articles (updated January
This checklist is adapted from Materials Design Analysis Reporting (MDAR) Checklist for Authors. MDAR establishes a minimum set of requirements in transparent reporting in the life sciences (see Statement of Task: 10.31222/osf.io/9sm4x). Please follow the journal's guidelines in preparing your
Please note that a copy of this checklist will be published alongside your article.

Abridged guidelines for figures
1. Data
The data shown in figures should satisfy the following conditions:
- ➞ the data were obtained and processed according to the field's best practice and are presented to reflect the results of the experiments in an accurate and unbiased manner.
- ➞ ideally, figure panels should include only measurements that are directly comparable to each other and obtained with the same assay.
- ➞ plots include clearly labeled error bars for independent experiments and sample sizes. Unless justified, error bars should not be shown for technical
- ➞ if n<5, the individual data points from each experiment should be plotted. Any statistical test employed should be justified.
- ➞ Source Data should be included to report the data underlying figures according to the guidelines set out in the authorship guidelines on Data

2. Captions
Each figure caption should contain the following information, for each panel where they are relevant:
- ➞ a specification of the experimental system investigated (eg cell line, species name).
- ➞ the assay(s) and method(s) used to carry out the reported observations and measurements.
- ➞ an explicit mention of the biological and chemical entity(ies) that are being measured.
- ➞ an explicit mention of the biological and chemical entity(ies) that are altered/varied/perturbed in a controlled manner.
- ➞ the exact sample size (n) for each experimental group/condition, given as a number, not a range;
- ➞ a description of the sample collection allowing the reader to understand whether the samples represent technical or biological replicates (including how many animals, litters, cultures, etc.).
- ➞ a statement of how many times the experiment shown was independently replicated in the laboratory.
- ➞ definitions of statistical methods and measures:
  - common tests, such as t-test (please specify whether paired vs. unpaired), simple χ2 tests, Wilcoxon and Mann-Whitney tests, can be unambiguously identified by name only, but more complex techniques should be described in the methods section;
  - are tests one-sided or two-sided?
  - are there adjustments for multiple comparisons?
  - exact statistical test results, e.g., P values = x but not P values < x;
  - definition of 'center values' as median or average;
  - definition of error bars as s.d. or s.e.m.

Please complete ALL of the questions below.
Select "Not Applicable" only when the requested information is not relevant for your study.

Materials

Newly Created Materials	Information included in the manuscript?	In which section is the information available? (Reagents and Tools Table, Materials and Methods, Figures, Data Availability Section)
New materials and reagents need to be available; do any restrictions apply?	Yes	Data availability

Antibodies	Information included in the manuscript?	In which section is the information available? (Reagents and Tools Table, Materials and Methods, Figures, Data Availability Section)
For **antibodies** provide the following information:<br>- Commercial antibodies: RRID (if possible) or supplier name, catalogue number and or/clone number<br>- Non-commercial: RRID or citation	Yes	Reagents and Tools Table

DNA and RNA sequences	Information included in the manuscript?	In which section is the information available? (Reagents and Tools Table, Materials and Methods, Figures, Data Availability Section)
**Short novel DNA or RNA including primers, probes**: provide the sequences.	Yes	Reagents and Tools Table

Cell materials	Information included in the manuscript?	In which section is the information available? (Reagents and Tools Table, Materials and Methods, Figures, Data Availability Section)
**Cell lines:** Provide species information, strain. Provide accession number in repository **OR** supplier name, catalog number, clone number, and/**OR** RRID.	Not Applicable	
**Primary cultures:** Provide species, strain, sex of origin, genetic modification status.	Not Applicable	
Report if the cell lines were recently **authenticated** (e.g., by STR profiling) and tested for mycoplasma contamination.	Not Applicable	

Experimental animals	Information included in the manuscript?	In which section is the information available? (Reagents and Tools Table, Materials and Methods, Figures, Data Availability Section)
**Laboratory animals or Model organisms:** Provide species, strain, sex, age, genetic modification status. Provide accession number in repository **OR** supplier name, catalog number, clone number, **OR** RRID.	Yes	Reagents and Tools table (sex not determined)
**Animal observed in or captured from the field:** Provide species, sex, and age where possible.	Not Applicable	
Please detail **housing and husbandry conditions**.	Yes	Materials and Methods

Plants and microbes	Information included in the manuscript?	In which section is the information available? (Reagents and Tools Table, Materials and Methods, Figures, Data Availability Section)
**Plants:** provide species and strain, ecotype and cultivar where relevant, unique accession number if available, and source (including location for collected wild specimens).	Not Applicable	
**Microbes:** provide species and strain, unique accession number if available, and source.	Not Applicable	

Human research participants	Information included in the manuscript?	In which section is the information available? (Reagents and Tools Table, Materials and Methods, Figures, Data Availability Section)
If collected and within the bounds of privacy constraints report on age, sex and gender or ethnicity for all study participants.	Not Applicable	

Core facilities	Information included in the manuscript?	In which section is the information available? (Reagents and Tools Table, Materials and Methods, Figures, Data Availability Section)
If your work benefited from core facilities, was their service mentioned in the acknowledgments section?	Yes	Acknowledgments

Design

Study protocol	Information included in the manuscript?	In which section is the information available? (Reagents and Tools Table, Materials and Methods, Figures, Data Availability Section)
If study protocol has been **pre-registered, provide DOI in the manuscript**. For clinical trials, provide the trial registration number **OR** cite DOI.	Not Applicable	
Report the **clinical trial registration number** (at ClinicalTrials.gov or equivalent), where applicable.	Not Applicable	

Laboratory protocol	Information included in the manuscript?	In which section is the information available? (Reagents and Tools Table, Materials and Methods, Figures, Data Availability Section)
Provide DOI OR other citation details if **external detailed step-by-step protocols** are available.	Yes	Materials and Methods

Experimental study design and statistics	Information included in the manuscript?	In which section is the information available? (Reagents and Tools Table, Materials and Methods, Figures, Data Availability Section)
Include a statement about **sample size** estimate even if no statistical methods were used.	Yes	Materials and methods, Figure Legends
Were any steps taken to minimize the effects of subjective bias when allocating animals/samples to treatment (e.g. **randomization procedure**)? If yes, have they been described?	Not Applicable	
Include a statement about **blinding** even if no blinding was done.	Yes	Materials and Methods
Describe **inclusion/exclusion criteria** if samples or animals were excluded from the analysis. Were the criteria pre-established?<br><br>If sample or data points were omitted from analysis, report if this was due to attrition or intentional exclusion and provide justification.	Yes	Results
For every figure, are **statistical tests** justified as appropriate? Do the data meet the assumptions of the tests (e.g., normal distribution)? Describe any methods used to assess it. Is there an estimate of variation within each group of data? Is the variance similar between the groups that are being statistically compared?	Yes	Materials and Methods

Sample definition and in-laboratory replication	Information included in the manuscript?	In which section is the information available? (Reagents and Tools Table, Materials and Methods, Figures, Data Availability Section)
In the figure legends: state number of times the experiment was **replicated** in laboratory.	Yes	Legends
In the figure legends: define whether data describe **technical or biological replicates**.	Yes	Legends

Ethics

Ethics	Information included in the manuscript?	In which section is the information available? (Reagents and Tools Table, Materials and Methods, Figures, Data Availability Section)
Studies involving **human participants**: State details of **authority granting ethics approval** (IRB or equivalent committee(s), provide reference number for approval.	Not Applicable	
Studies involving **human participants**: Include a statement confirming that **informed consent** was obtained from all subjects and that the experiments conformed to the principles set out in the WMA Declaration of Helsinki and the Department of Health and Human Services Belmont Report.	Not Applicable	
Studies involving **human participants:** For publication of **patient photos**, include a statement confirming that consent to publish was obtained.	Not Applicable	
Studies involving experimental **animals**: State details of **authority granting ethics approval** (IRB or equivalent committee(s), provide reference number for approval. Include a statement of compliance with ethical regulations.	Yes	Materials and Methods
Studies involving **specimen and field samples:** State if relevant **permits** obtained, provide details of authority approving study; if none were required, explain why.	Not Applicable	

Dual Use Research of Concern (DURC)	Information included in the manuscript?	In which section is the information available? (Reagents and Tools Table, Materials and Methods, Figures, Data Availability Section)
Could your study fall under dual use research restrictions? Please check biosecurity documents and list of **select agents and toxins** (CDC): https://www.selectagents.gov/sat/list.htm	Not Applicable	
If you used a select agent, is the security level of the lab appropriate and reported in the manuscript?	Not Applicable	
If a study is subject to dual use research of concern regulations, is the name of the **authority granting approval and reference number** for the regulatory approval provided in the manuscript?	Not Applicable	

Reporting

The MDAR framework recommends adoption of discipline-specific guidelines, established and endorsed through community initiatives. Journals have their own policy about requiring specific guidelines and recommendations to complement MDAR.

Adherence to community standards	Information included in the manuscript?	In which section is the information available? (Reagents and Tools Table, Materials and Methods, Figures, Data Availability Section)
State if relevant guidelines or checklists (e.g., **ICMJE, MIBBI, ARRIVE, PRISMA**) have been followed or provided.	Yes	Results, Material and Methods, Legends
For **tumor marker prognostic studies,** we recommend that you follow the **REMARK** reporting guidelines (see link list at top right). See author guidelines, under 'Reporting Guidelines'. Please confirm you have followed these guidelines.	Not Applicable	
For **phase II and III randomized controlled trials**, please refer to the **CONSORT** flow diagram (see link list at top right) and submit the CONSORT checklist (see link list at top right) with your submission. See author guidelines, under 'Reporting Guidelines'. Please confirm you have submitted this list.	Not Applicable	

Data Availability

Data availability	Information included in the manuscript?	In which section is the information available? (Reagents and Tools Table, Materials and Methods, Figures, Data Availability Section)
Have **primary datasets** been deposited according to the journal's guidelines (see 'Data Deposition' section) and the respective accession numbers provided in the Data Availability Section?	Yes	Data availability
Were **human clinical and genomic datasets** deposited in a public access-controlled repository in accordance to ethical obligations to the patients and to the applicable consent agreement?	Not Applicable	
Are **computational models** that are central and integral to a study available without restrictions in a machine-readable form? Were the relevant accession numbers or links provided?	Yes	Data availability
If publicly available data were reused, provide the respective **data citations in the reference list**.	Yes	Results
