# Supplementary material for: 3D chromatin organisation contributes to the regulatory logic in differentiating lymphatic endothelium
Source: EMBO Rep. 2026 Aug 14;27(18):5262–91. doi: 10.1038/s44319-026-00895-1 (PMC13601605; doi:10.1038/s44319-026-00895-1)
Supplement: Supplementary file 3 [file 44319_2026_895_MOESM35_ESM.pdf]

Expanded View Figures

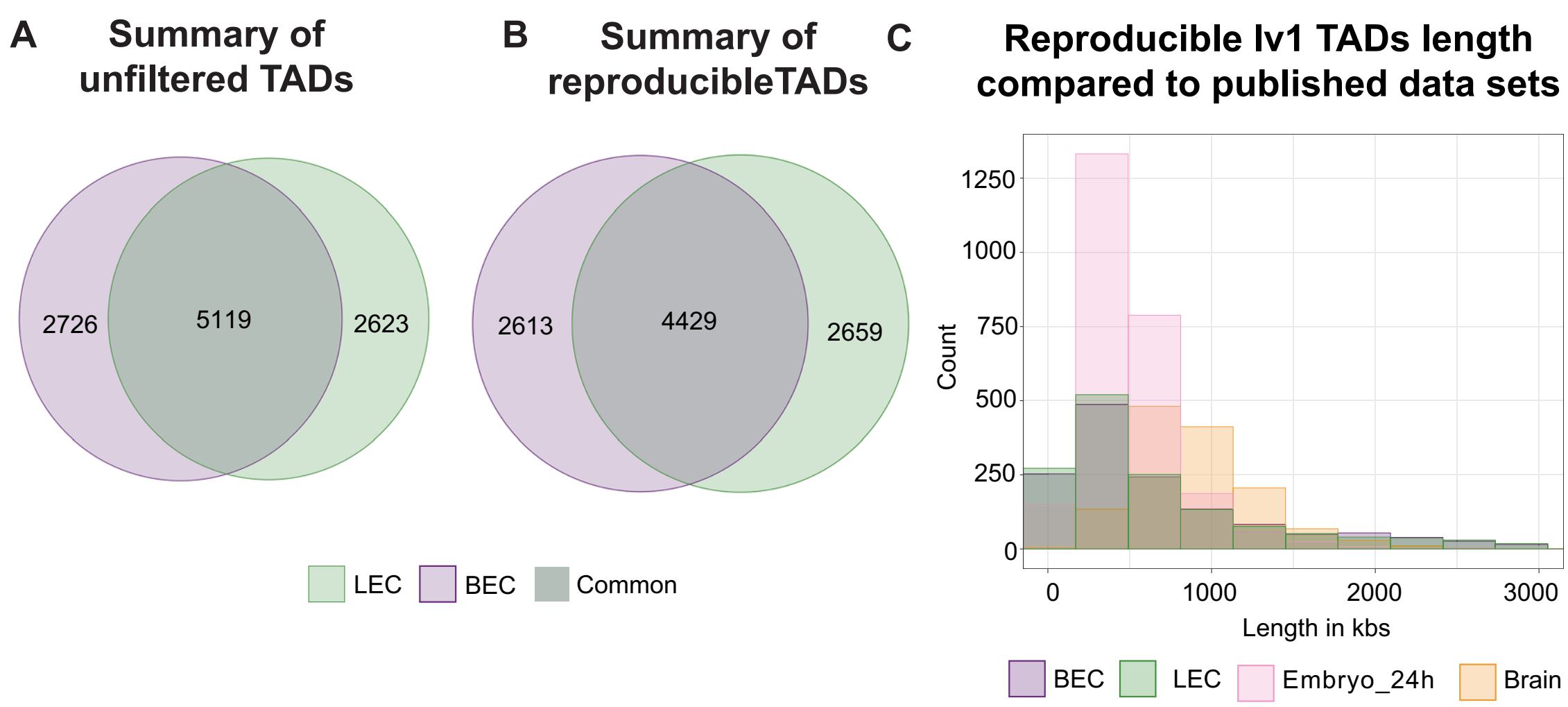

Figure EV1. TAD structure differs between LECs and BECs.

(**A**) Summary of unfiltered TADs of all levels in LECs and BECs. (**B**) Summary of reproducible TADs of all levels in LECs and BECs. (**C**) Length distribution of level 1 TAD compared with published datasets, showing similarities to reported brain data. Purple: BEC TADs; Green: LEC TADs; pink: 24 hpf embryo TADs from (Franke et al, 2021); yellow: brain TADs from Yang et al, (2020)

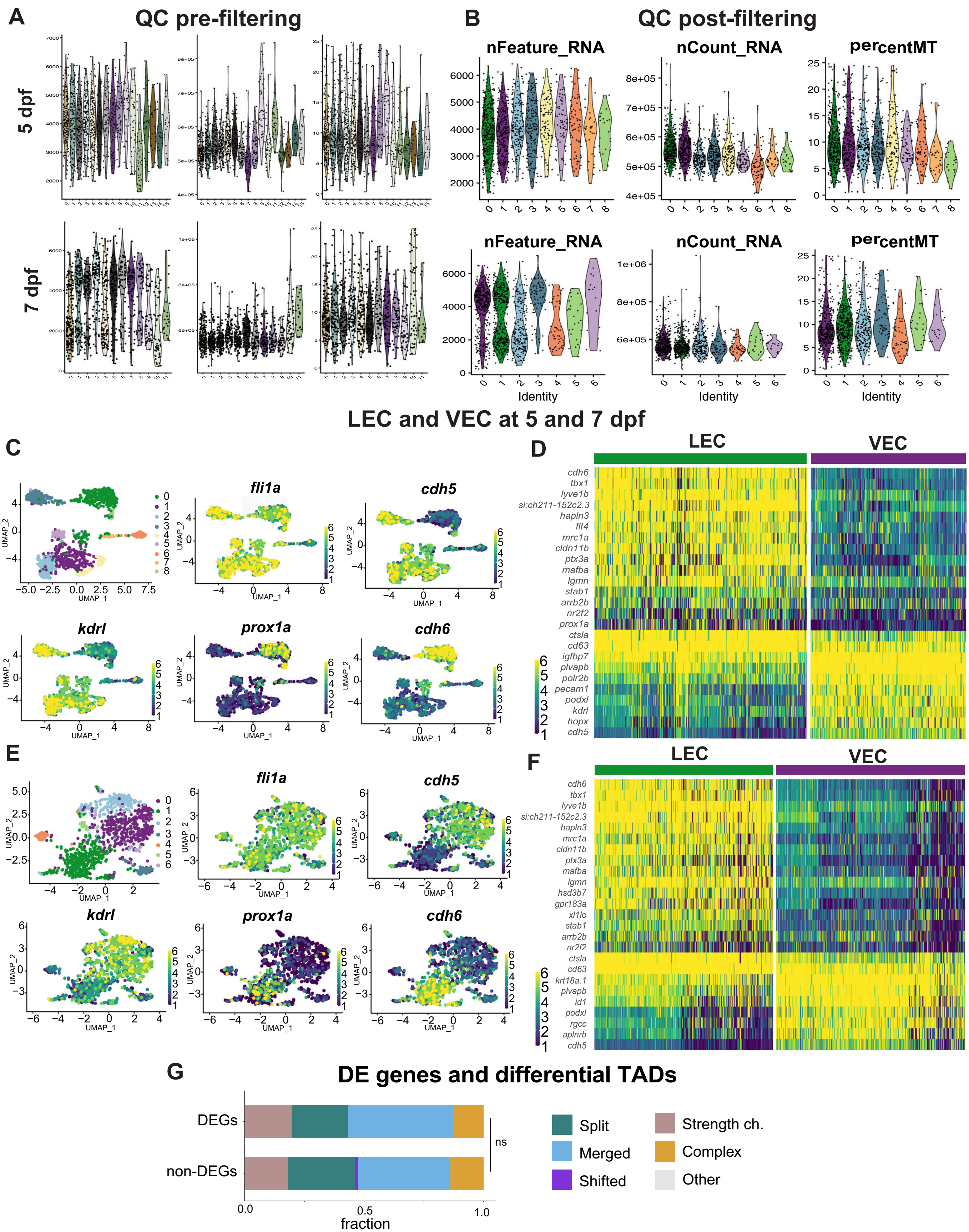
A
QC pre-filtering
5 dpf
7 dpf
B
QC post-filtering
nFeature_RNA
nCount_RNA
percentMT
Identity
LEC and VEC at 5 and 7 dpf
C
fli1a
cdh5
kdrl
prox1a
cdh6
UMAP_1
UMAP_2
D
LEC
VEC
cdh6
tbx1
lyve1b
si:ch211-152c2.3
hapln3
flt4
mrc1a
cldn11b
ptx3a
mafba
lgmn
stab1
arrb2b
nr2f2
prox1a
ctsla
cd63
igfbp7
plvapb
polr2b
pecam1
podxl
kdrl
hopx
cdh5
E
F
hsd3b7
gpr183a
xl1lo
krt18a.1
id1
rgcc
aplnrb
G
DE genes and differential TADs
DEGs
non-DEGs
ns
fraction
Split
Merged
Shifted
Strength ch.
Complex
Other

 © The Author(s)

Figure EV2. scRNA-seq quality control and cluster identification.

(**A**, **B**) Plate quality control (QC) before (**A**) and after (**B**) filtering for low-quality reads, showing the percentage of mitochondrial, protein-coding and rRNA reads. (**C**, **E**) UMAP visualisation of vascular clusters from 5 dpf (**C**) and 7 dpf (**E**), coloured by cluster identity. UMAP plot shows expression of vascular markers: *fli1a* (all endothelium), *chd5* and *kdrl* (blood endothelium), *cdh6* and *prox1a* (lymphatic endothelium). Colour scale shows log-normalised expression. (**D**, **F**) Heatmaps of known gene expression in the endothelial cells at 5 dpf (**D**) and 7 dpf (**F**). Cells ordered by clusters. Colour scale shows log-normalised read counts. (**G**) Distribution of differential TAD boundaries in differentially expressed vs non-differentially expressed genes within level 1 TADs containing a differential boundary. DEG-associated boundaries: $n = 157$; non-DEG-associated boundaries: $n = 1186$. Chi-squared test, ns no significance ($p = 0.2424$). Source data are available online for this figure.

© The Author(s)

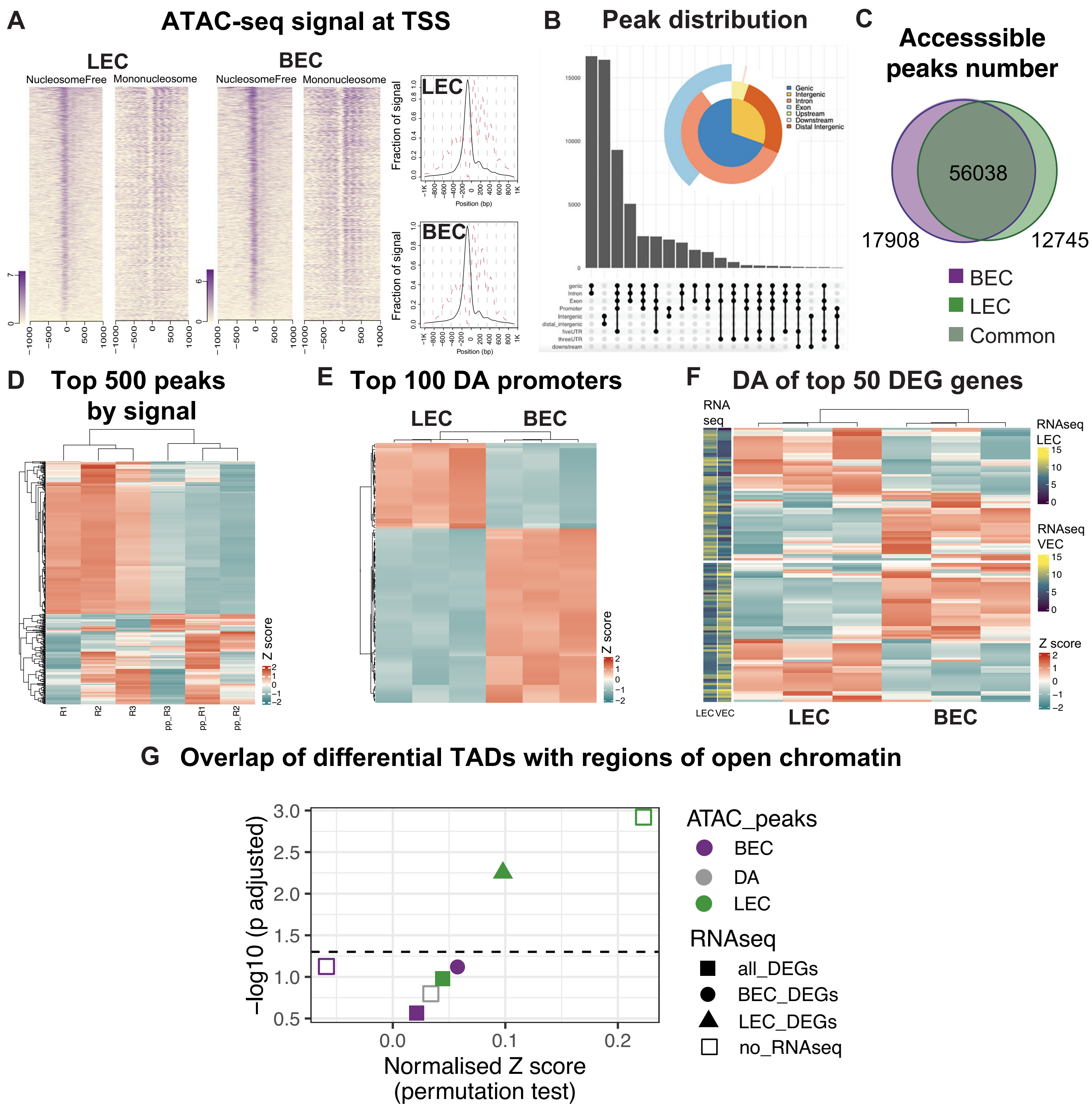

Figure EV3. Combination of ATAC-sequencing and scRNA-sequencing reveals novel LEC factors.

(**A**) ATAC-seq signal at TSS in nucleosome-free and mononucleosome fractions. Left: heatmaps of scaled counts in 10 bp bins centred on TSS. Right: cumulative TSS signal. (**B**) Distribution of genomic feature annotation of identified ATAC-seq peaks. (**C**) Quantification of the tissue-specific and common accessible ATAC-seq peaks. (**D**) ATAC-seq heatmap showing chromatin state at the top 500 DA peaks. (**E**) ATAC-seq heatmap of the top 100 differentially accessible (DA) promoters at 5 dpf. (**F**) ATAC-seq heatmap of promoters of differentially expressed (DE) genes at 5 dpf. (**G**) Graphical representation of the results of the permutation tests for the association of TADs, open chromatin regions and differential gene expression. Purple: BEC DA regions; green: LEC DA regions, grey: all DA regions; square: regions associated with any DEG; circle: regions associated with BEC DEG, triangle: regions associated with LEC DEGs, empty square: all accessible regions regardless of DEG association. The significance of these overlaps was assessed by permutation tests, where TAD regions were randomised. The horizontal line represents a significance cutoff $p = 0.05$.

 © The Author(s)

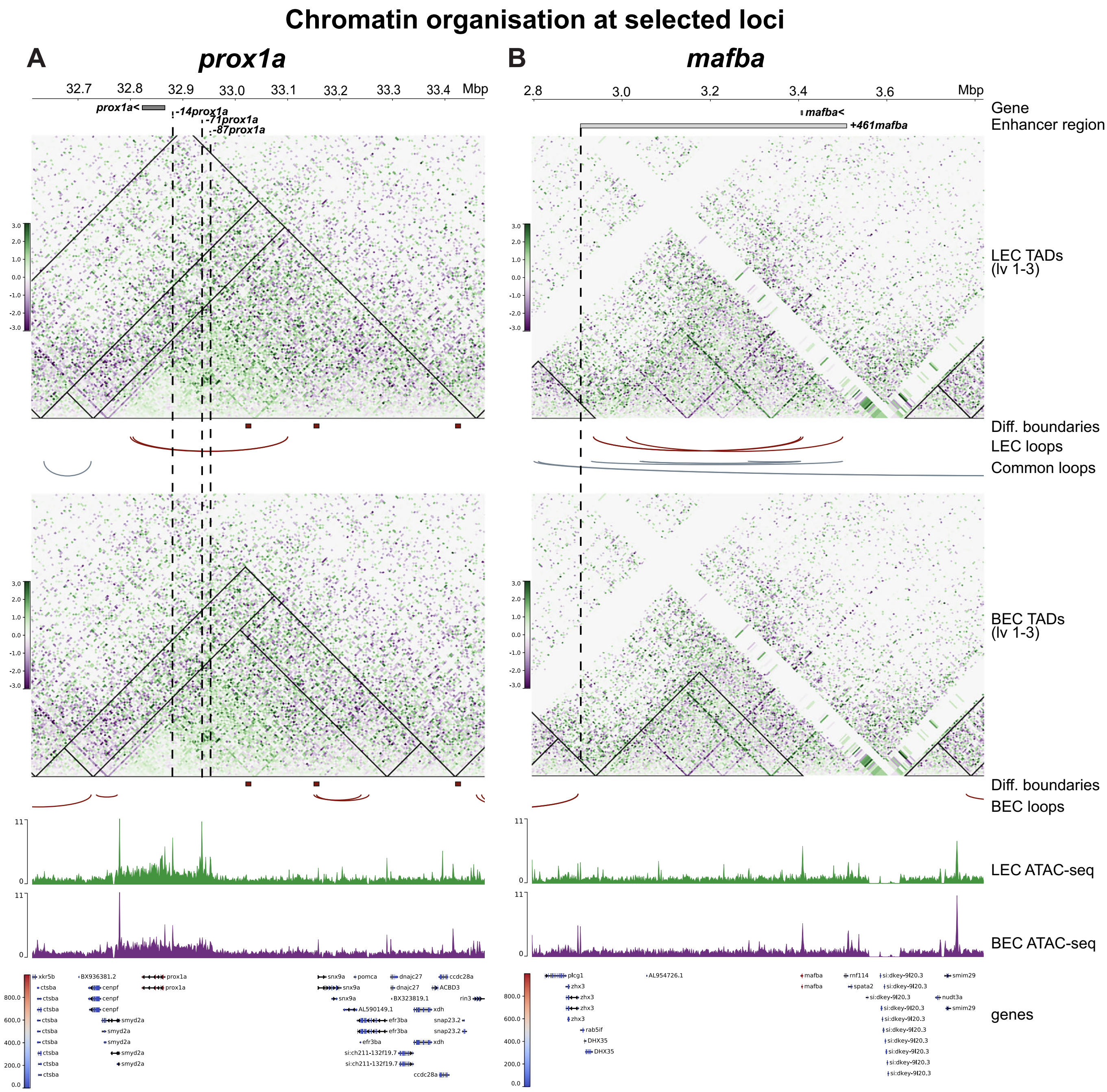

Figure EV4. Characterisation of chromatin state at key lymphatic loci.

(**A**, **B**) Chromatin organisation at the *prox1a* (**A**) and *mafba* (**B**) loci in LECs and BECs. Log2 of the HiC signal in LEC vs BEC is shown. LEC and BEC TADs of levels 1–3 are plotted in black. Differential boundaries are shown in red. The dotted line indicates the position of the putative enhancers. Tissue-specific loops plotted in red. Common loops plotted in grey. Chromatin accessibility plotted in green for LECs and purple for BECs.

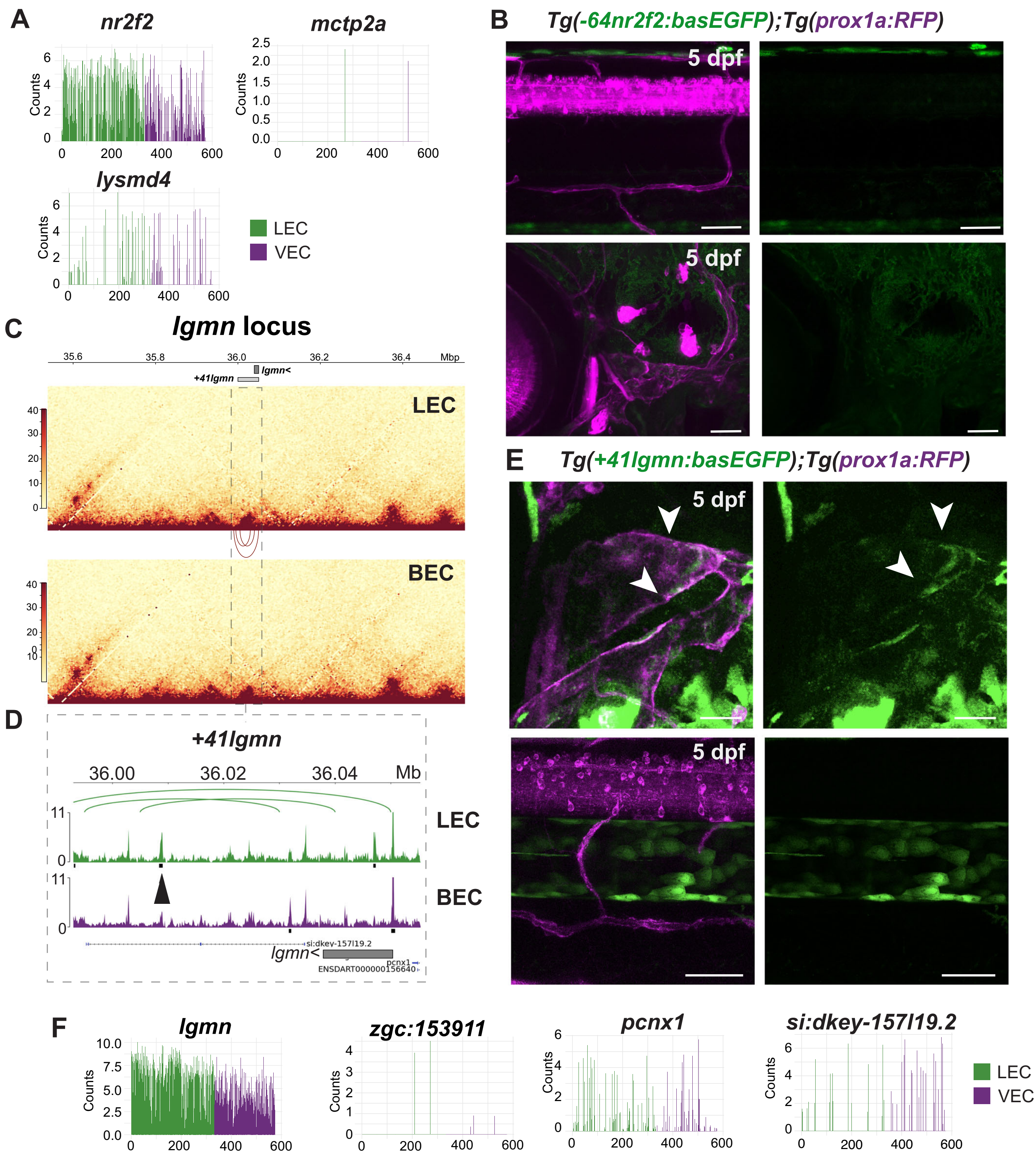

Figure EV5. Additional characterisation of novel HiC-based LEC enhancers.

(**A**) Cell counts of gene loci surrounding *nr2f2* in LEC and VEC clusters at 5 dpf, showing enriched expression at selected loci. (**B**) *Tg(+62nr2f2:basEGFP);Tg(prox1a:RFP)* embryo, showing no enhancer activity in lymphatics at 5 dpf. (**C**) HiC maps in LECs and BECs showing tissue-specific interactions at the *lgmn locus*. Dotted box: regions in (**E**). (**D**) Chromatin accessibility and tissue-specific loops at the *lgmn* locus and *+41lgmn* in LECs and BECs. Black boxes: DA peaks. Arrow: *+41lgmn*. (**E**) *Tg(+41lgmn:basEGFP);Tg(prox1a:RFP)* confocal and masked expression, showing activity in the facial lymphatics at 5 dpf (arrows). (**F**) Cell counts of gene loci around *lgmn* in LEC and VEC clusters at 5 dpf, showing enriched expression at selected loci. Scale bars: 50 μm. Source data are available online for this figure.

 © The Author(s)
